# Supplementary material for: Human milk oligosaccharide mediates mutualism between Escherichia coli and Bifidobacterium bifidum
Source: Nat Commun. 2026 Apr 22;17:3489. doi: 10.1038/s41467-026-71764-7 (PMC13103366; doi:10.1038/s41467-026-71764-7)
Supplement: Supplementary file 1 — Supplementary information [file 41467_2026_71764_MOESM1_ESM.pdf]

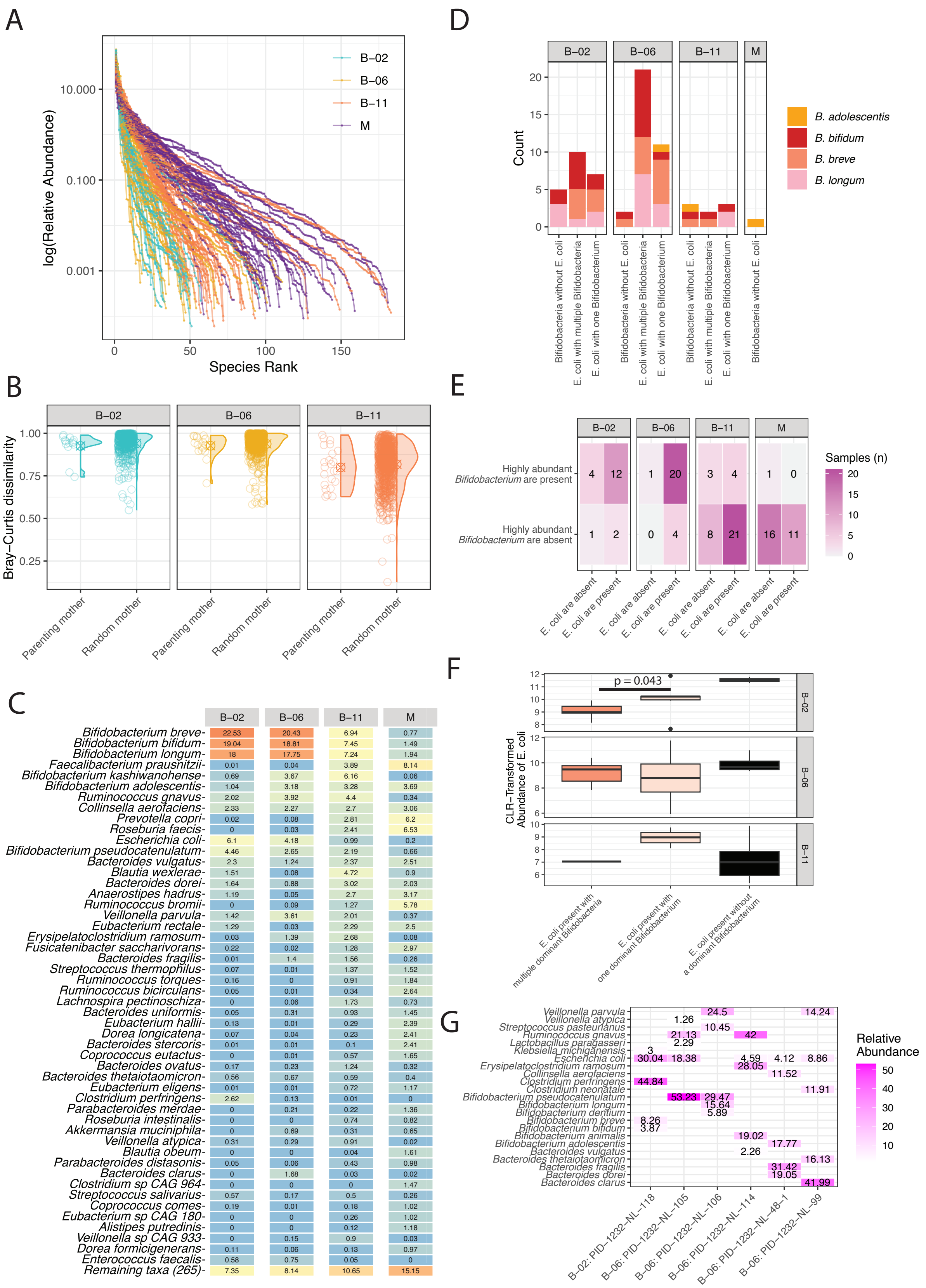

Supplementary Figure 1:

A) Rarefaction curves illustrating observed diversity in maternal and infant samples. B) Boxplots summarizing Bray-Curtis distances of pairwise comparisons between microbiota of infants and parenting or non-related mothers. C) Heatmap displaying mean average

abundance of species in given age groups. Blue colour indicates low mean average abundance; orange colour indicates high mean average abundance. D). Species-level resolved co-occurrence combinations of E. coli and Bifidobacterium taxa (*B. bifidum, B. longum* subsp. *longum*, *B. adolescentis*, and *B. breve*) across samples. E) Age-stratified 2×2 heatmaps of *E. coli* (>0.1% relative abundance) versus highly-abundant *Bifidobacterium* (including *B. bifidum*, *B. breve*, *B. longum*, and *B. adolescentis*; either one or multiple are >20%), showing sample counts per presence/absence combination F) Centered log-ratio (CLR)-transformed abundance of *E. coli* in respective colonization groups (displayed p-value is not corrected for multiple testing). G) Relative abundance of microbial species in samples without an abundant *Bifidobacterium*. Box plots show group median and interquartile range. ⊗ in violin plots indicate group median.

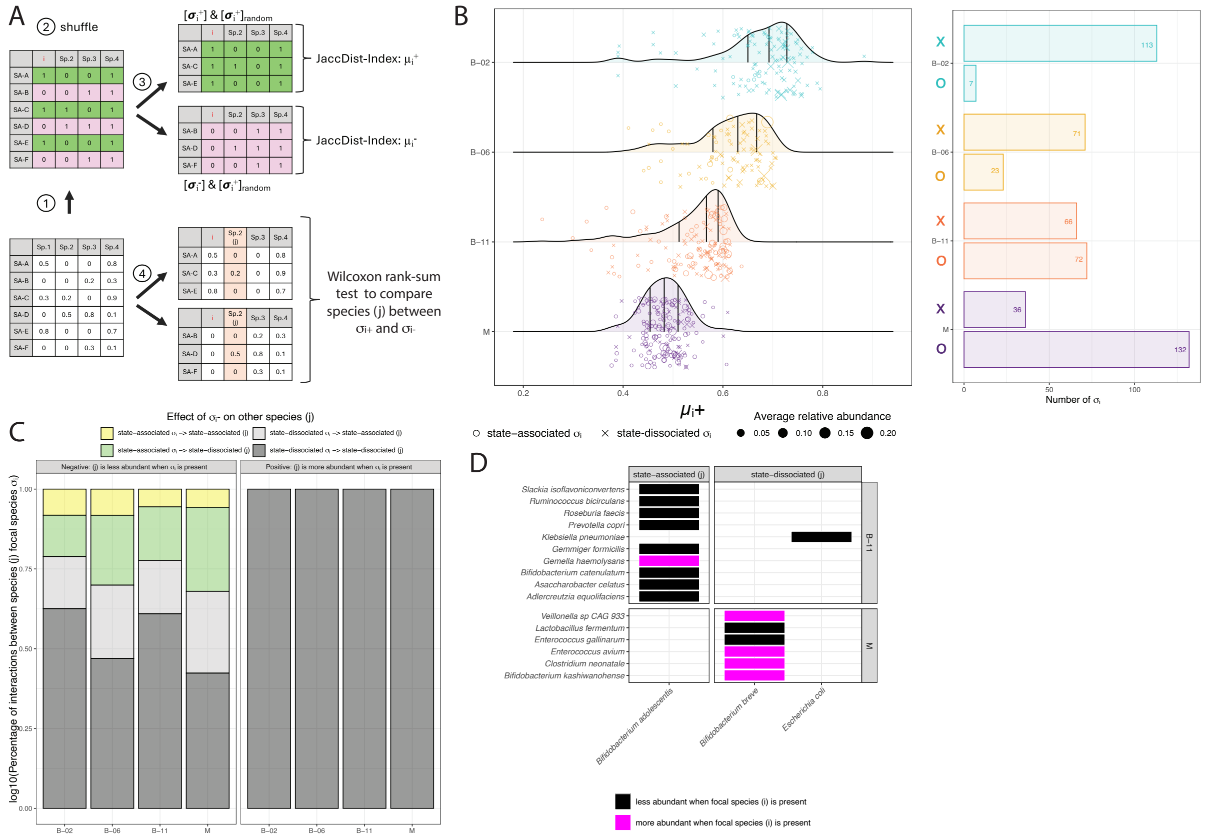

Supplementary Figure 2:

A) Schematic illustration of computational pipeline. B) Distribution of average Jaccard dissimilarities (μ) in presence of state-associated (X) or state-dissociated (O) focal species (***σ***i). C) For each focal species (***σ***i), we identify differences in abundance patterns of any other species (j) between samples where focal species i (***σ***i+) is present and samples where it's absent (***σ***i). Bar-chart summarizes these significant differences. D) Heatmap listing significant associations between 'state-associated' focal species (***σ***i) and any type of species (j) with involvements of highly-abundant *Bifidobacterium* (B. adolescentis, B. breve, *B. bifidum*, and *B. longum* subsp*. longum*) or *E coli*.

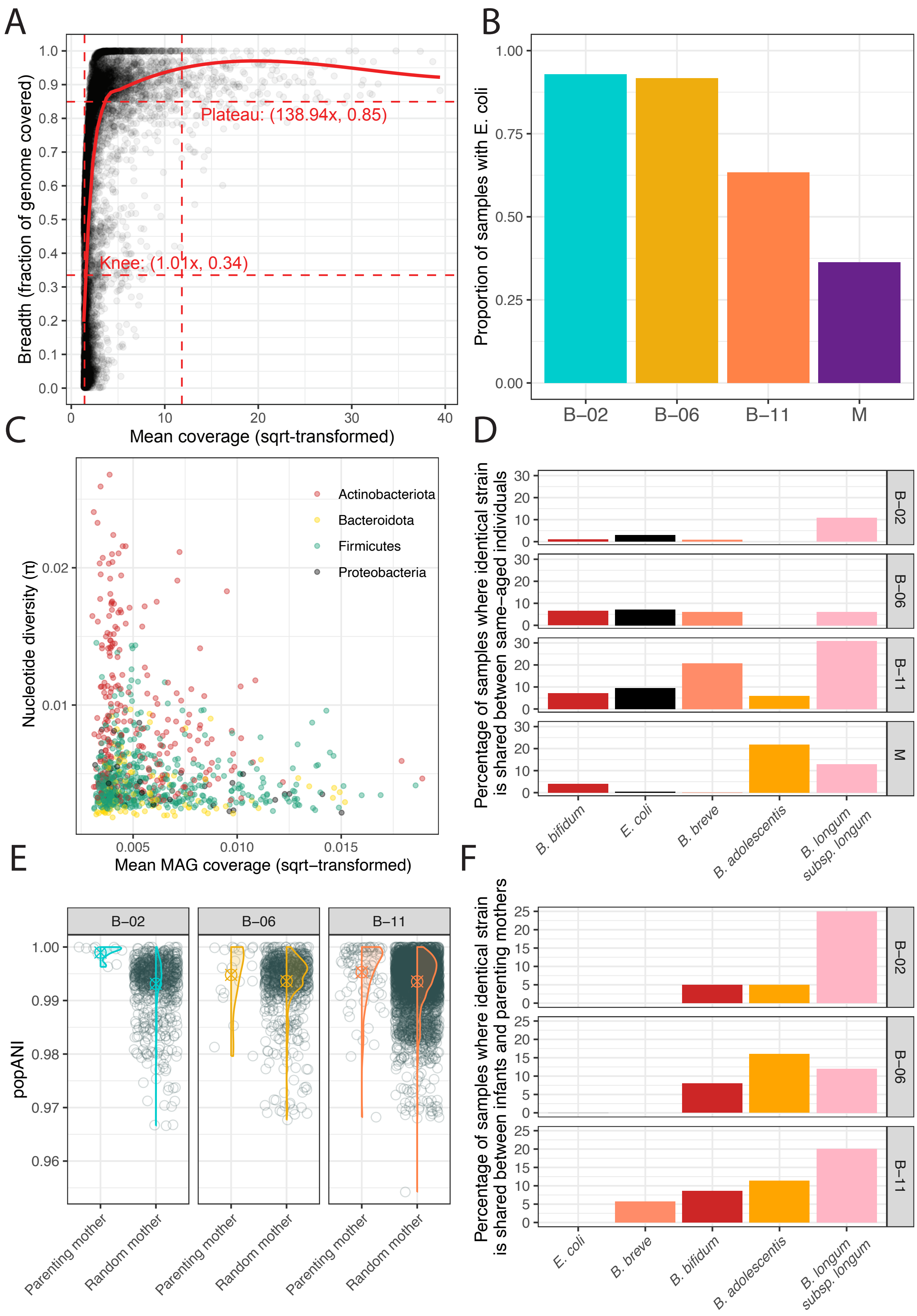

Supplementary Figure 3:

A) Scatterplot showing genome coverage (x-axis) versus genome breadth (y-axis) for dereplicated MAGs. A linear-model smoothing line is overlaid in red, and dotted horizontal lines mark the observed knee (~0.34) and plateau (~0.85) in genome breadth (threshold =

0.5) vs coverage (threshold = 5). B) Bar-chart showing the proportion of maternal (M) and infant samples at 2, 6, and 11 months (B-02, B-06, and B-11) in which *E. coli* MAGs were detected using the thresholds of genome breadth > 0.5 and coverage > 5. C) Scatterplot between nucleotide diversity (π) and mean MAG coverage. Each dot represents a MAG, coloured by corresponding phylum. D) Summary of strain-sharing events where strains of *Bifidobacterium*, and *E. coli* are shared among same-aged individuals. E) Differences of pop-ANI between comparisons of infants with parenting and random mothers. F) Summary of strain-sharing events where identical strains of *Bifidobacterium*, and *E. coli* are shared between infants and parenting mothers.

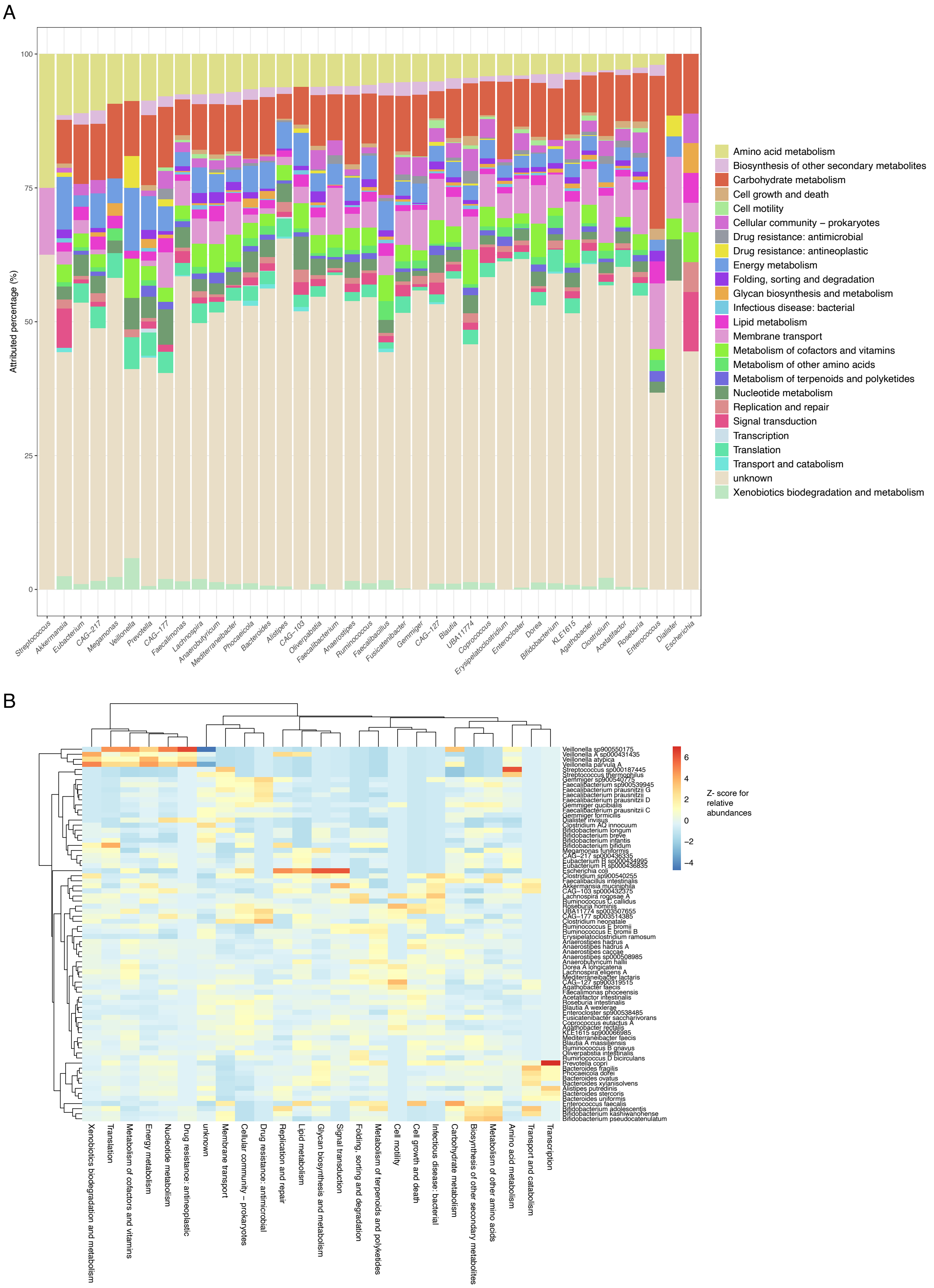

Supplementary Figure 4:

A) Bar-charts summarizing the relative distribution of GH-2 co-evolving genes per genus across given kegg-orthology (ko) groups. Distribution of ko-groups among microbial genera significantly differed from shuffled random distributions. B) Heatmap displaying the scaled proportions (z-scores) of GH-2 co-evolved genes associated with KEGG categories

(columns) across taxa (rows). Red indicates higher proportions, and blue indicates lower proportions compared to the mean. Rows and columns were clustered using Euclidean distance and Ward's D2 method.

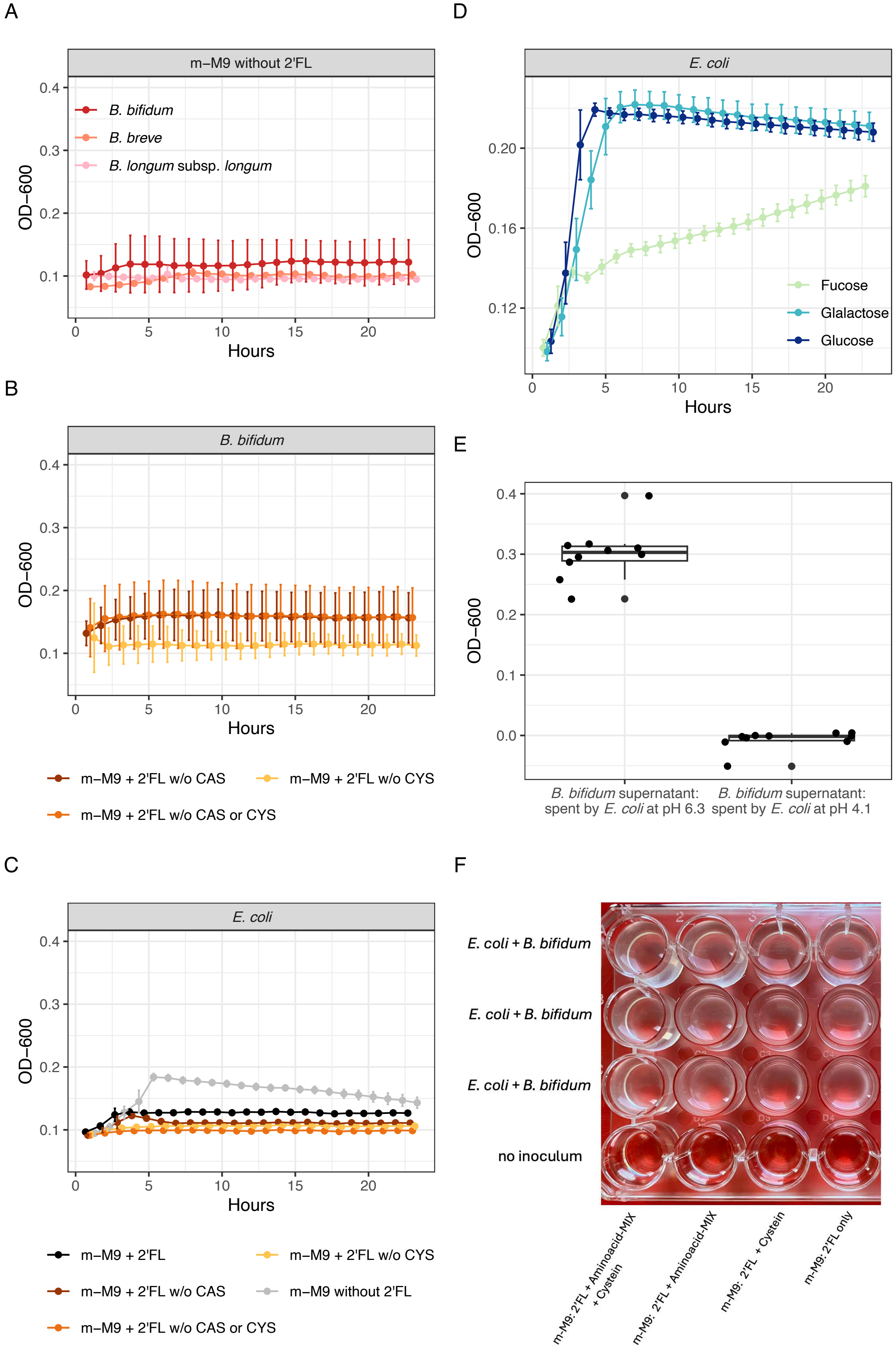

Supplementary Figure 5:

A) No-carbon control: growth of *B. bifidum* (red), *B. breve* (salmon), *B. longum* (pink) in modified M9-medium (m-M9) without 2'-O-fucosyl-lactose (2'FL) (n=3 biological replicates per species), measured by optical density (OD-600). B) No-amino-acid control: growth of B. bifidum in m-M9 + 2'FL lacking cas-amino-acids (brown), cysteine (yellow), or both (orange), measured by OD-600. C) Growth of *E. coli* in m-M9 and respective controls (n=3 biological replicates per condition). D) Growth of *E. coli* on 2'FL monomers fucose (light-green), galactose (light-blue), and glucose (dark-blue) (n=3 biological replicates per condition). E) Growth of *E. coli* in supernatant of m-M9 + 2'FL previously spent by *B. bifidum* at pH 6,3 and 4,1 (n = 10 biological replicates per condition). F) Co-cultures of *E. coli* and *B. bifidum* in m-M9 with and without each additive. Dots in line-plots show group median, error bars show standard deviation.

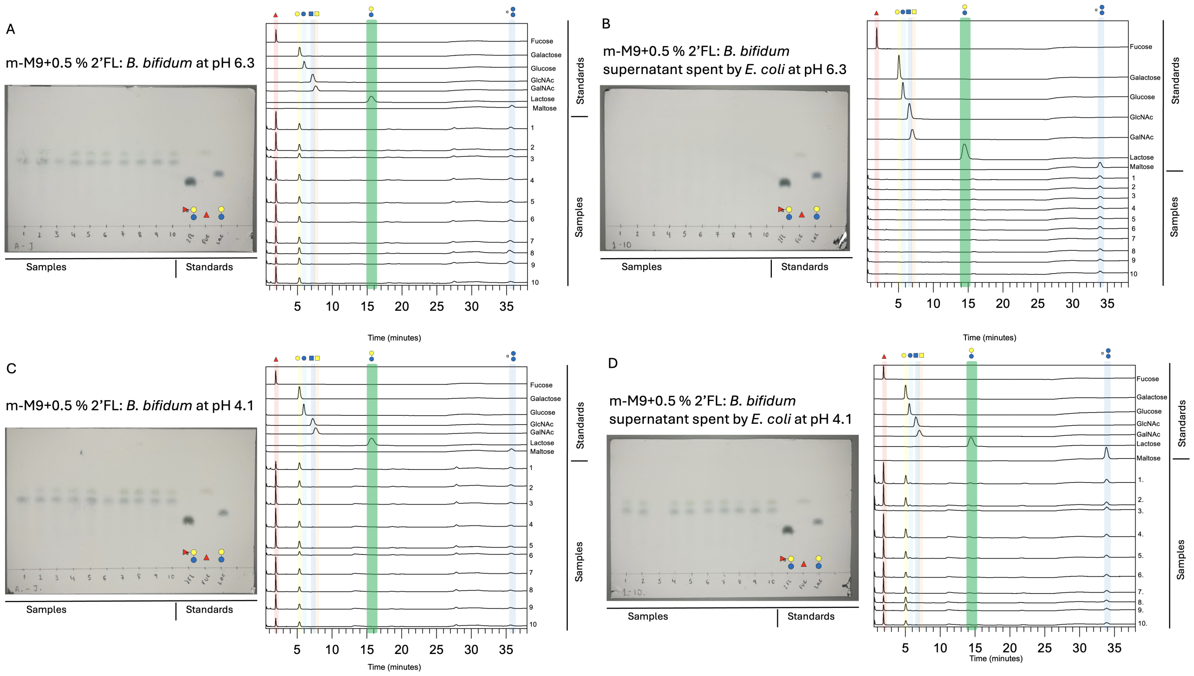

Supplementary Figure 6:
Thin layer chromatography (TLC) and matching high-pressure anion-exchange chromatography (HPAEC) for quantification of 2'-O-fucosyl-lactose (2'FL), its monomers, and lactose. A) Supernatants of *B. bifidum* grown in m-M9 with 0.5% 2'FL at pH 6,3. B) Supernatants of E. coli grown in B. bifidum spent m-M9 with 0.5% 2'FL at pH 6,3. C) Supernatants of B. bifidum grown in m-M9 with 0.5% 2'FL at pH 4,1. D) Supernatants of *E. coli* grown in *B. bifidum* spent m-M9 with 0.5% 2'FL at pH 4,1.
Supplementary File 1: co-culture of *E. coli* and *B. bifidum*: Quantification of Gram stains.
