# Supplementary material for: Human milk oligosaccharide mediates mutualism between Escherichia coli and Bifidobacterium bifidum
Source: Nat Commun. 2026 Apr 22;17:3489. doi: 10.1038/s41467-026-71764-7 (PMC13103366; doi:10.1038/s41467-026-71764-7)
Supplement: Supplementary file 2 — Description of Additional Supplementary Files [file 41467_2026_71764_MOESM2_ESM.pdf]

File Name: Supplementary Data 1
Description: Subject cohort demographics

File Name: Supplementary Data 2:
Description: Abundance distribution of microbiota

File Name: Supplementary Data 3
Description: MAG details and number of observed and predicted GH-2 domain-containing enzymes per MAG

File Name: Supplementary Data 4
Description: Most restricted or loose genes in MAGs of abundant *Bifidobacterium* and *E. coli*

File Name: Supplementary Data 5
Description: Co-culture of *E. coli* and *B. bifidum* - Quantification of Gram stains.
