# Supplementary material for: Human milk oligosaccharide mediates mutualism between Escherichia coli and Bifidobacterium bifidum
Source: Nat Commun. 2026 Apr 22;17:3489. doi: 10.1038/s41467-026-71764-7 (PMC13103366; doi:10.1038/s41467-026-71764-7)
Supplement: Supplementary file 7 — Supplementary Data 5 [file 41467_2026_71764_MOESM7_ESM.pdf]

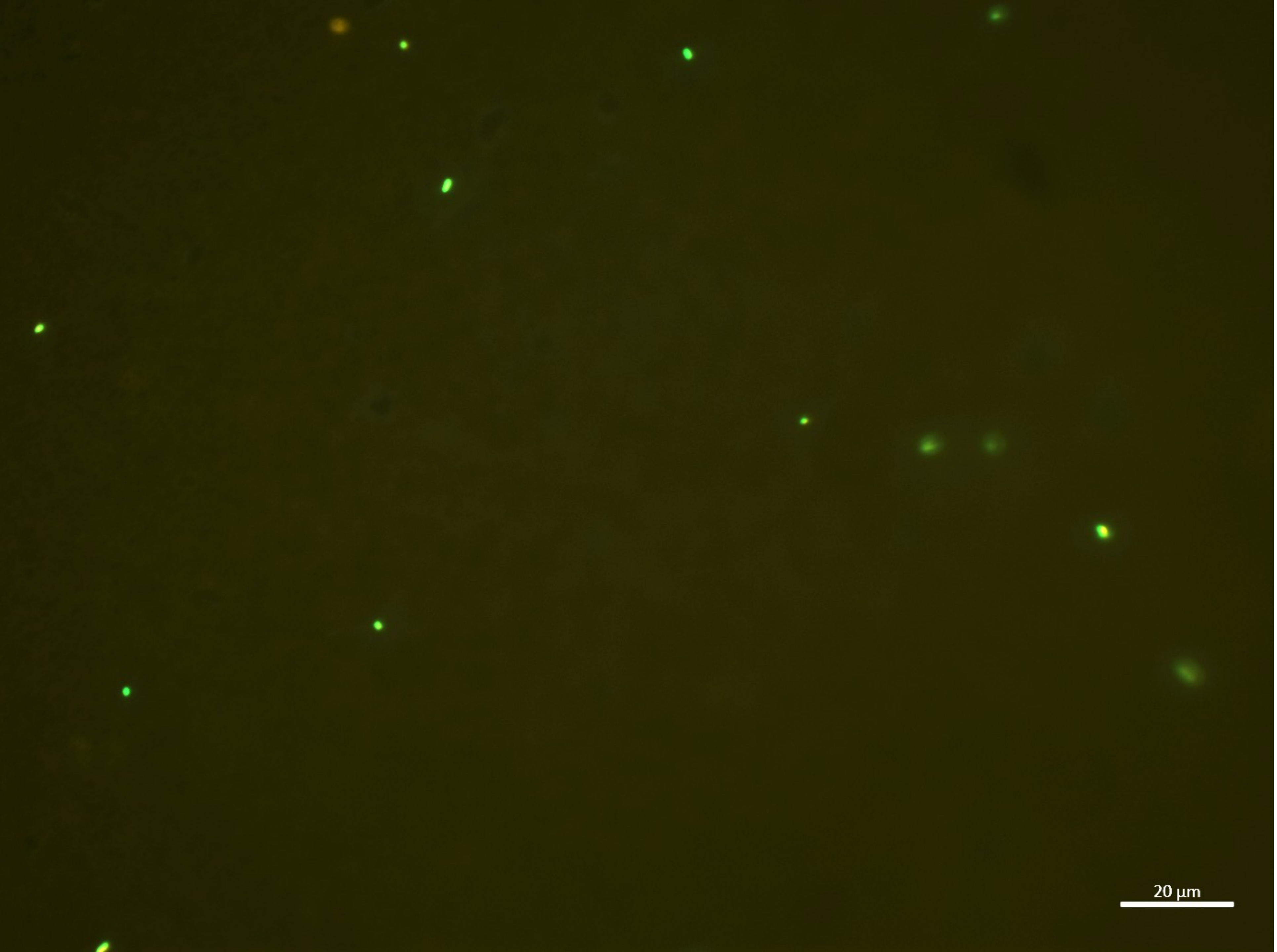

14:00-A

B.bifidum	1
E.coli	13
Sum	14

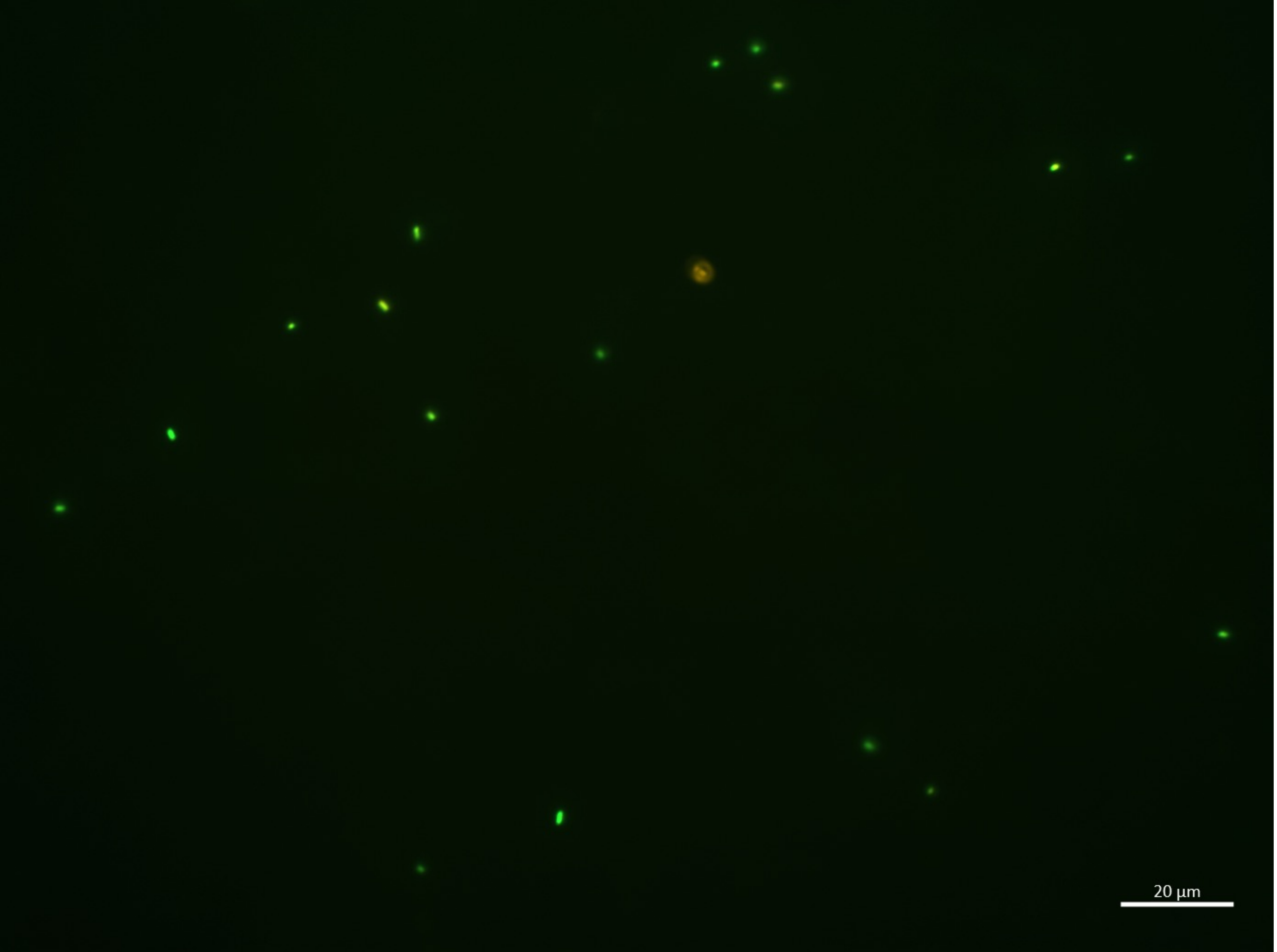

14:00-B

**B.bifidum**	**1**
**E.coli**	17
**Sum**	18

14:00-C

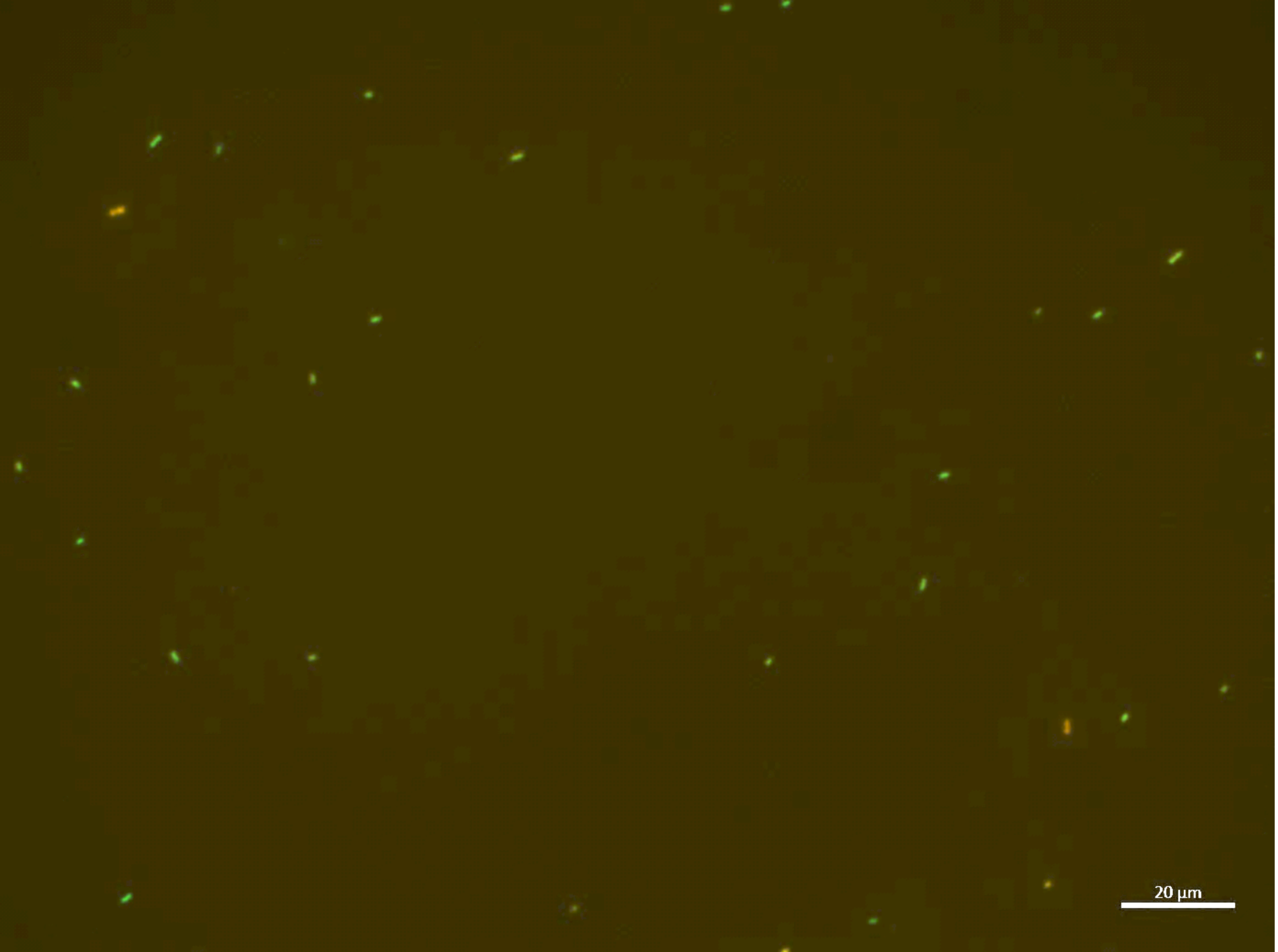

**B.bifidum**	**3**
**E.coli**	25
**Sum**	28

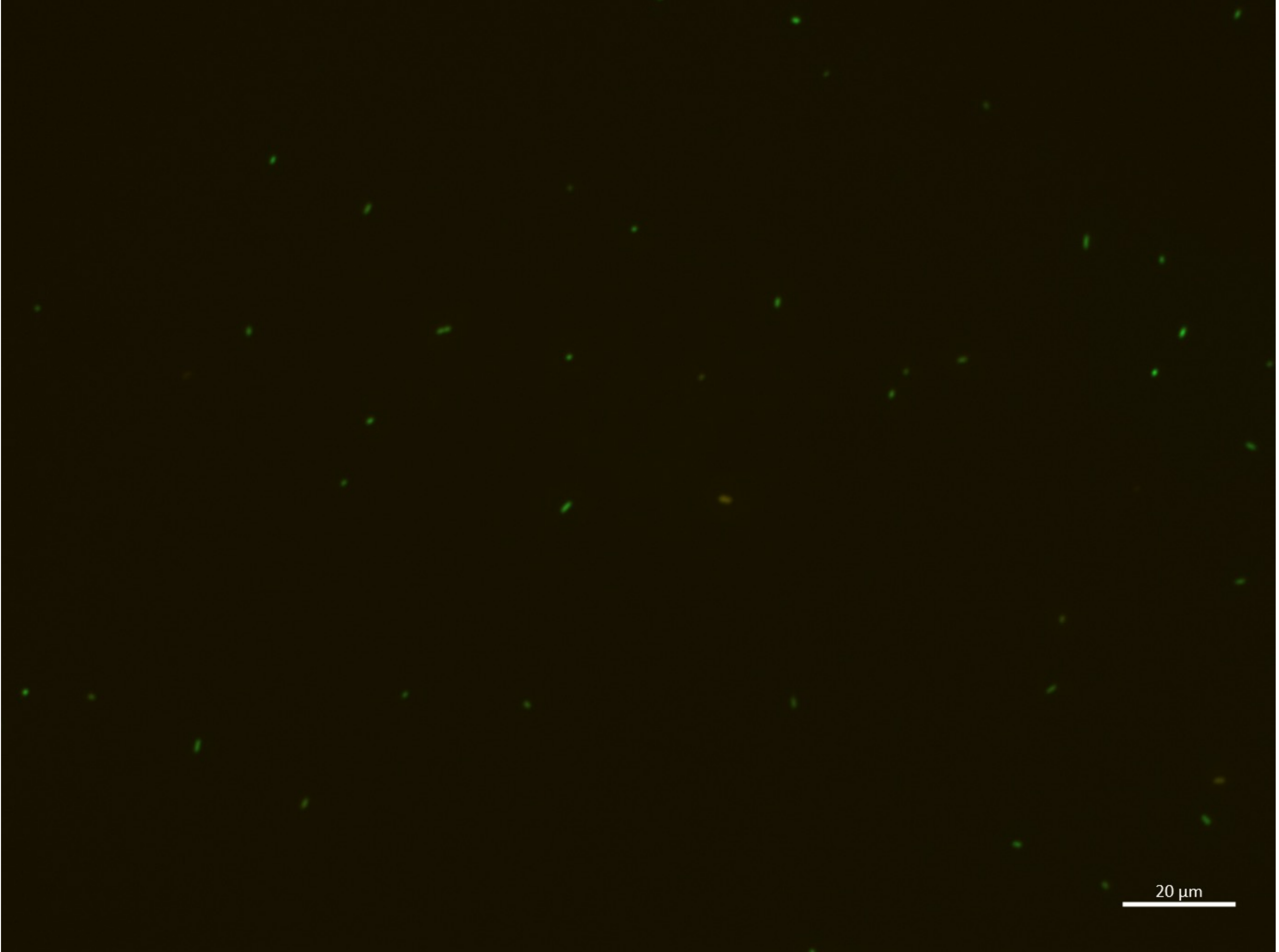

14:00-D

B.bifidum	**3**
E.coli	37
**Sum**	40

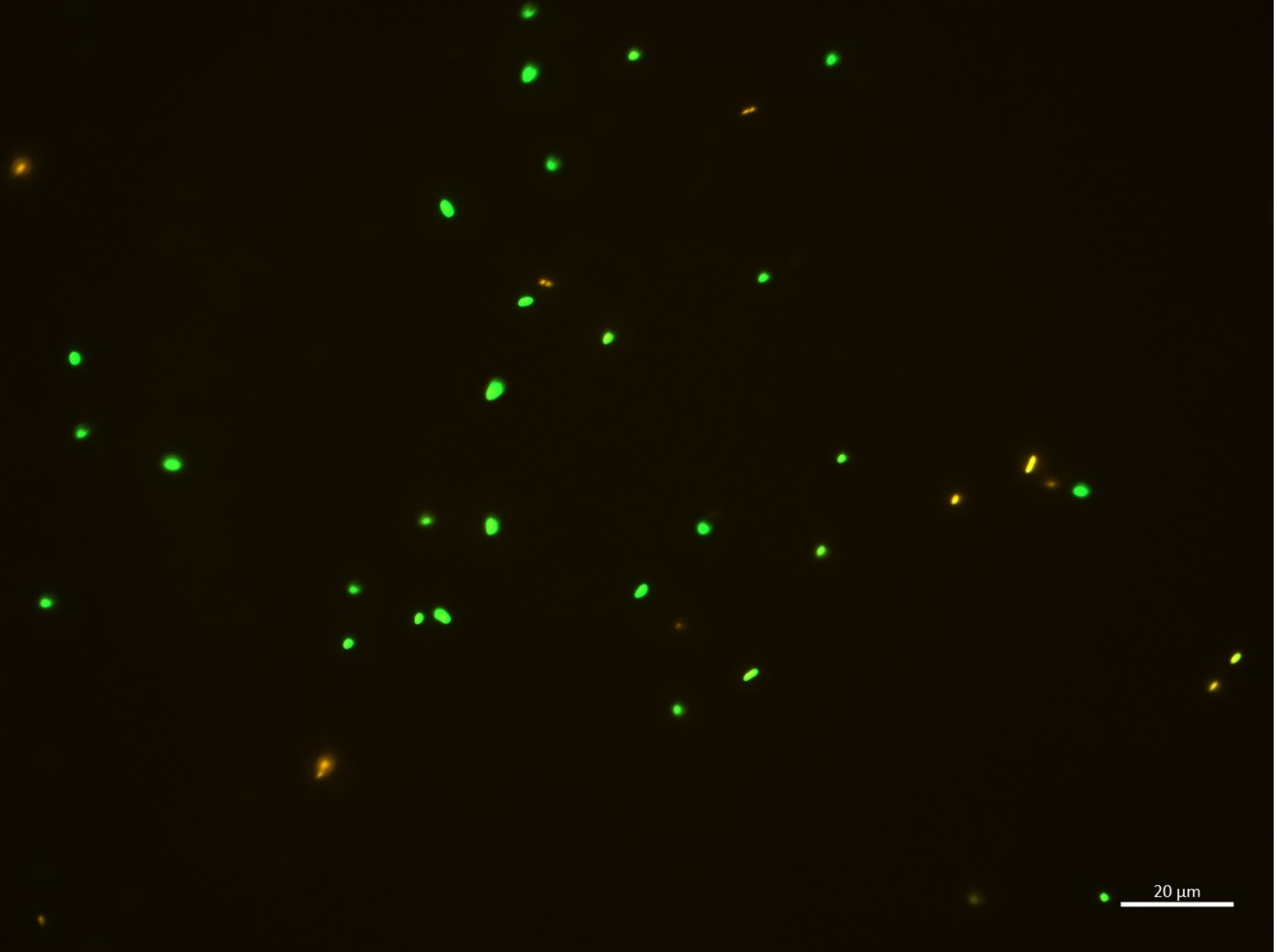

14:00-E

**B.bifidum**	**11**
**E.coli**	28
**Sum**	39

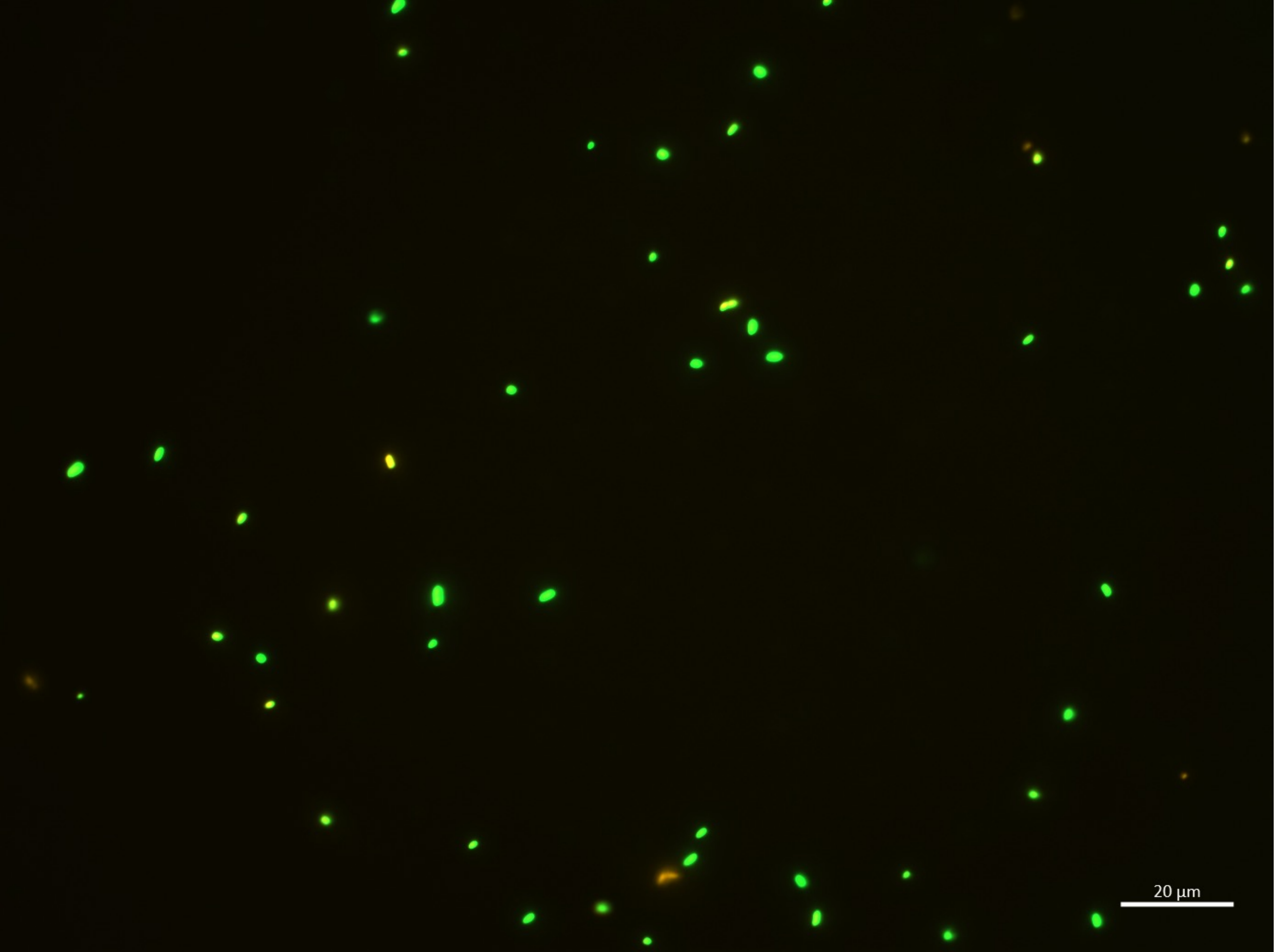

14:00-F

B.bifidum	**6**
E.coli	48
**Sum**	54

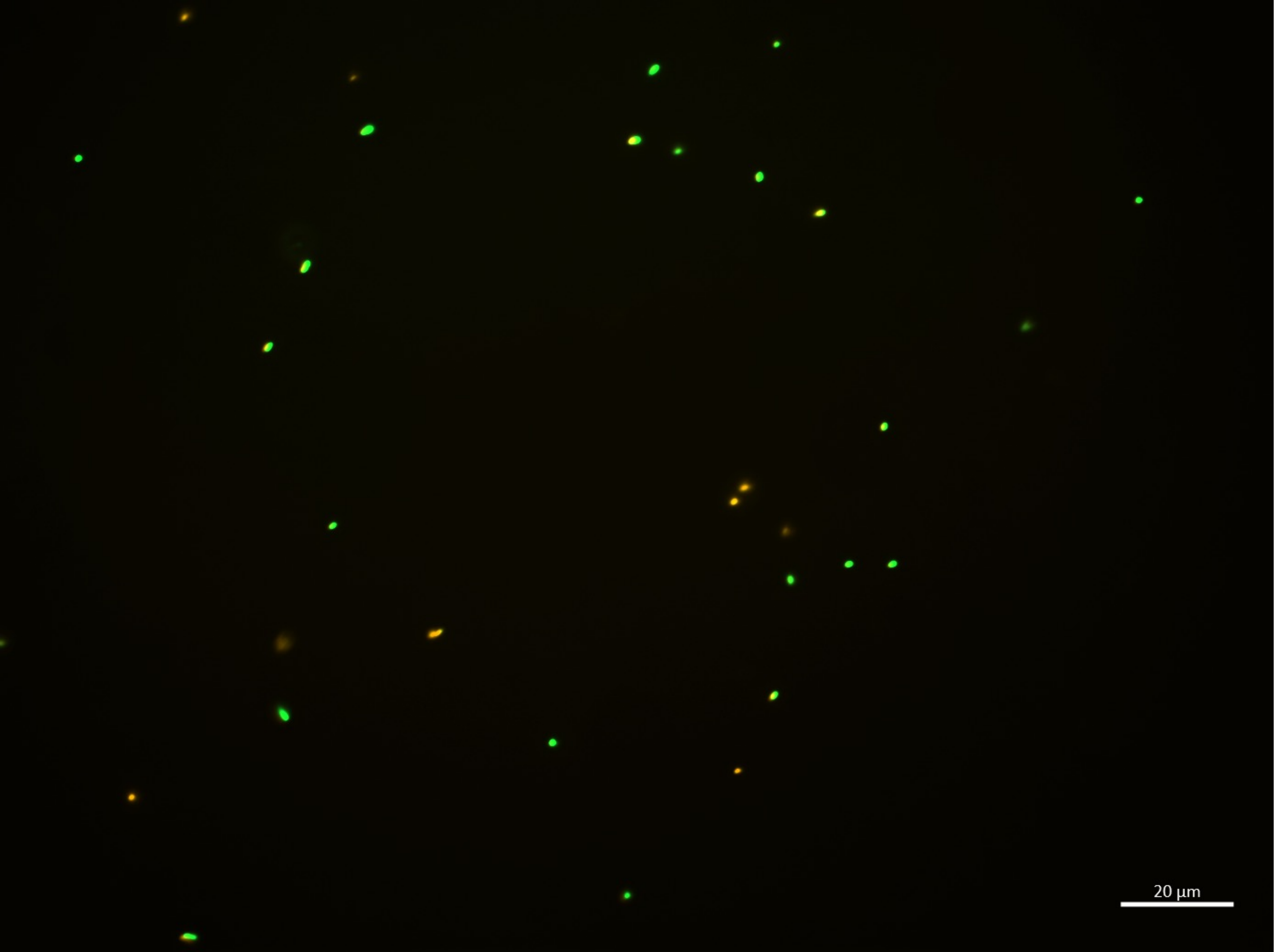

14:00-G

B.bifidum	**9**
E.coli	22
**Sum**	31

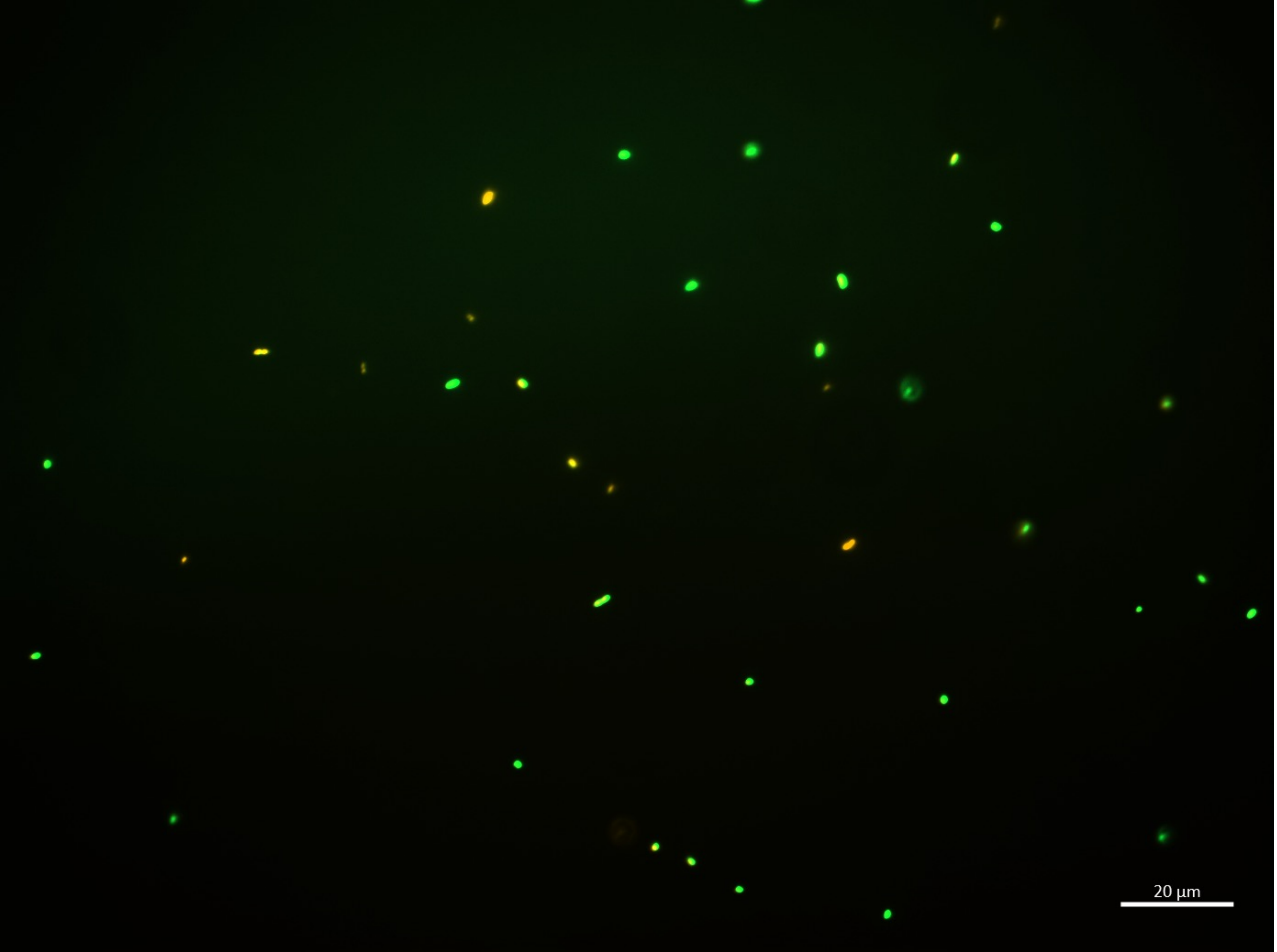

14:00-H

**B.bifidum**	**8**
**E.coli**	29
**Sum**	37

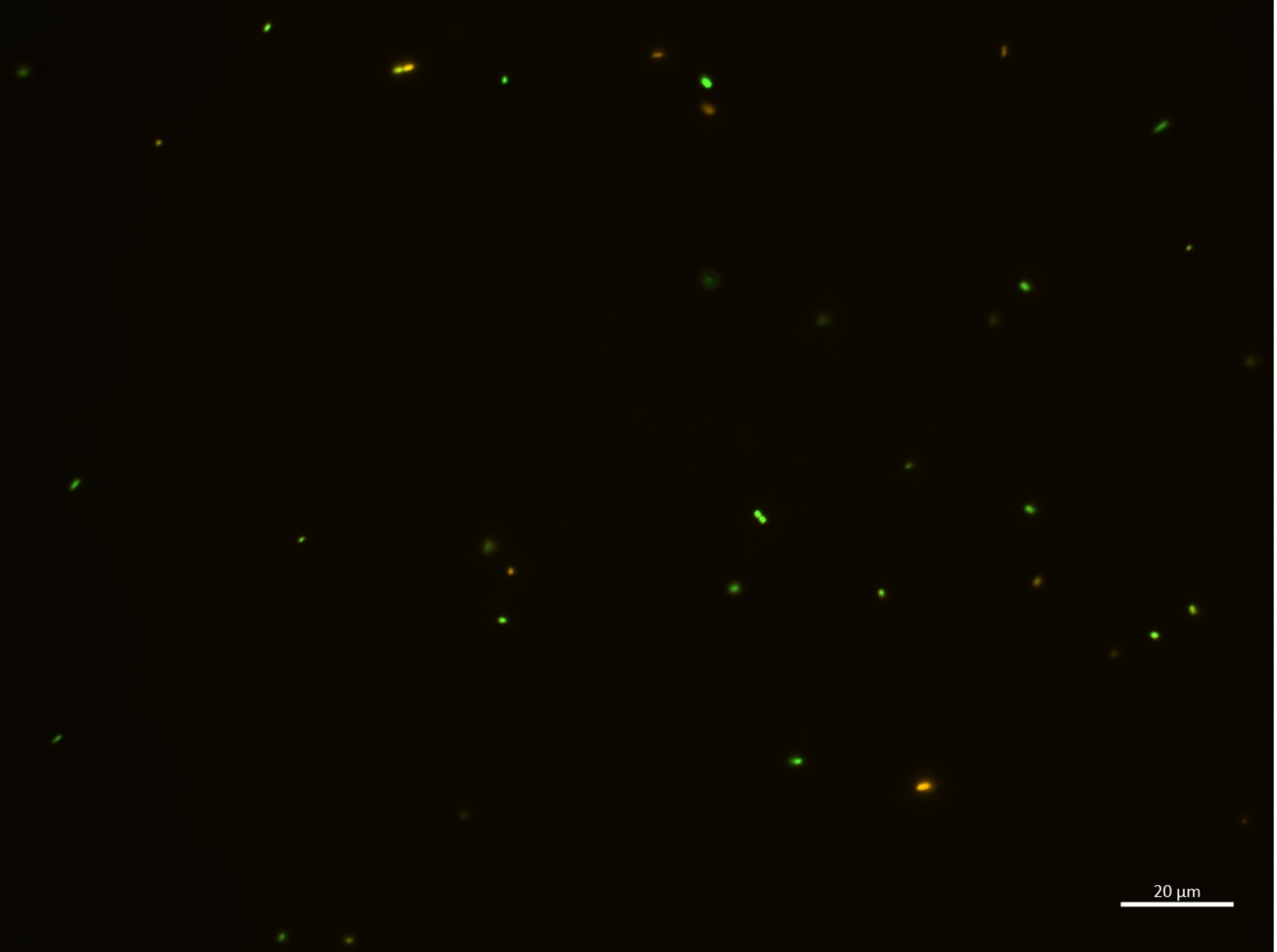

14:00-I

B.bifidum	**7**
E.coli	26
**Sum**	33

14:00-J

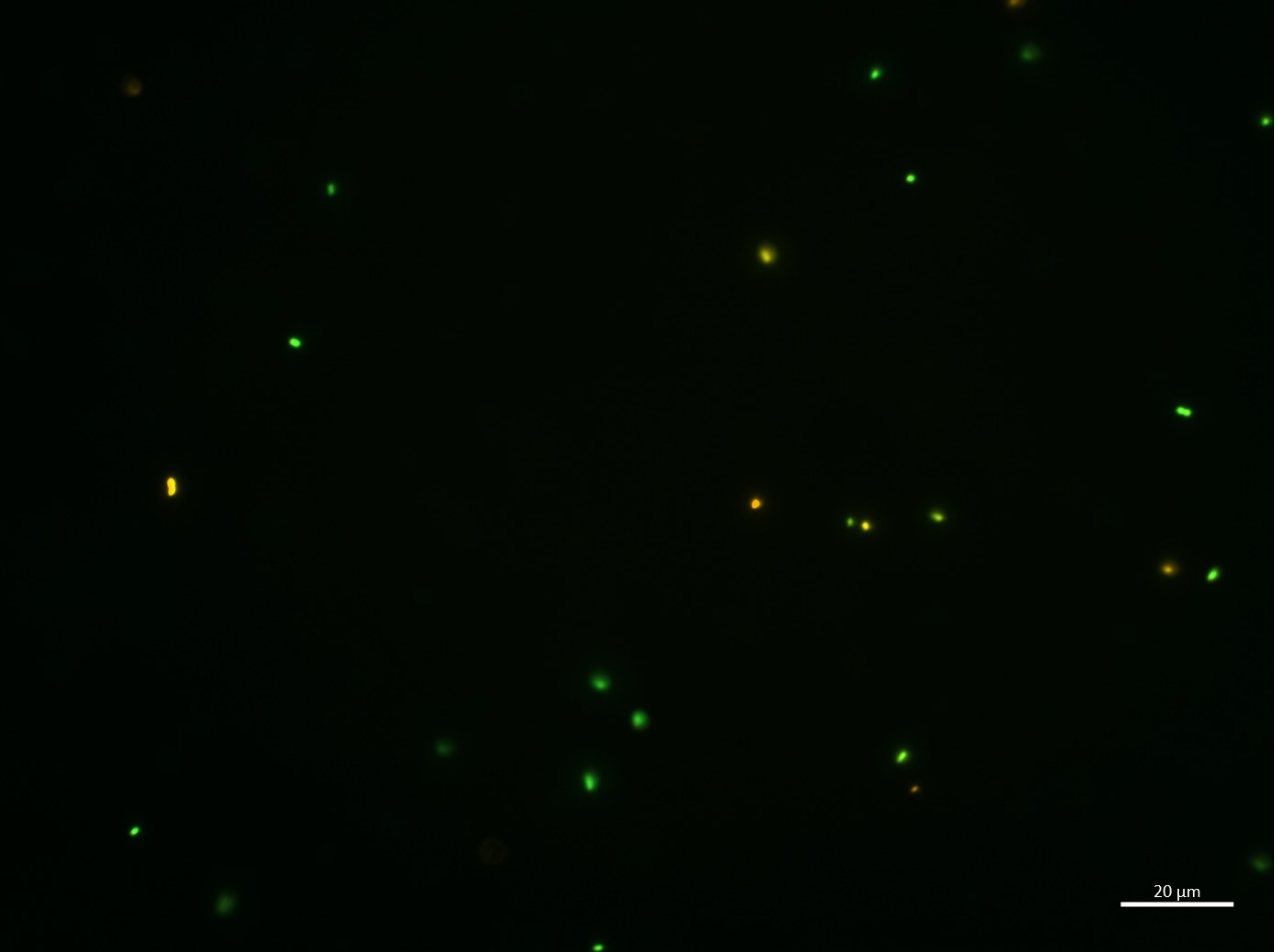

B.bifidum	**7**
E.coli	19
**Sum**	26

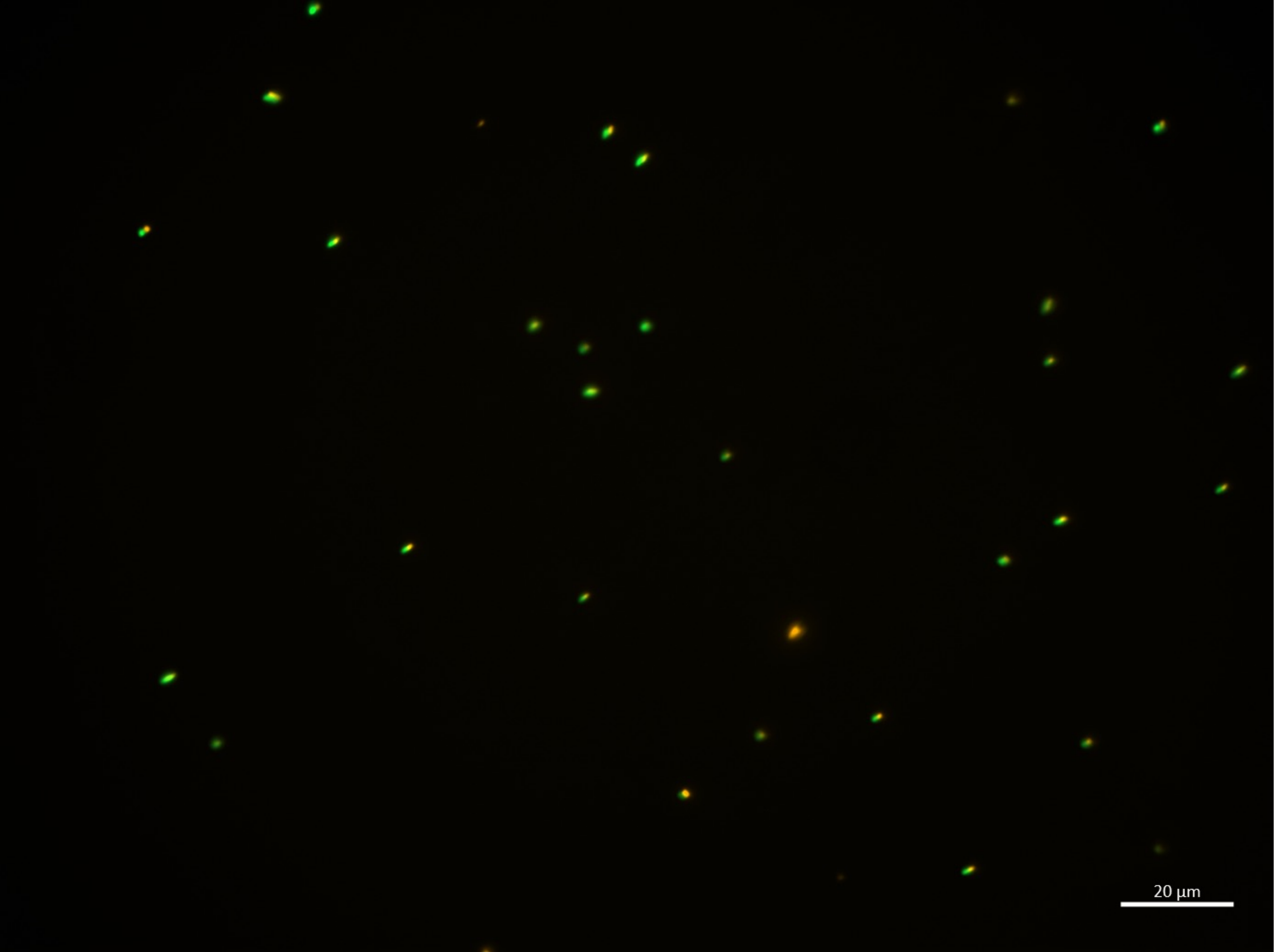

16:00-A

B.bifidum	**4**
E.coli	25
**Sum**	29

16:00-B

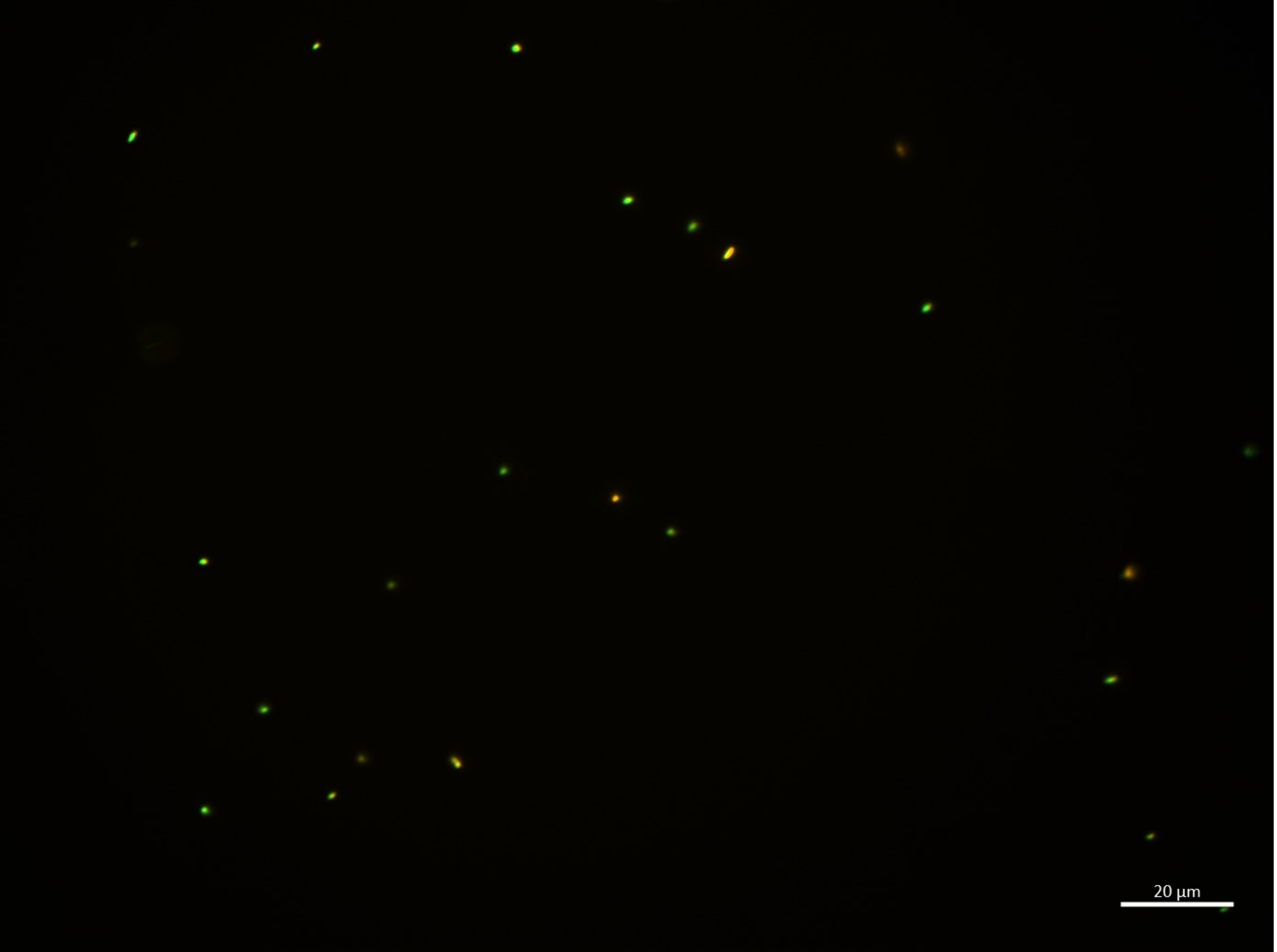

**B.bifidum**	**4**
**E.coli**	18
**Sum**	22

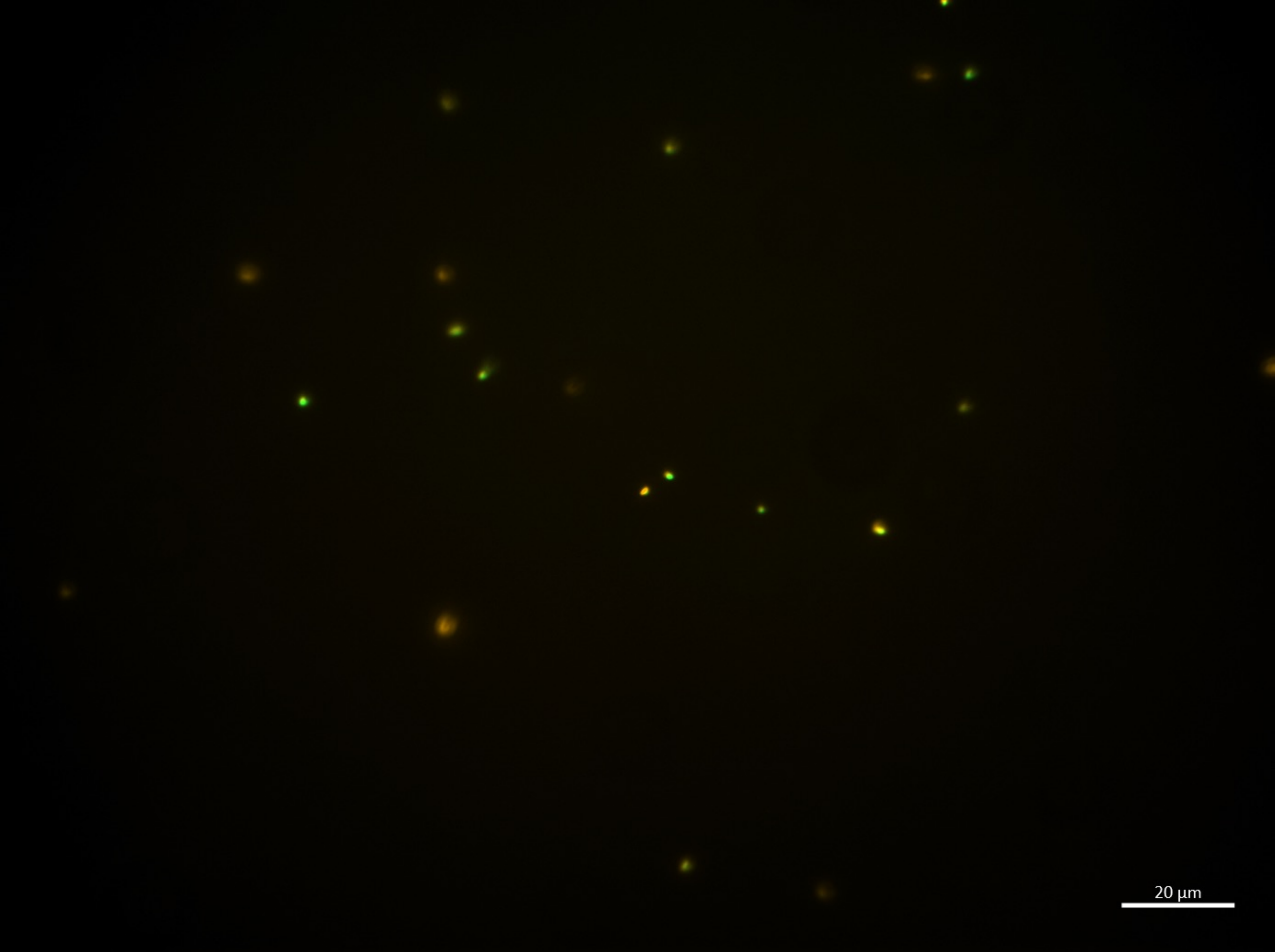

16:00-C

B.bifidum	**11**
E.coli	9
**Sum**	20

16:00-D

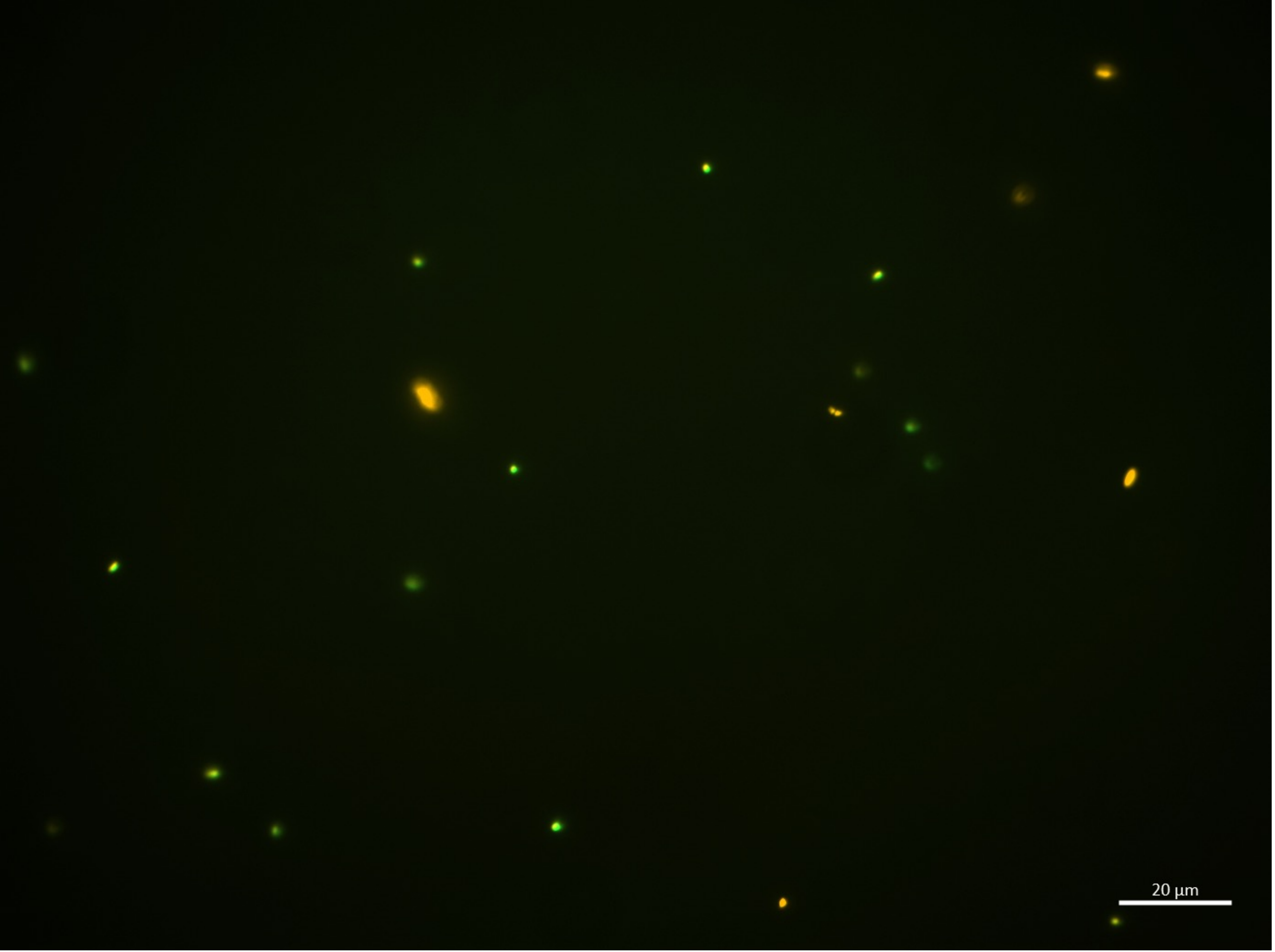

**B.bifidum**	**6**
**E.coli**	14
**Sum**	20

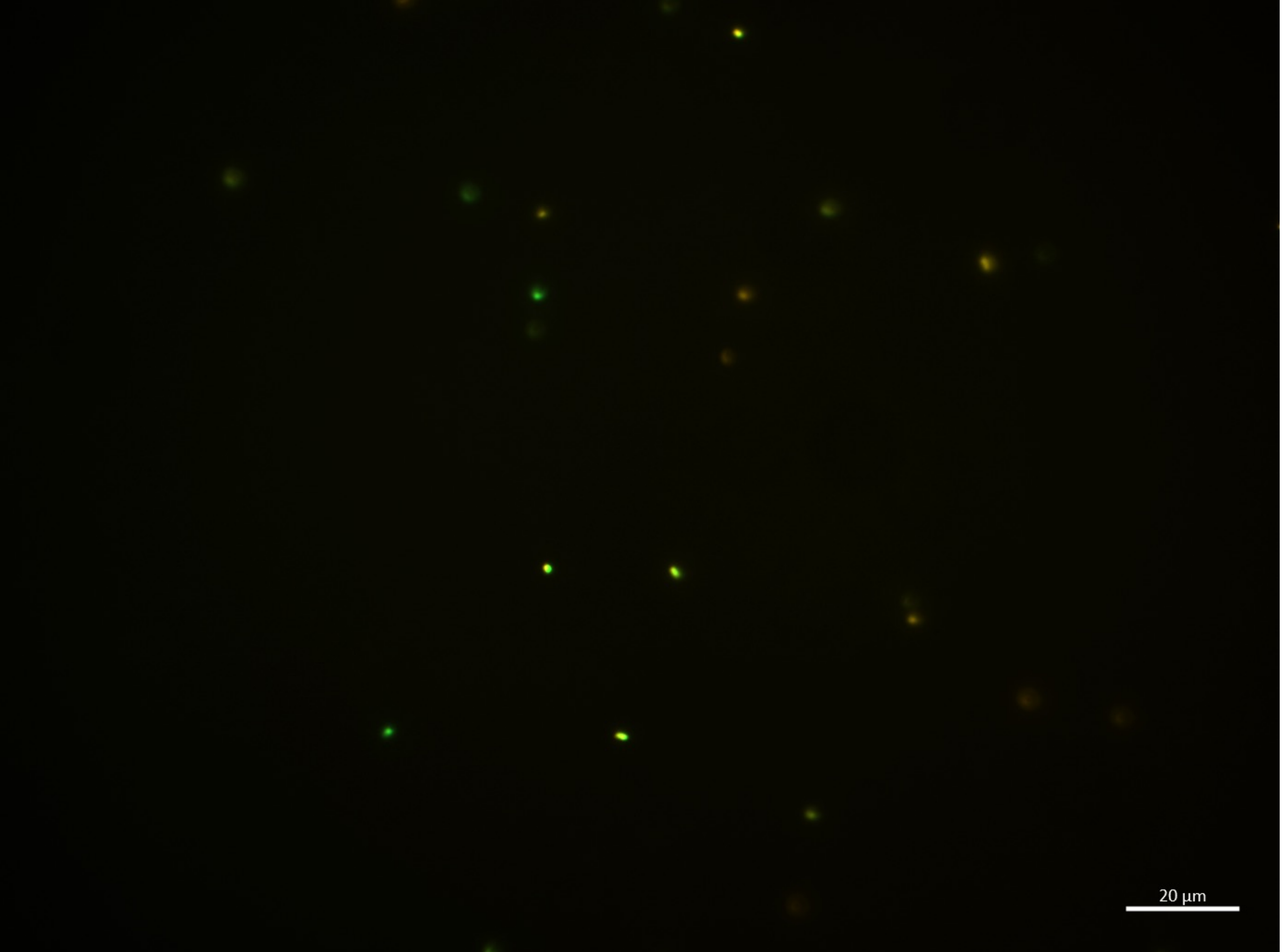

16:00-E

**B.bifidum**	**6**
**E.coli**	12
**Sum**	18

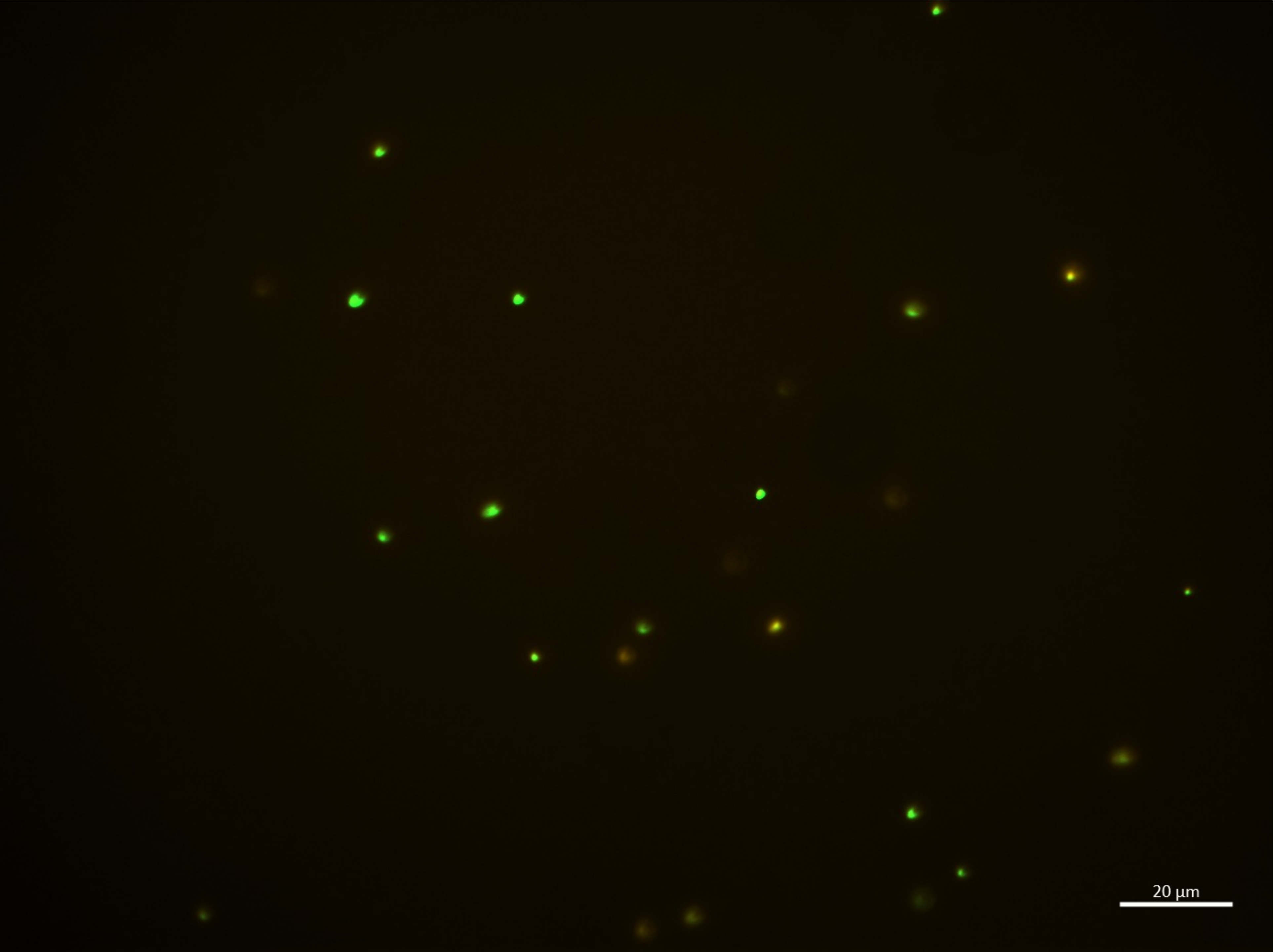

16:00-F

B.bifidum	**7**
E.coli	17
**Sum**	24

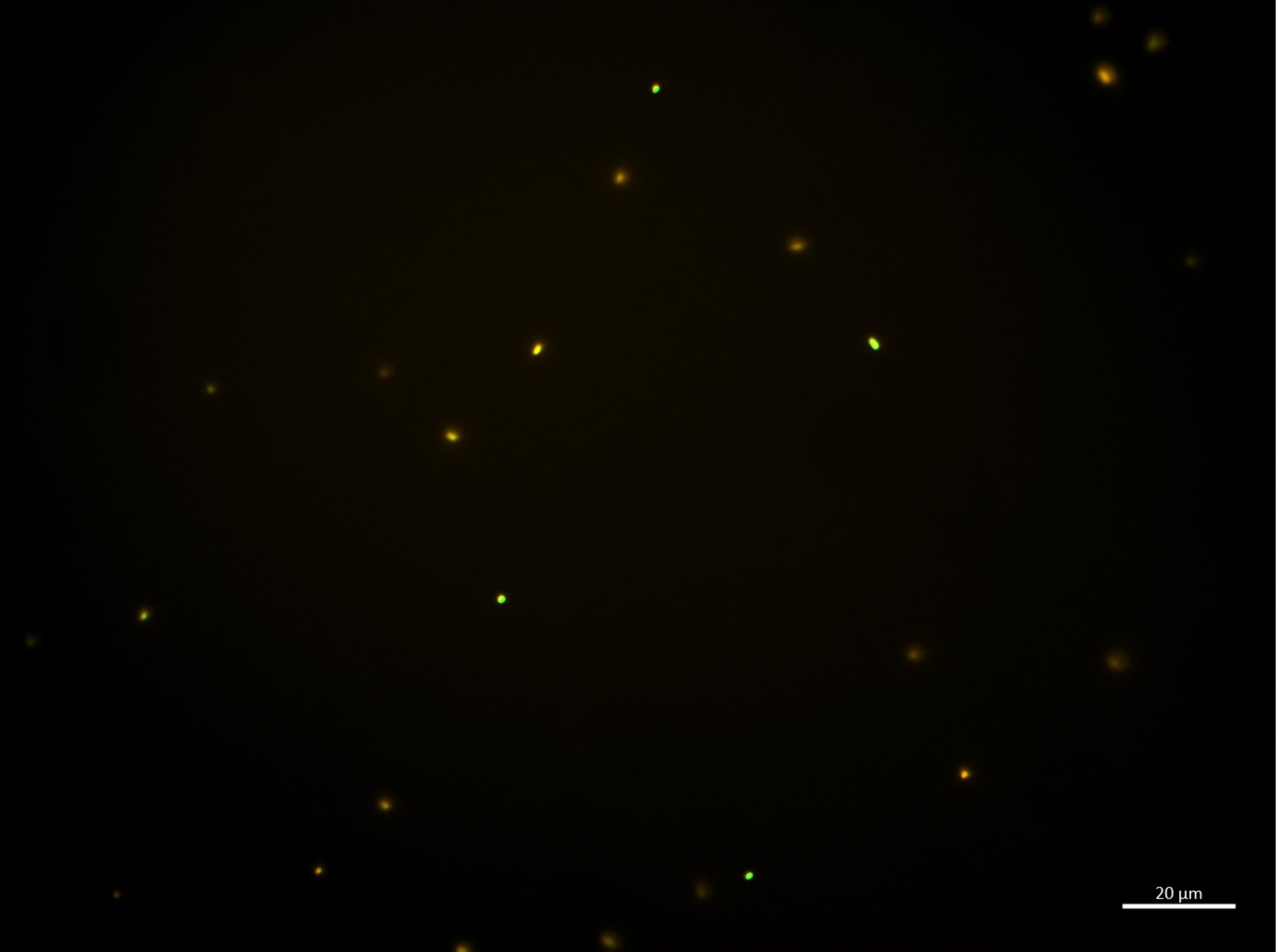

16:00-G

**B.bifidum**	**15**
**E.coli**	8
**Sum**	23

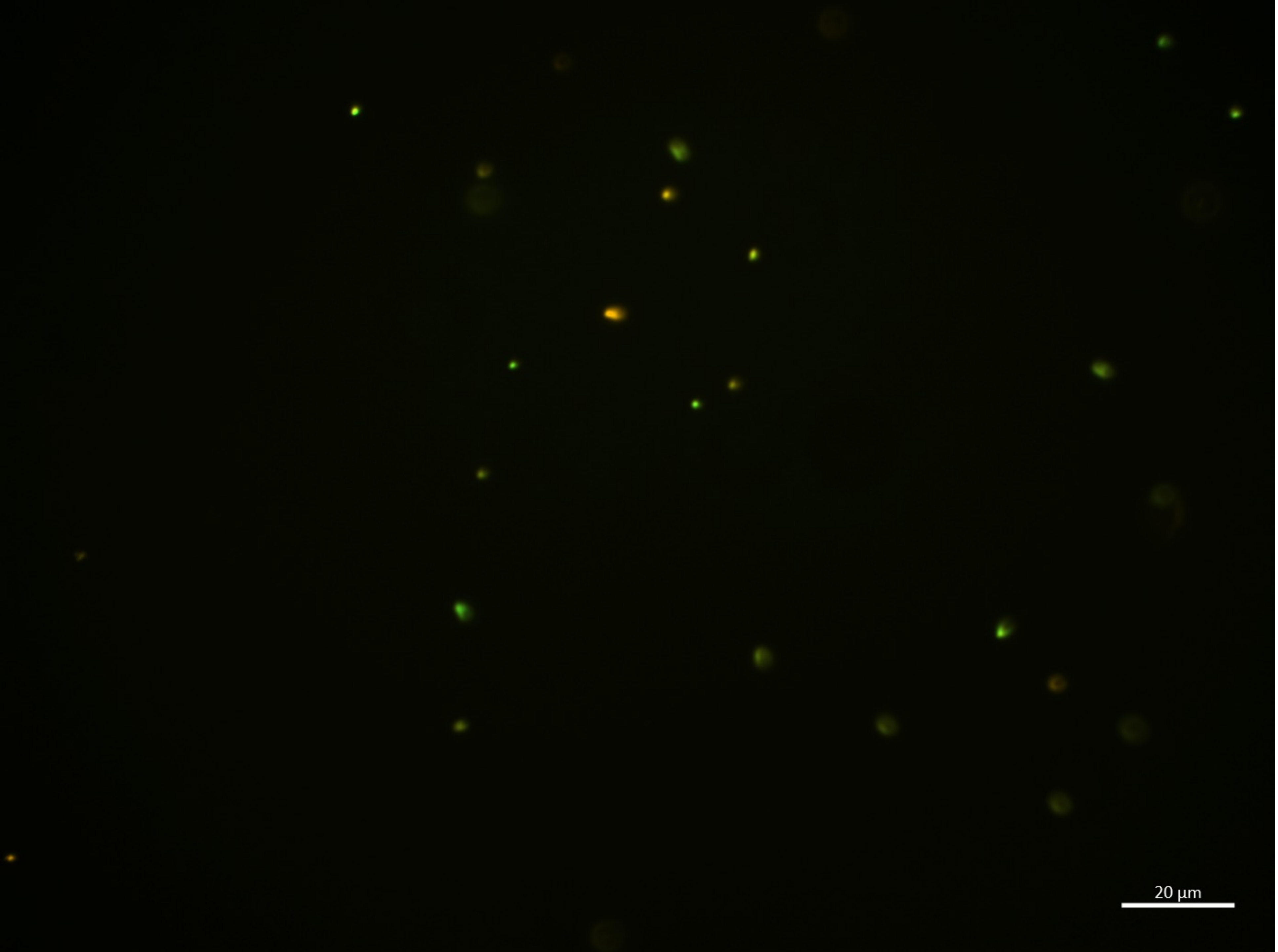

16:00-H

**B.bifidum**	**4**
**E.coli**	17
**Sum**	21

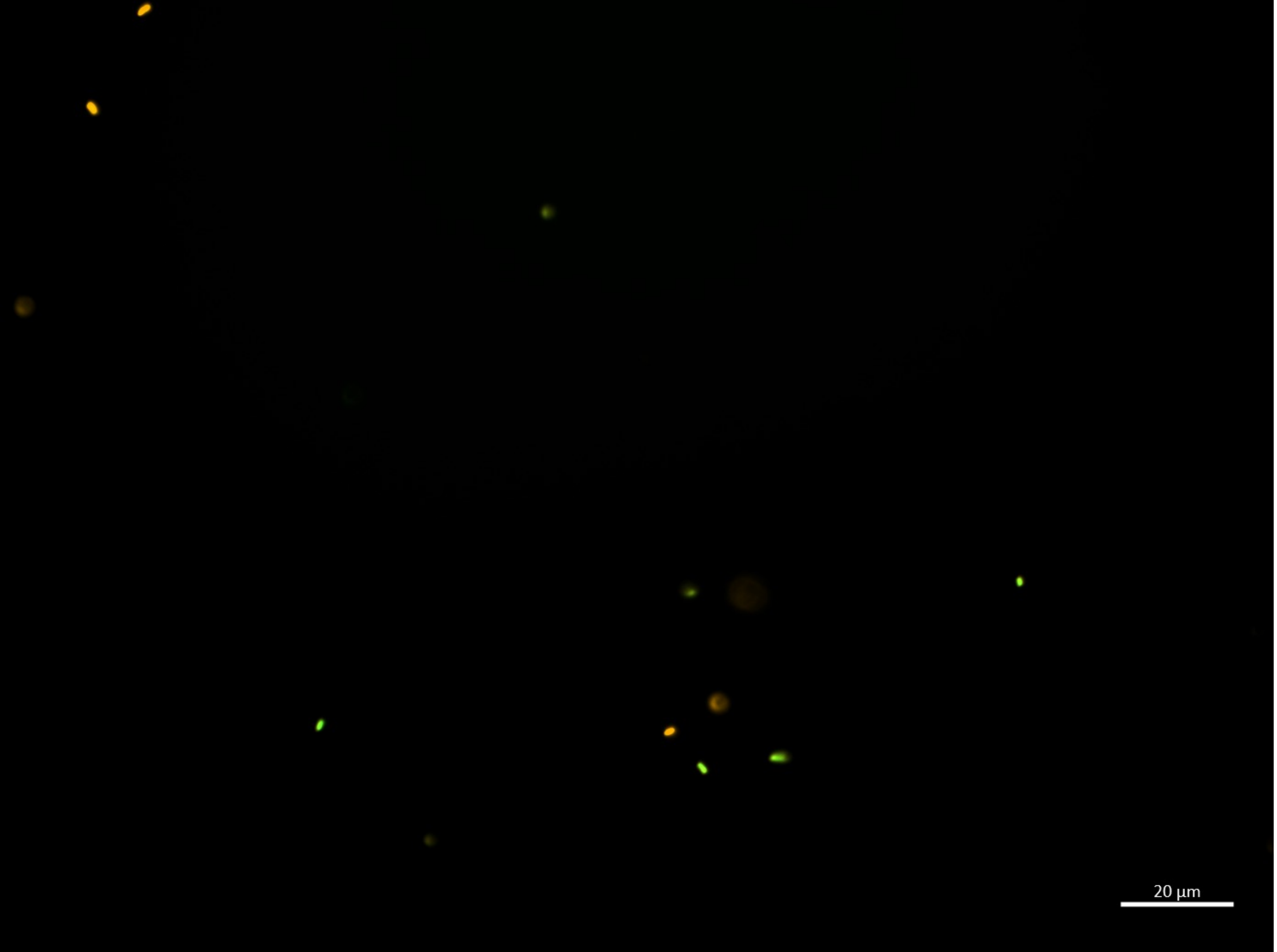

16:00-I

B.bifidum	4
E.coli	7
Sum	11

16:00-J

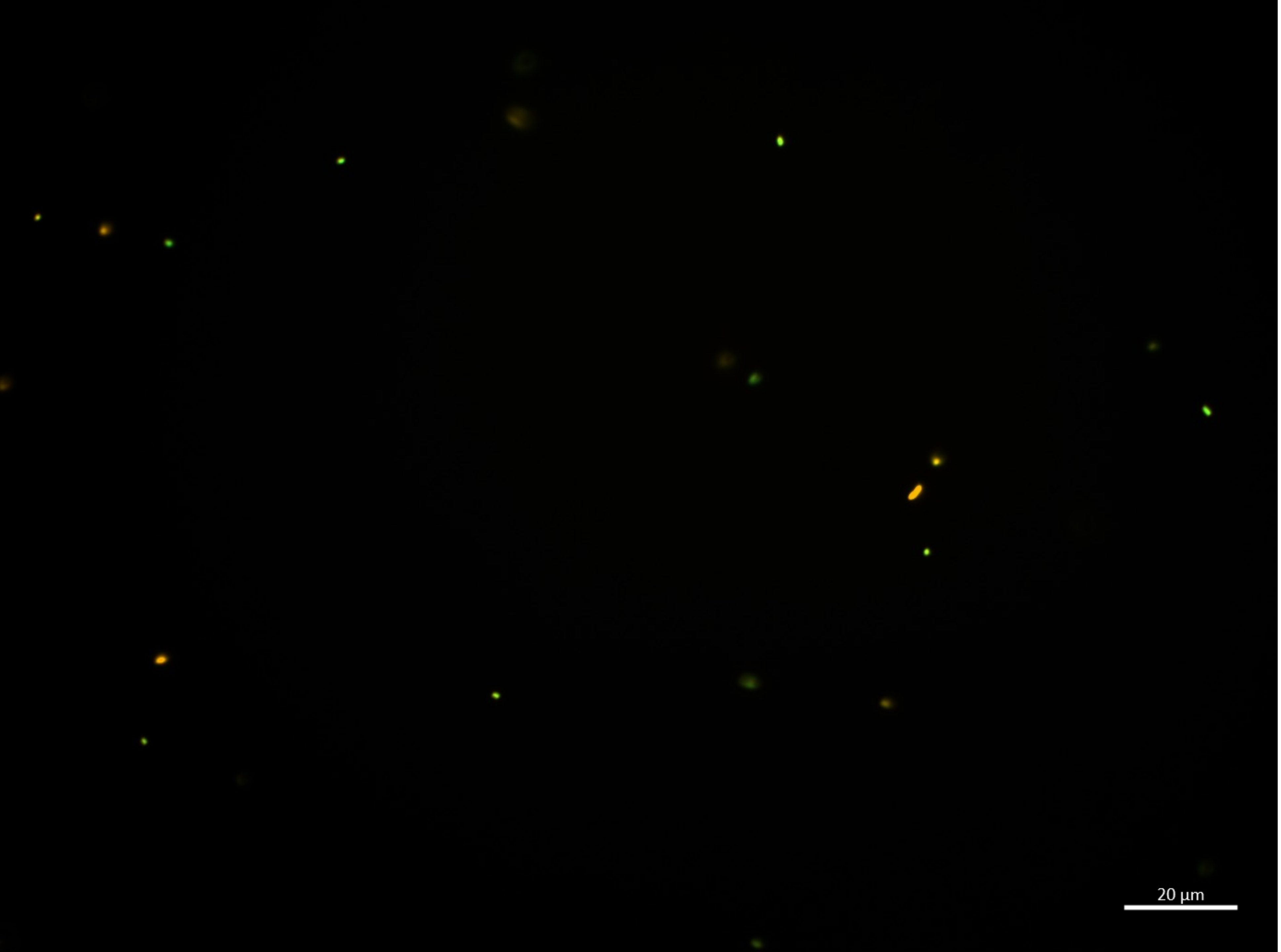

B.bifidum	6
E.coli	11
Sum	17

18:00-A

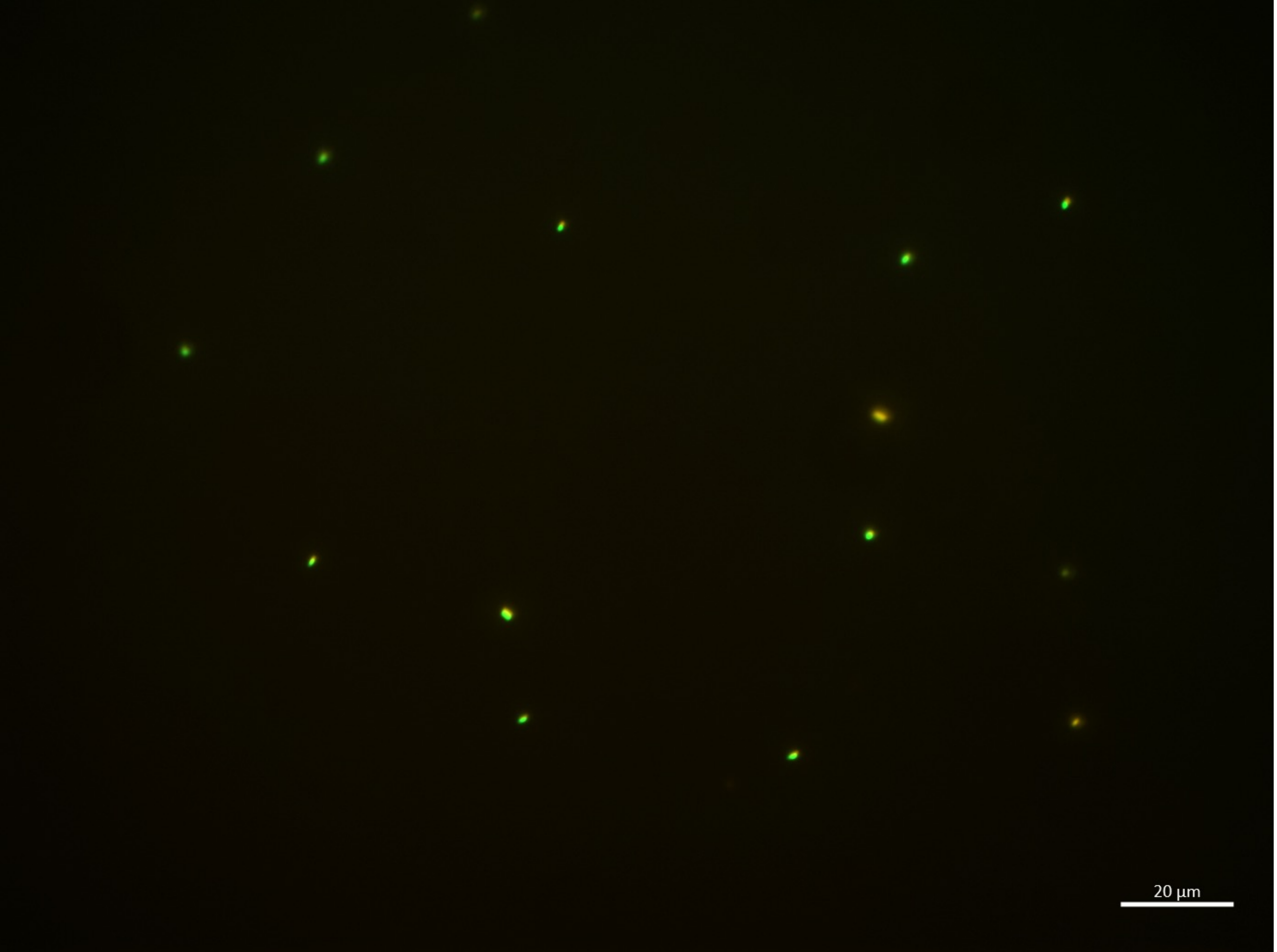

**B.bifidum**	**2**
**E.coli**	11
**Sum**	13

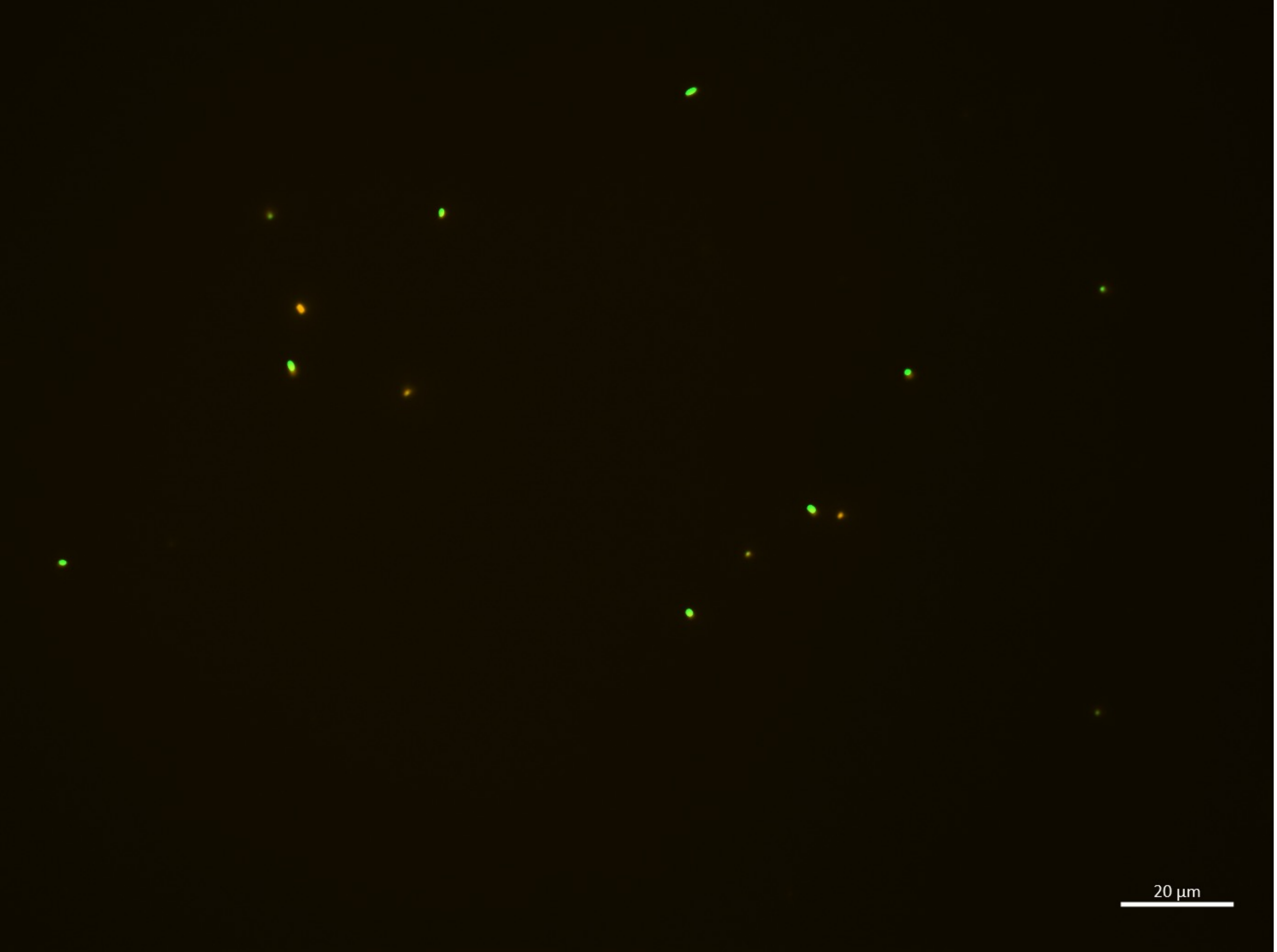

18:00-B

**B.bifidum**	**3**
**E.coli**	11
**Sum**	14

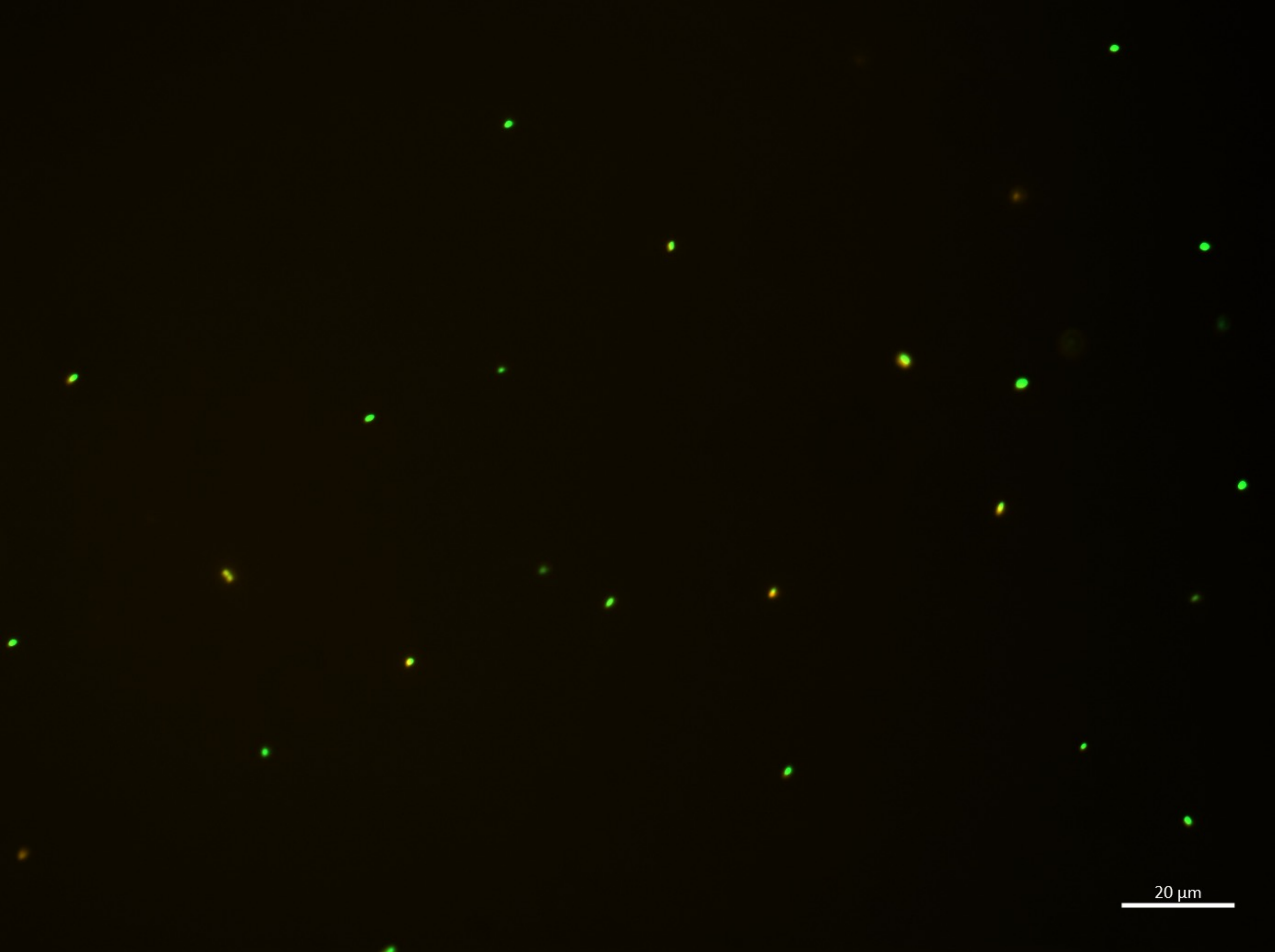

18:00-C

B.bifidum	**2**
E.coli	23
**Sum**	25

18:00-D

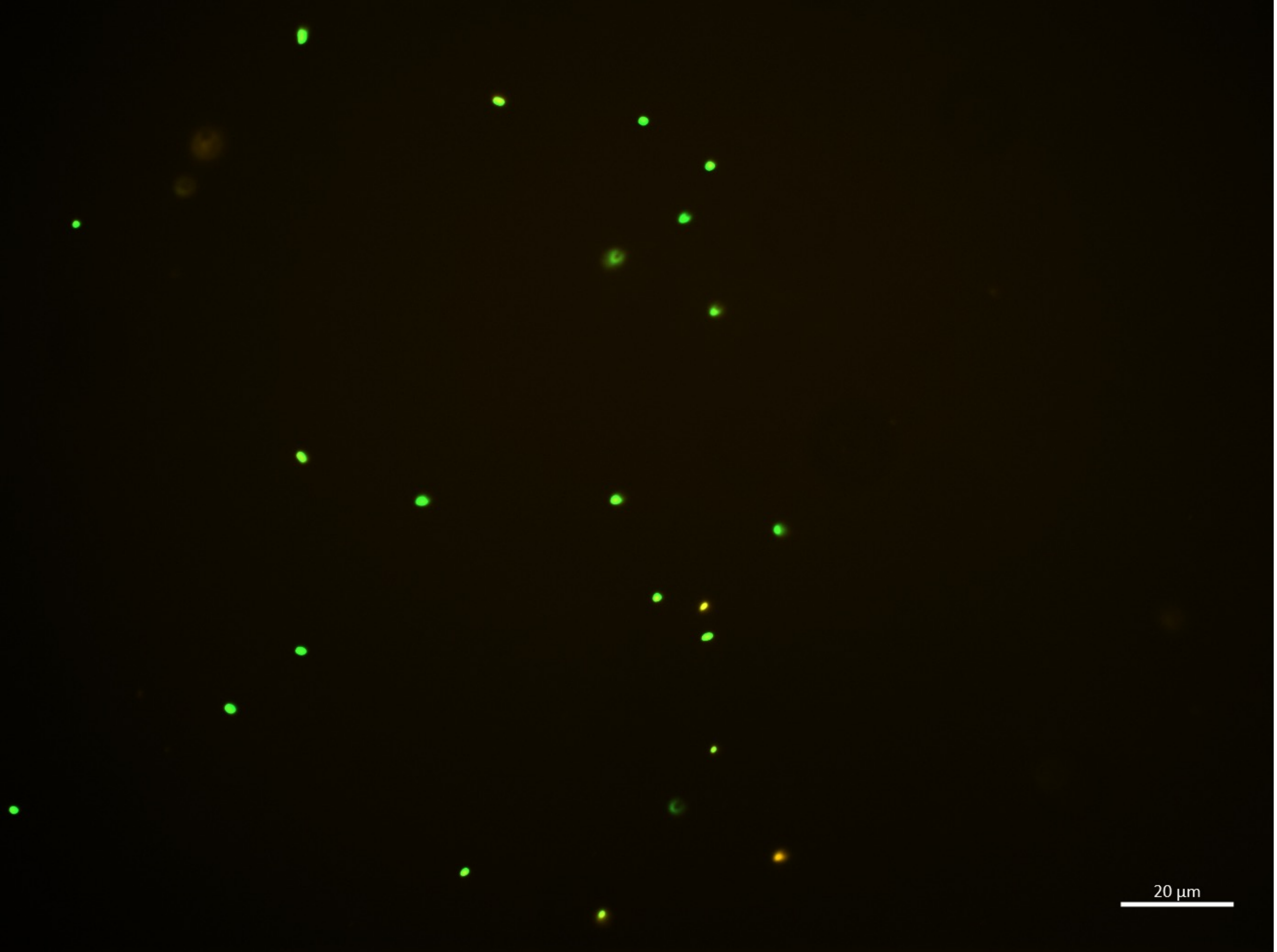

B.bifidum	**3**
E.coli	20
**Sum**	23

18:00-E

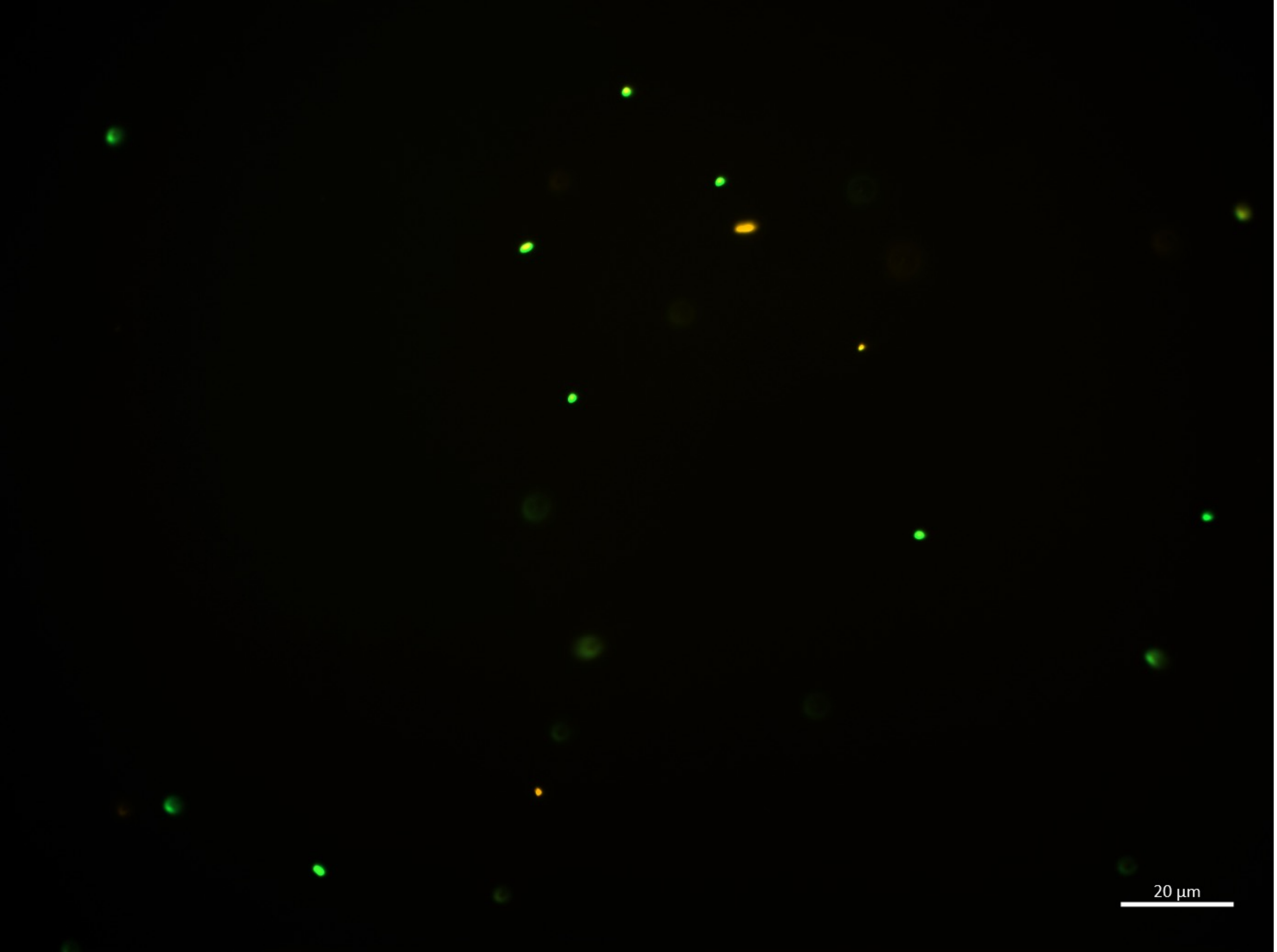

B.bifidum	**2**
E.coli	12
**Sum**	14

18:00-F

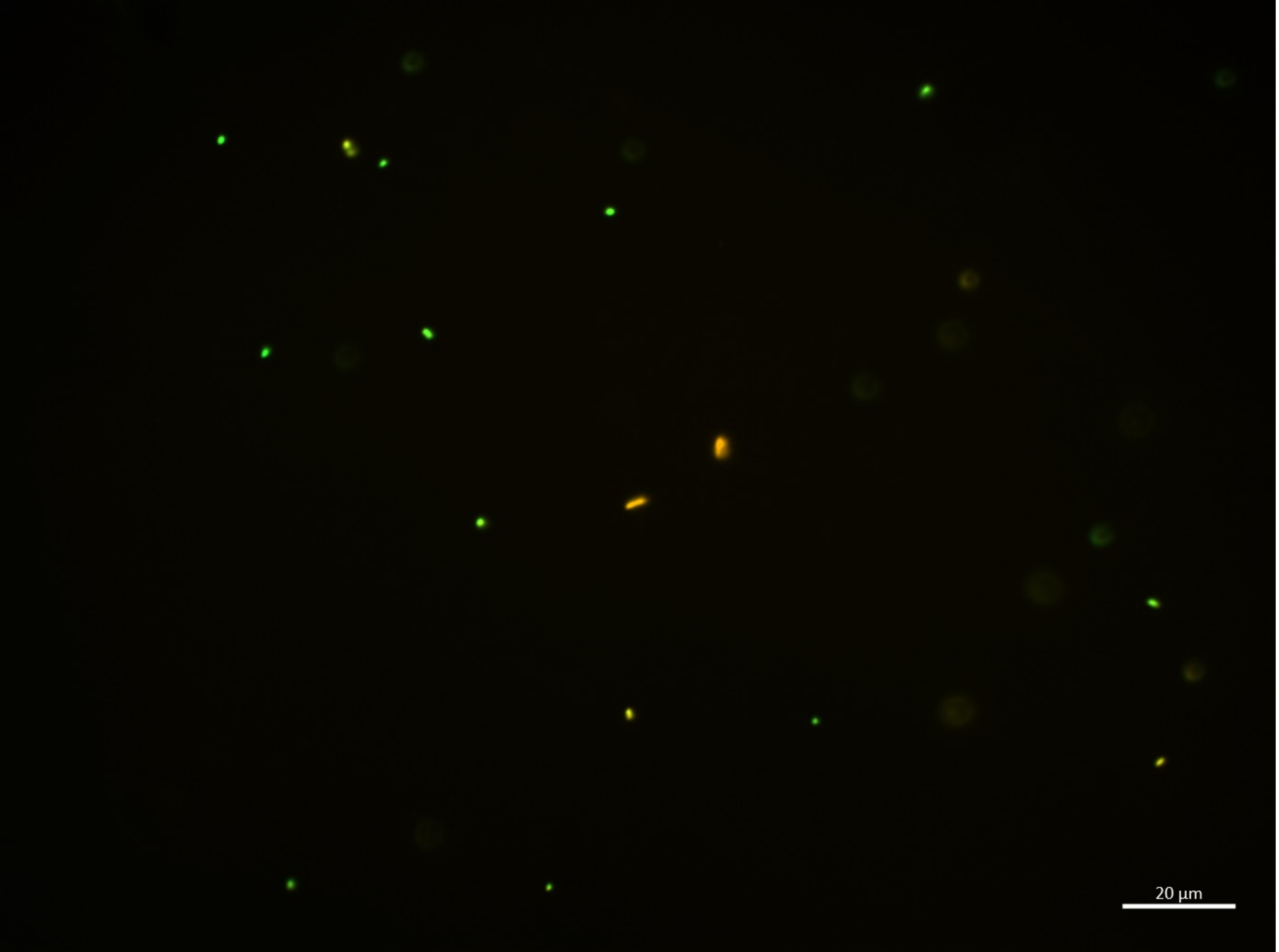

B.bifidum	**3**
E.coli	12
**Sum**	15

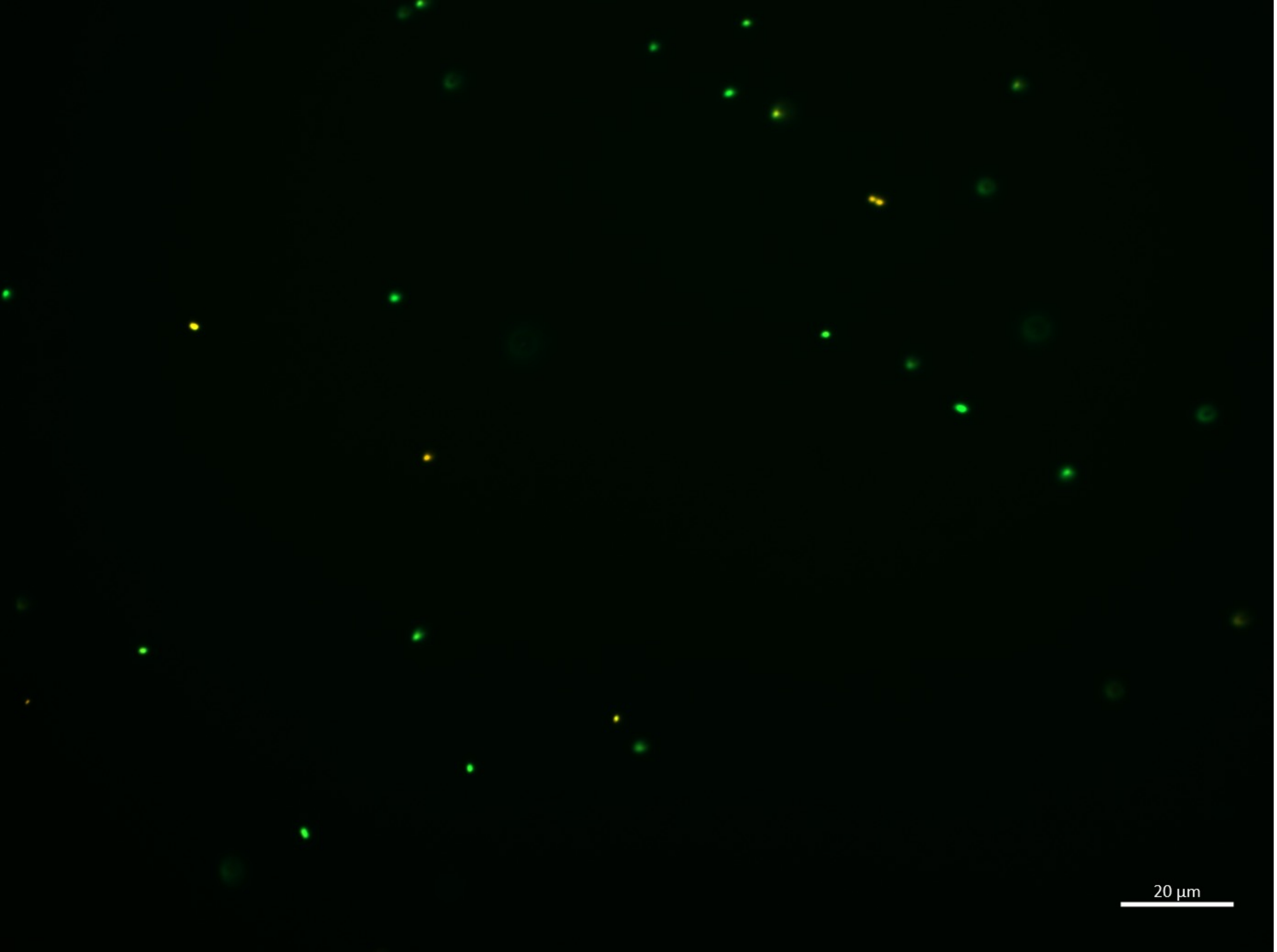

18:00-G

B.bifidum	**3**
E.coli	22
**Sum**	25

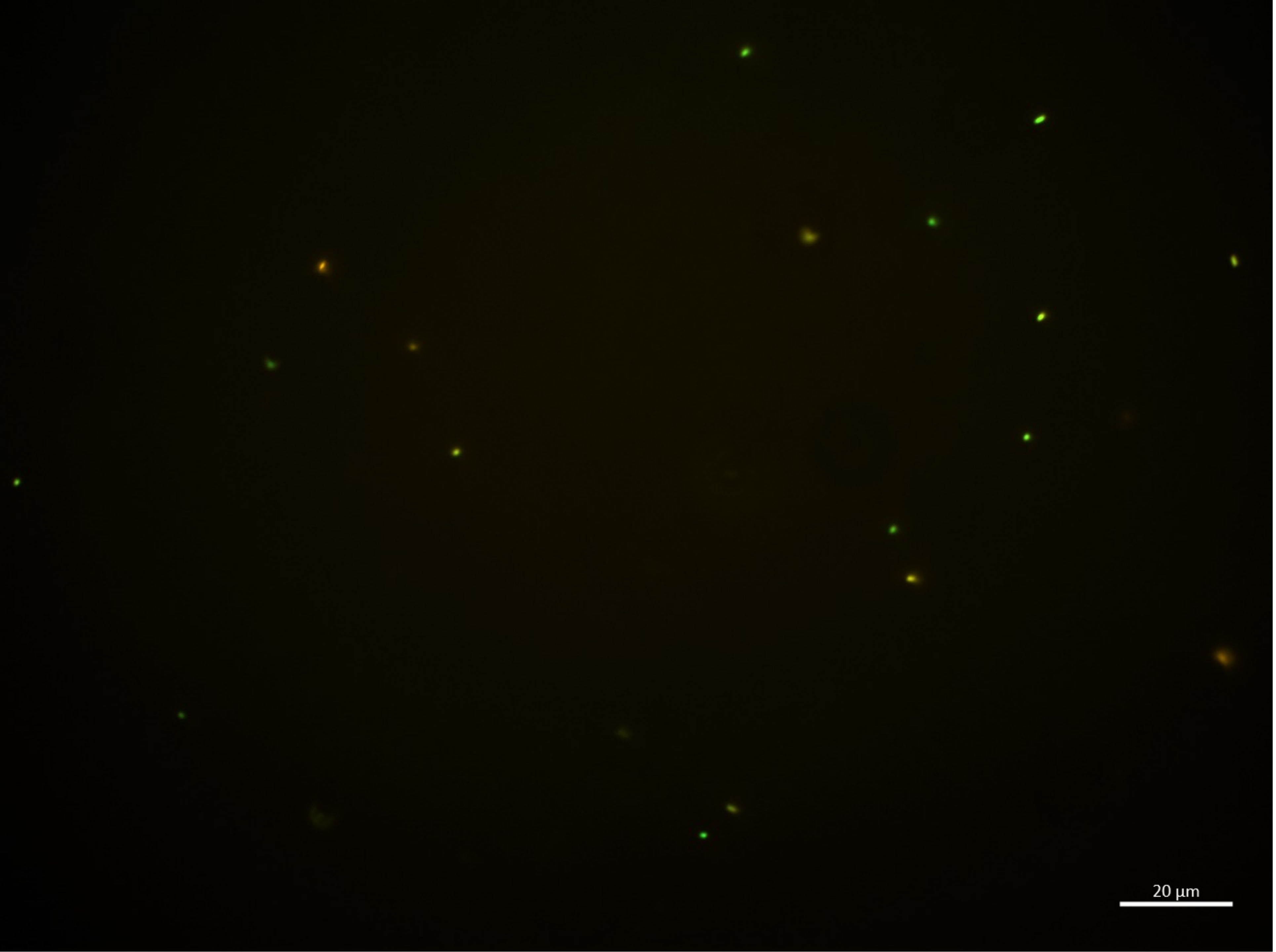

18:00-H

B.bifidum	**5**
E.coli	14
**Sum**	19

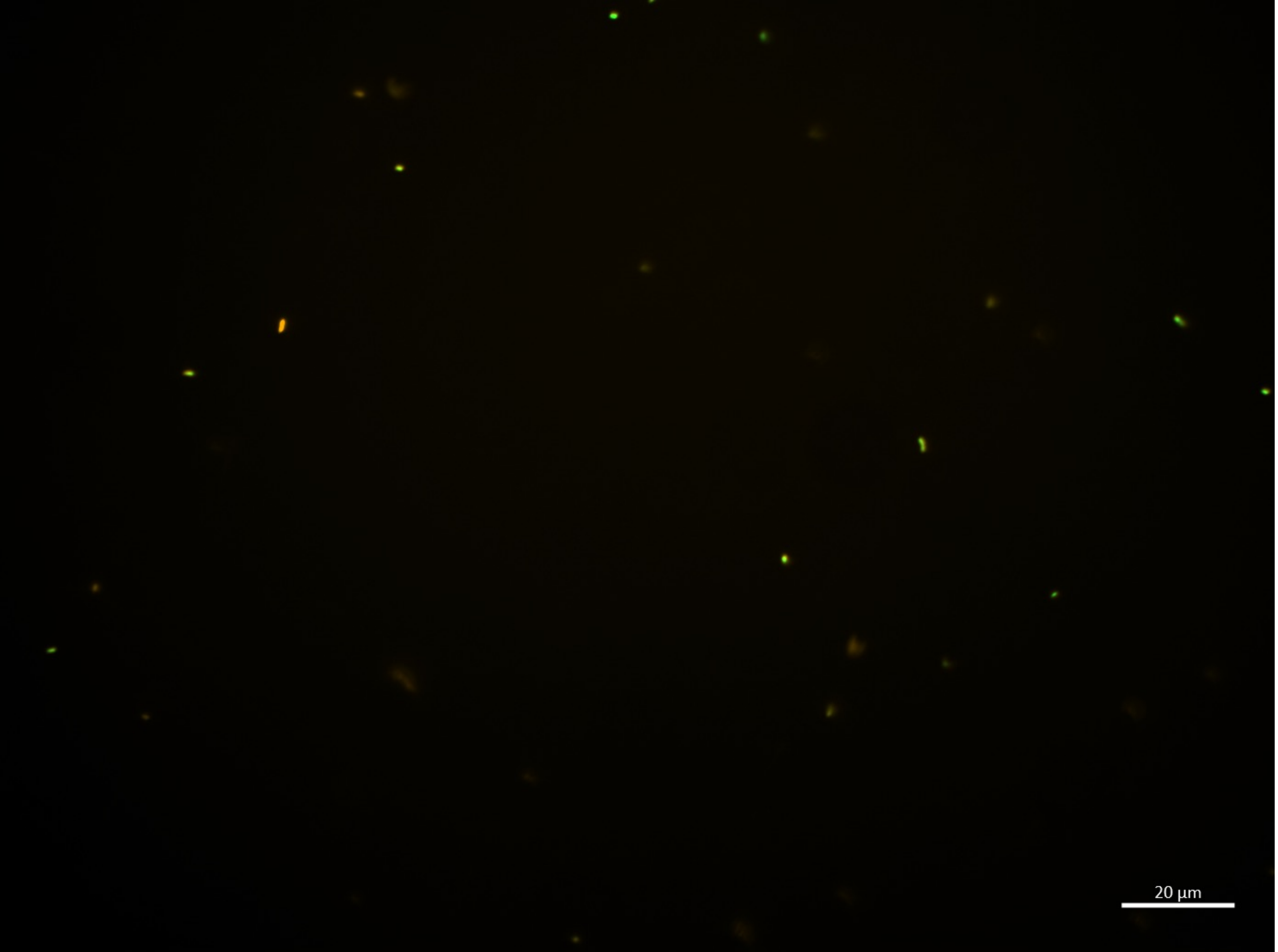

18:00-I

**B.bifidum**	**8**
**E.coli**	12
**Sum**	20

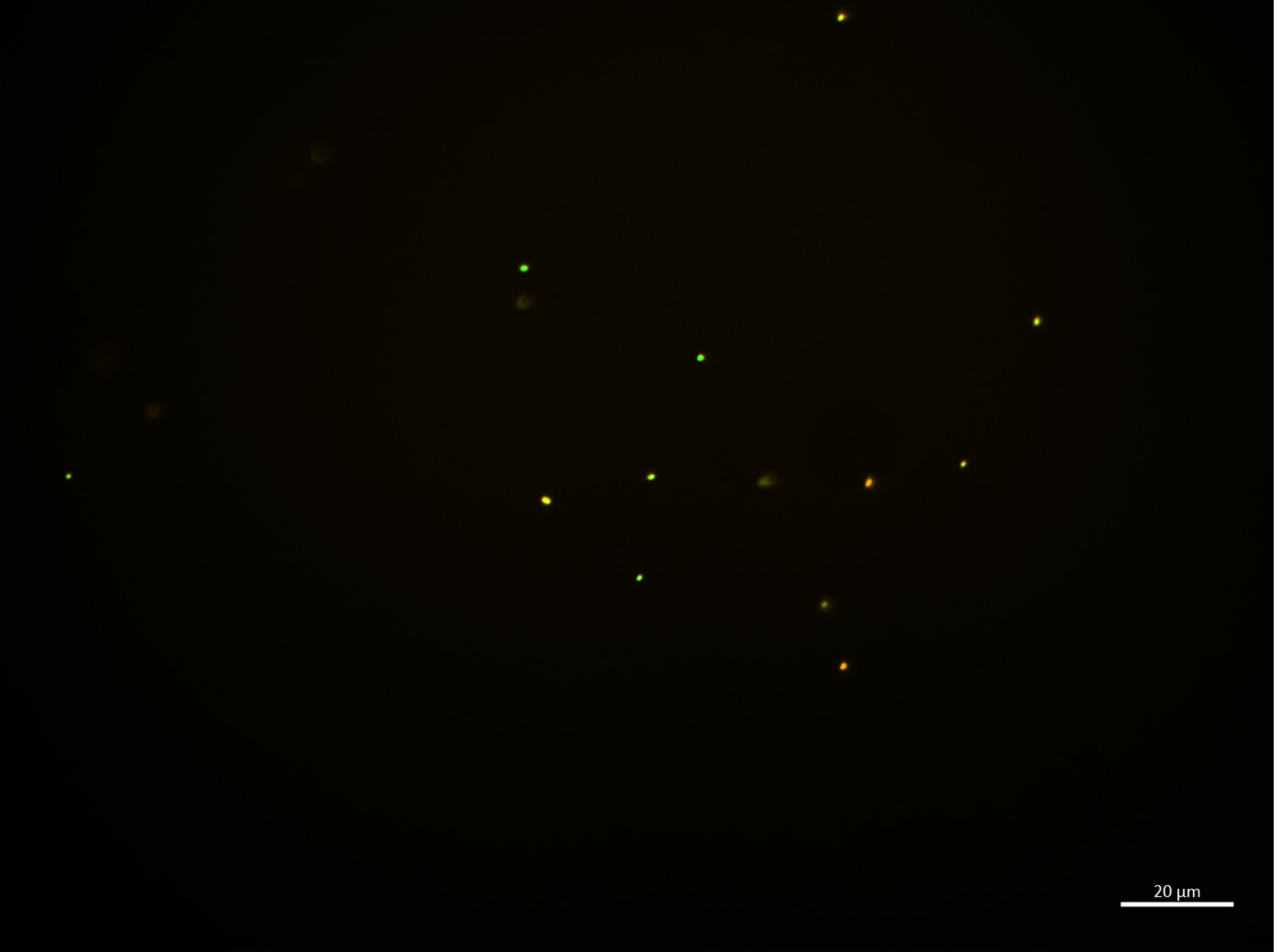

18:00-J

B.bifidum	5
E.coli	9
Sum	14

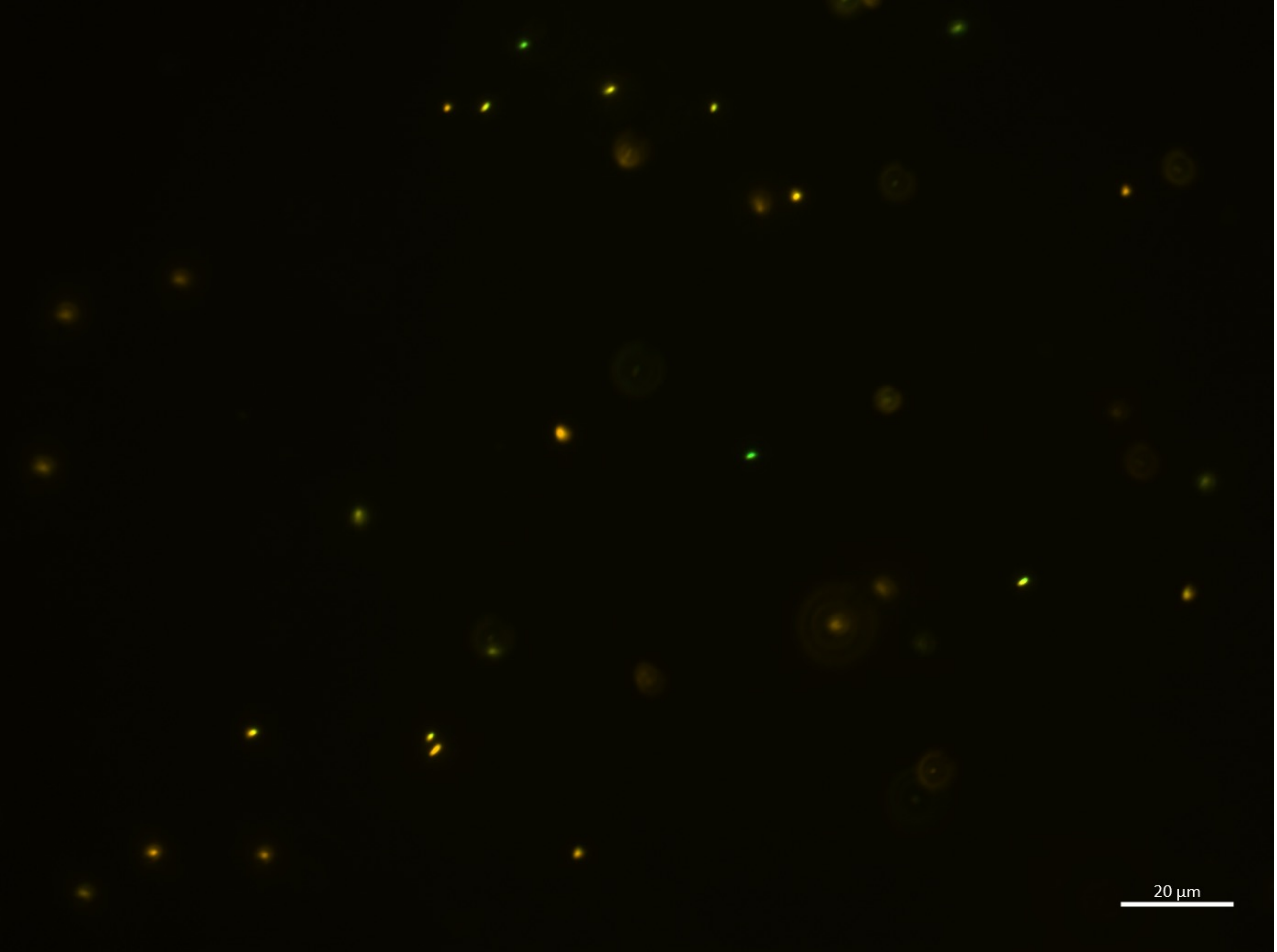

20:00-A

**B.bifidum**	**33**
**E.coli**	4
**Sum**	37

20:00-B

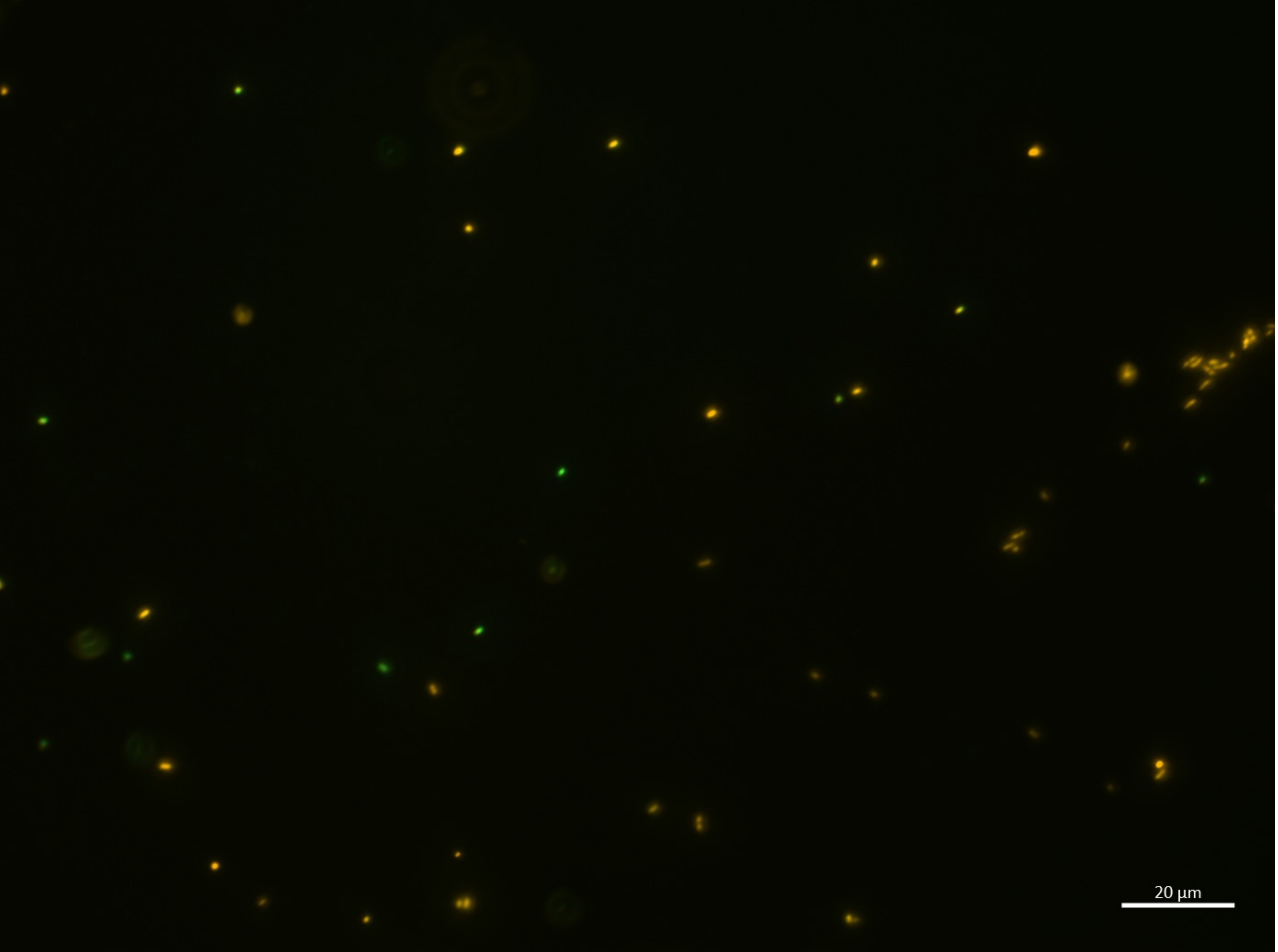

**B.bifidum**	**51**
E.coli	6
**Sum**	57

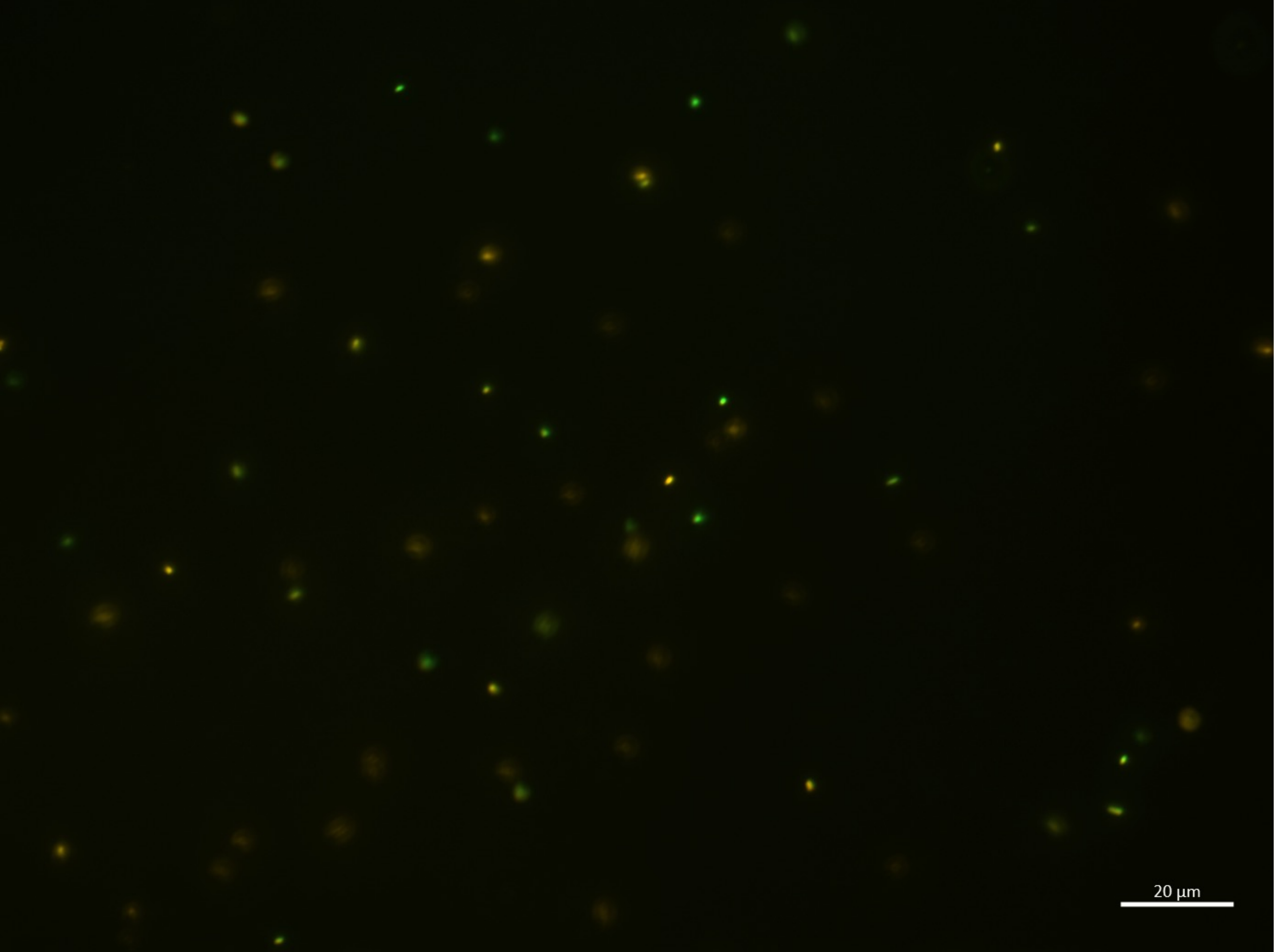

20:00-C

**B.bifidum**	**55**
**E.coli**	13
**Sum**	68

20:00-D

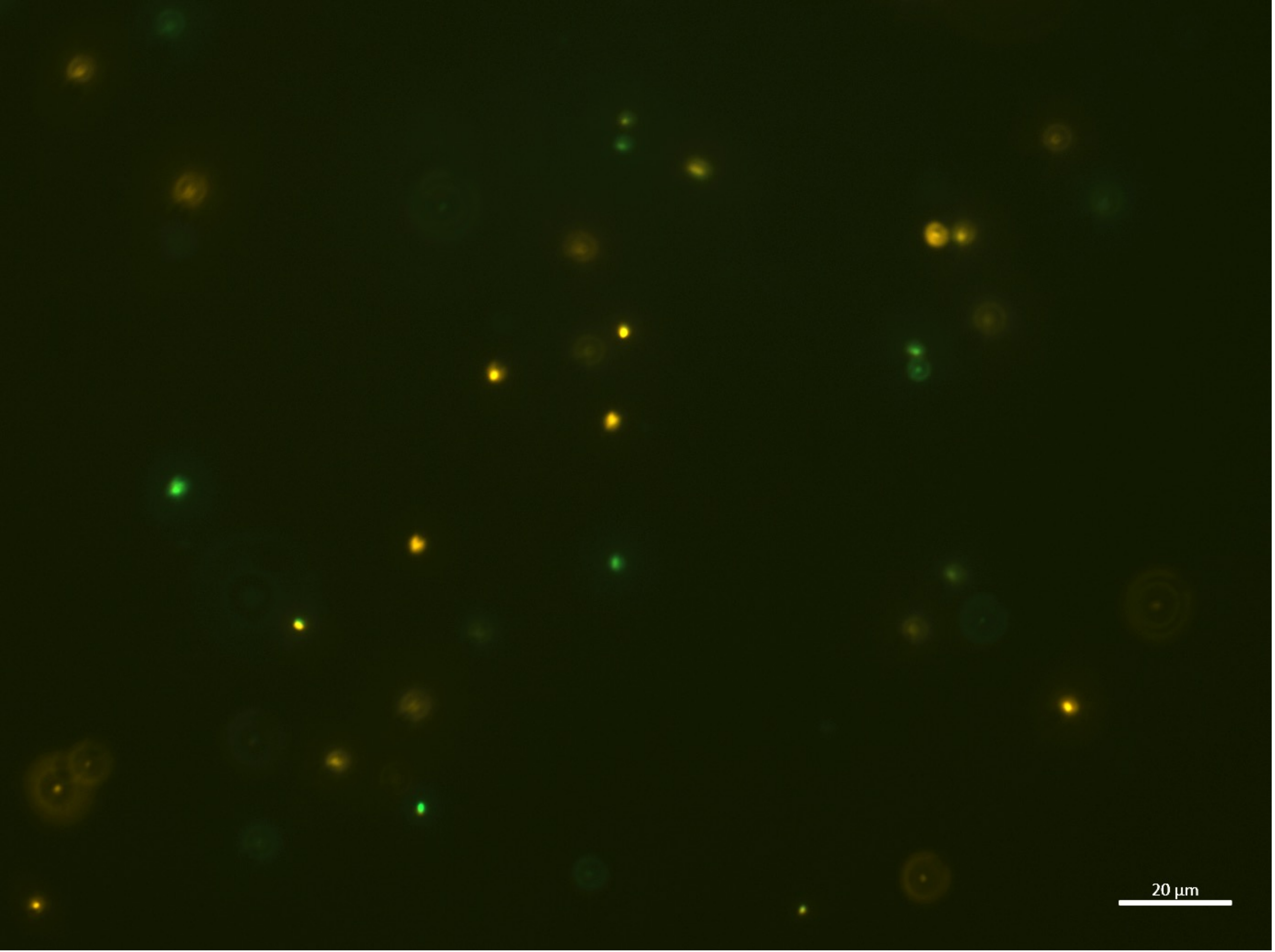

B.bifidum	**20**
E.coli	6
**Sum**	26

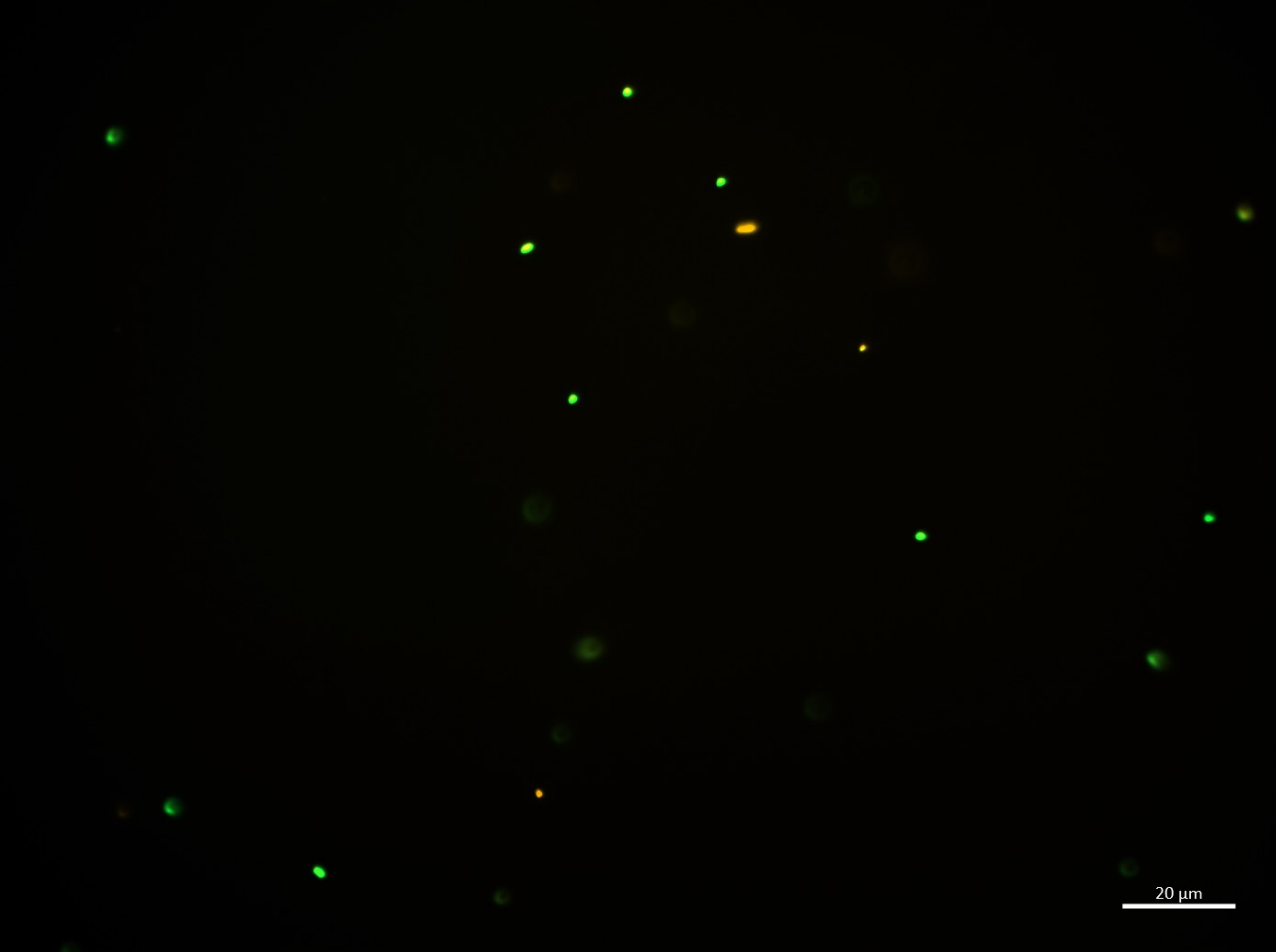

20:00-E

B.bifidum	7
E.coli	10
Sum	17

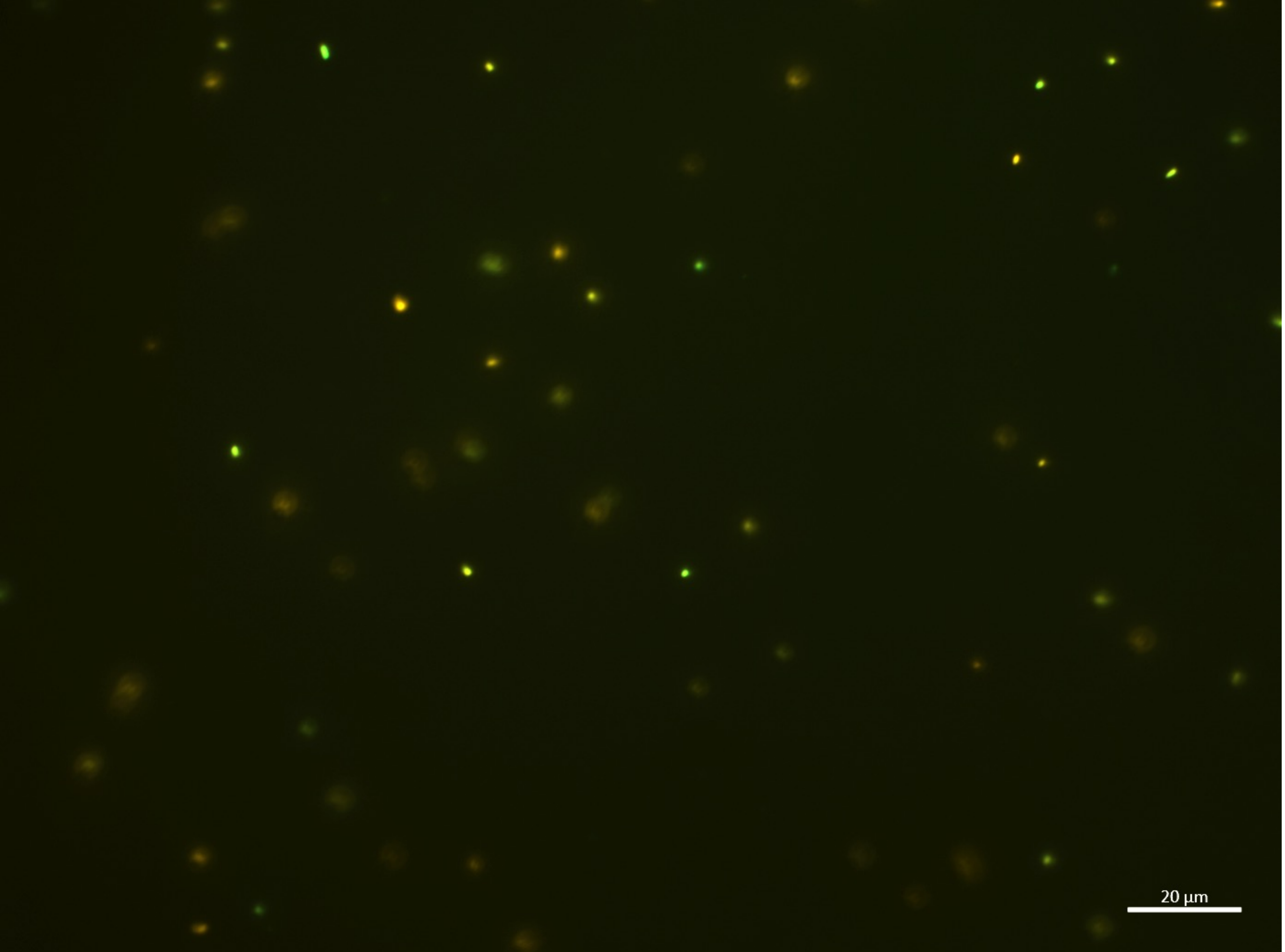

20:00-F

B.bifidum	**36**
E.coli	10
**Sum**	46

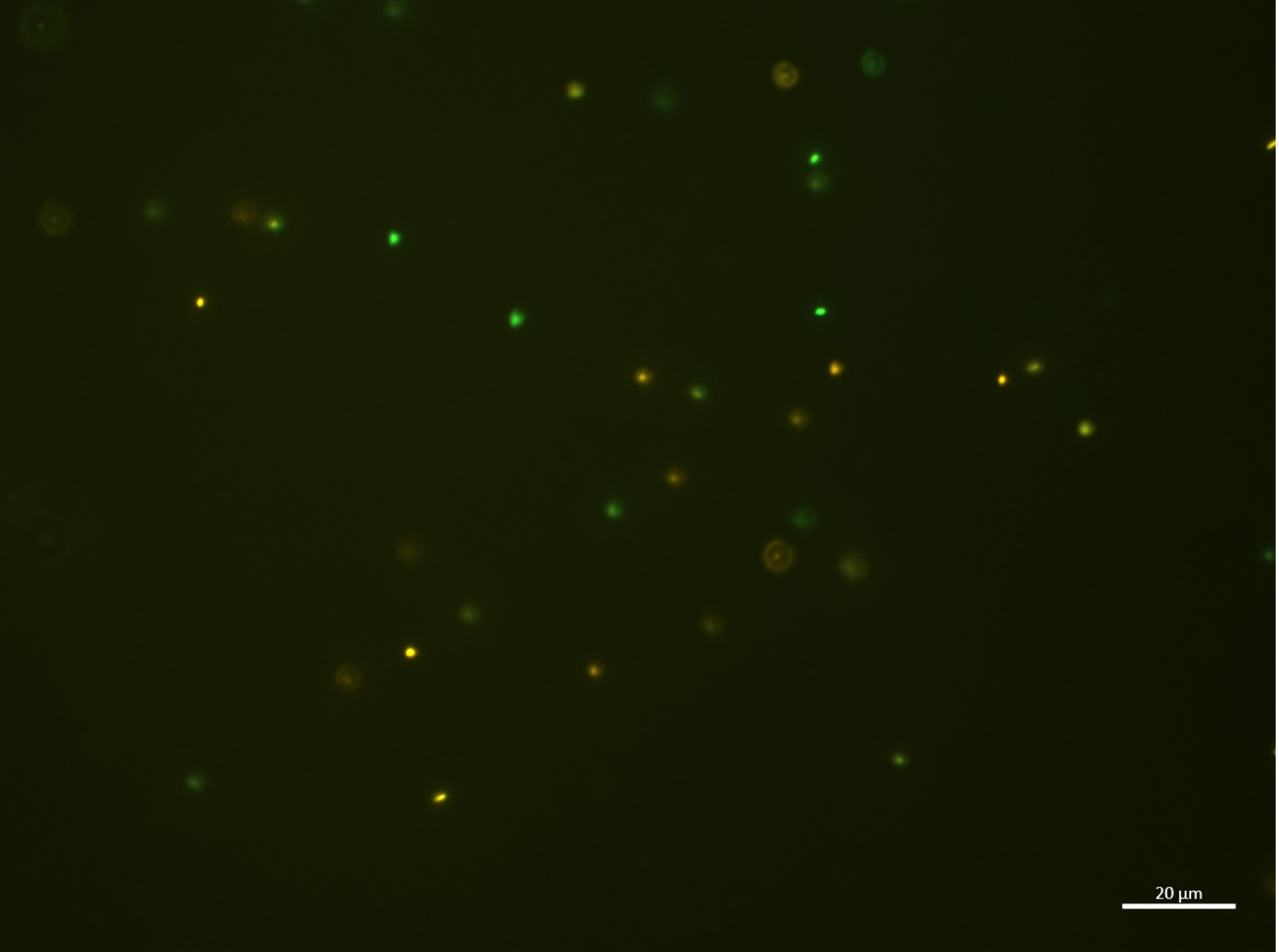

20:00-G

B.bifidum	**22**
E.coli	12
**Sum**	34

20:00-H

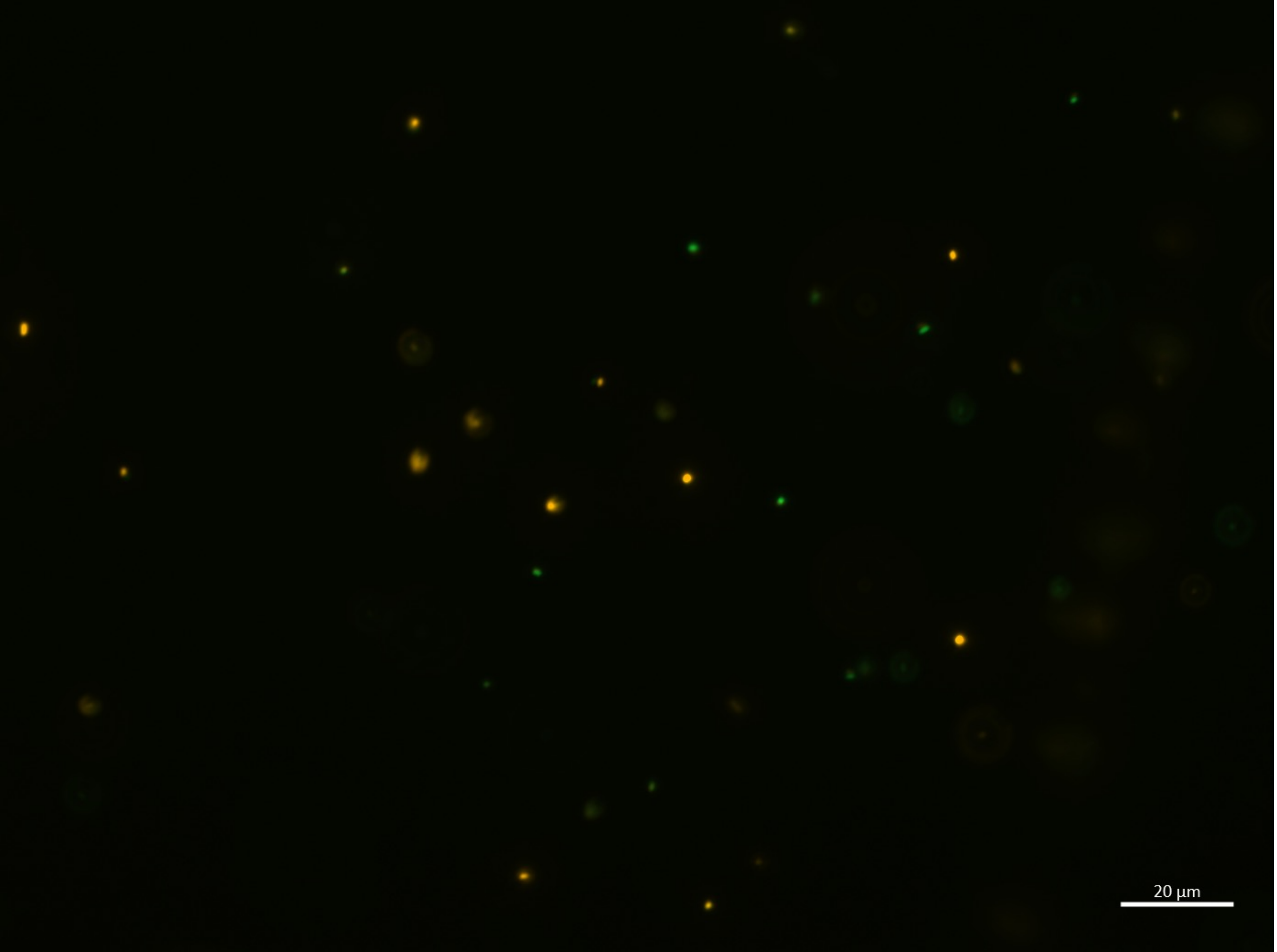

B.bifidum	**25**
E.coli	10
**Sum**	35

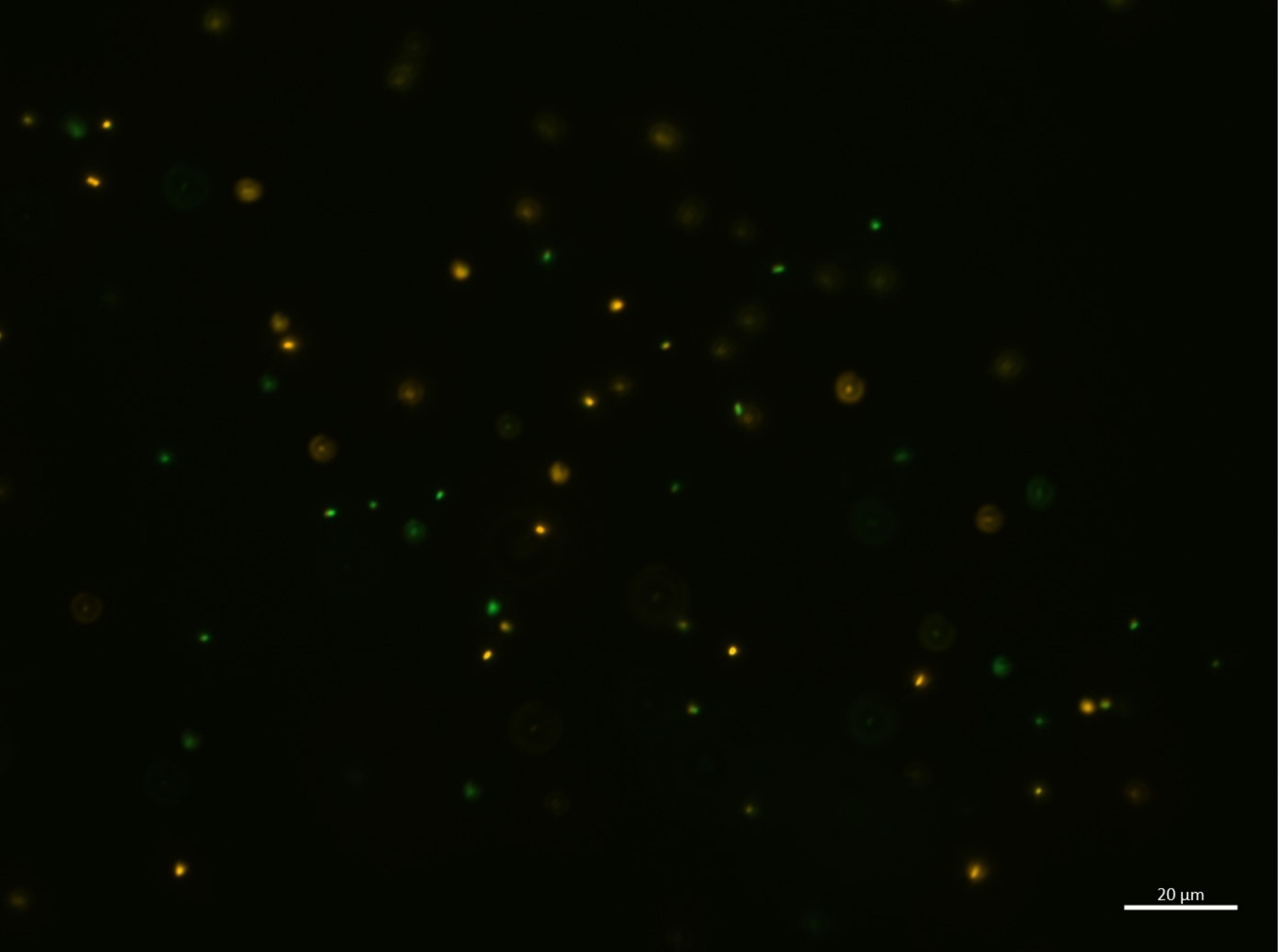

20:00-I

B.bifidum	**50**
E.coli	18
**Sum**	64

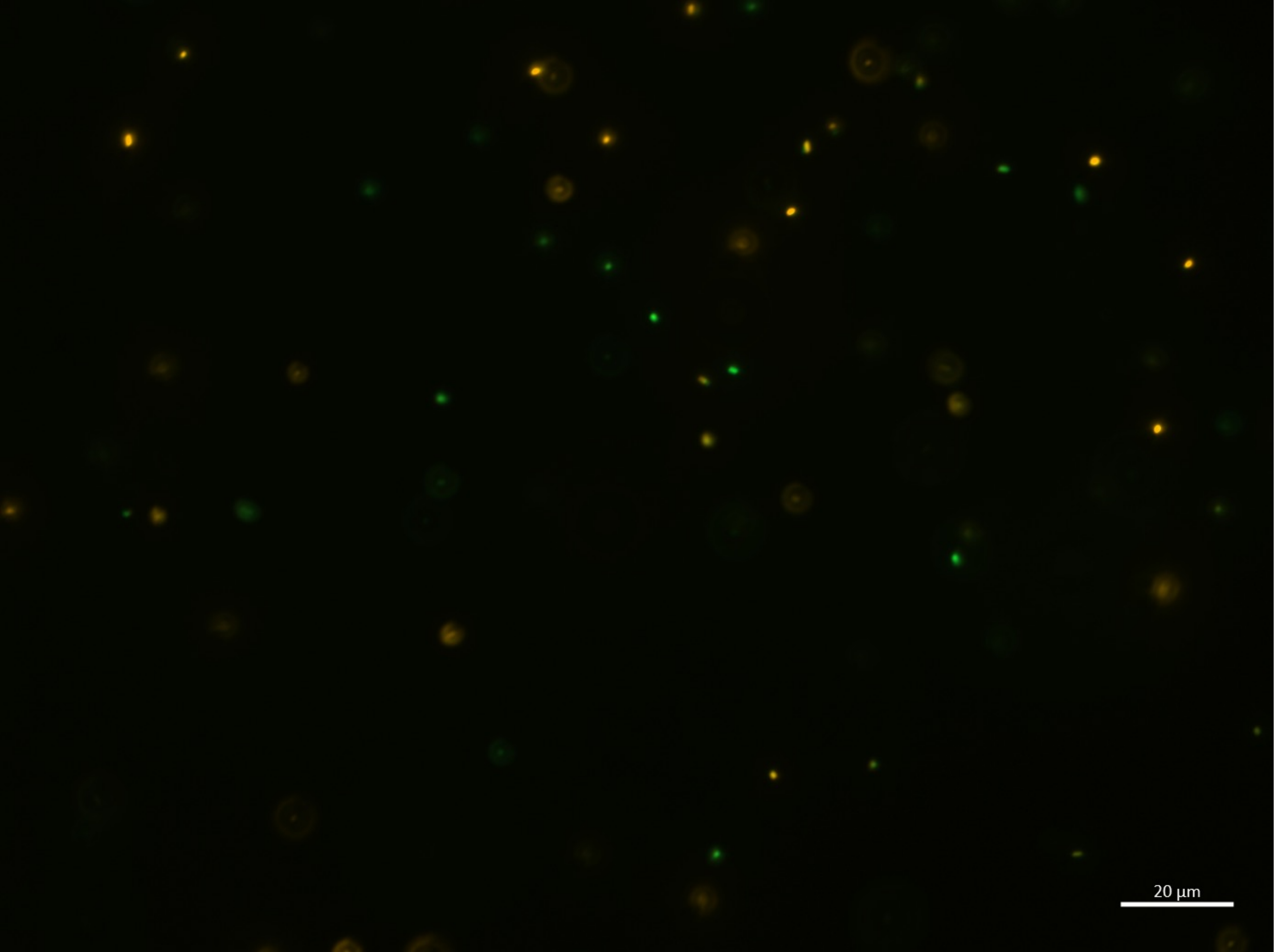

20:00-J

B.bifidum	**38**
E.coli	13
**Sum**	51

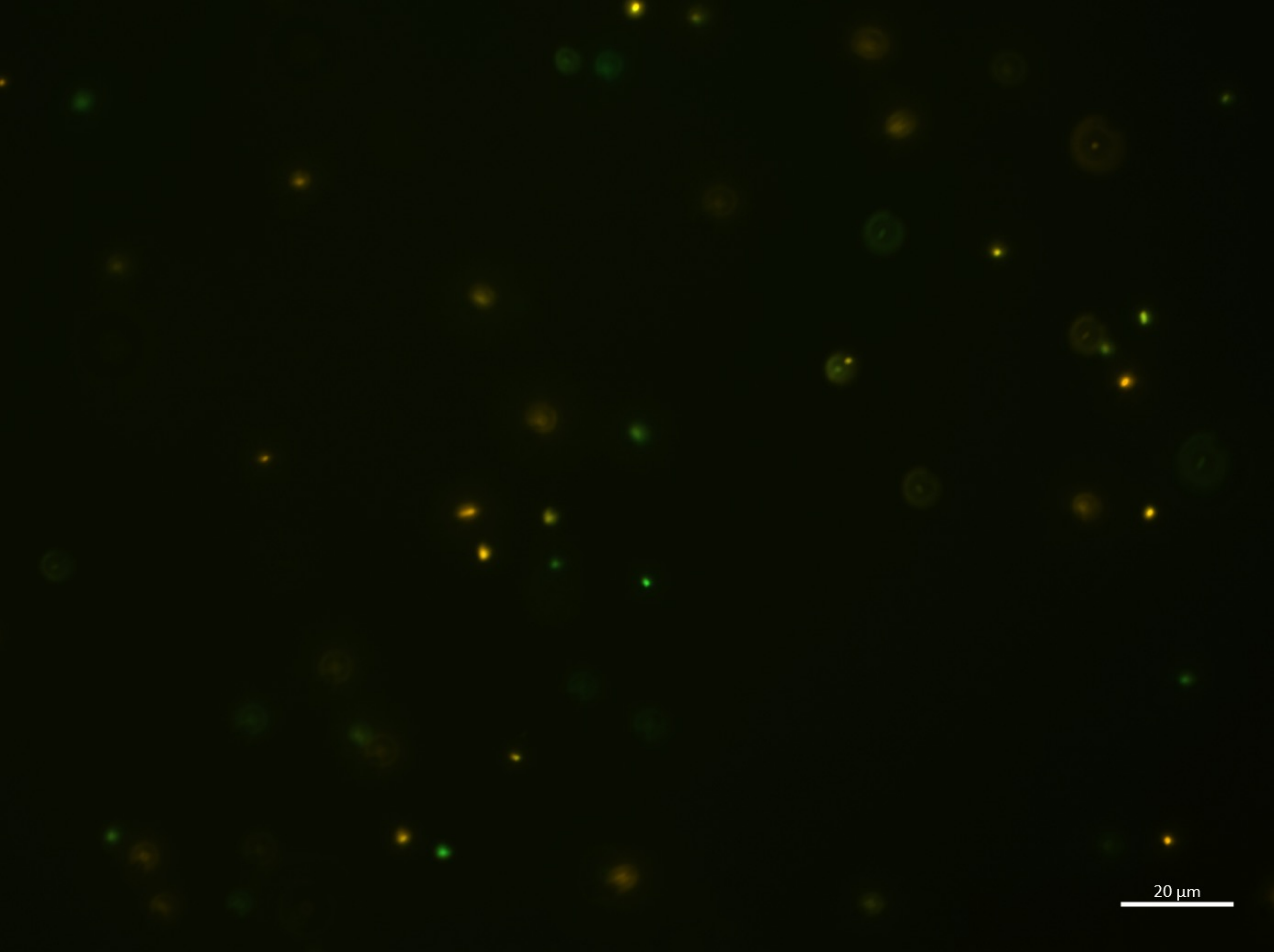

20:00-K

**B.bifidum**	**36**
E.coli	12
**Sum**	48

20:00-L

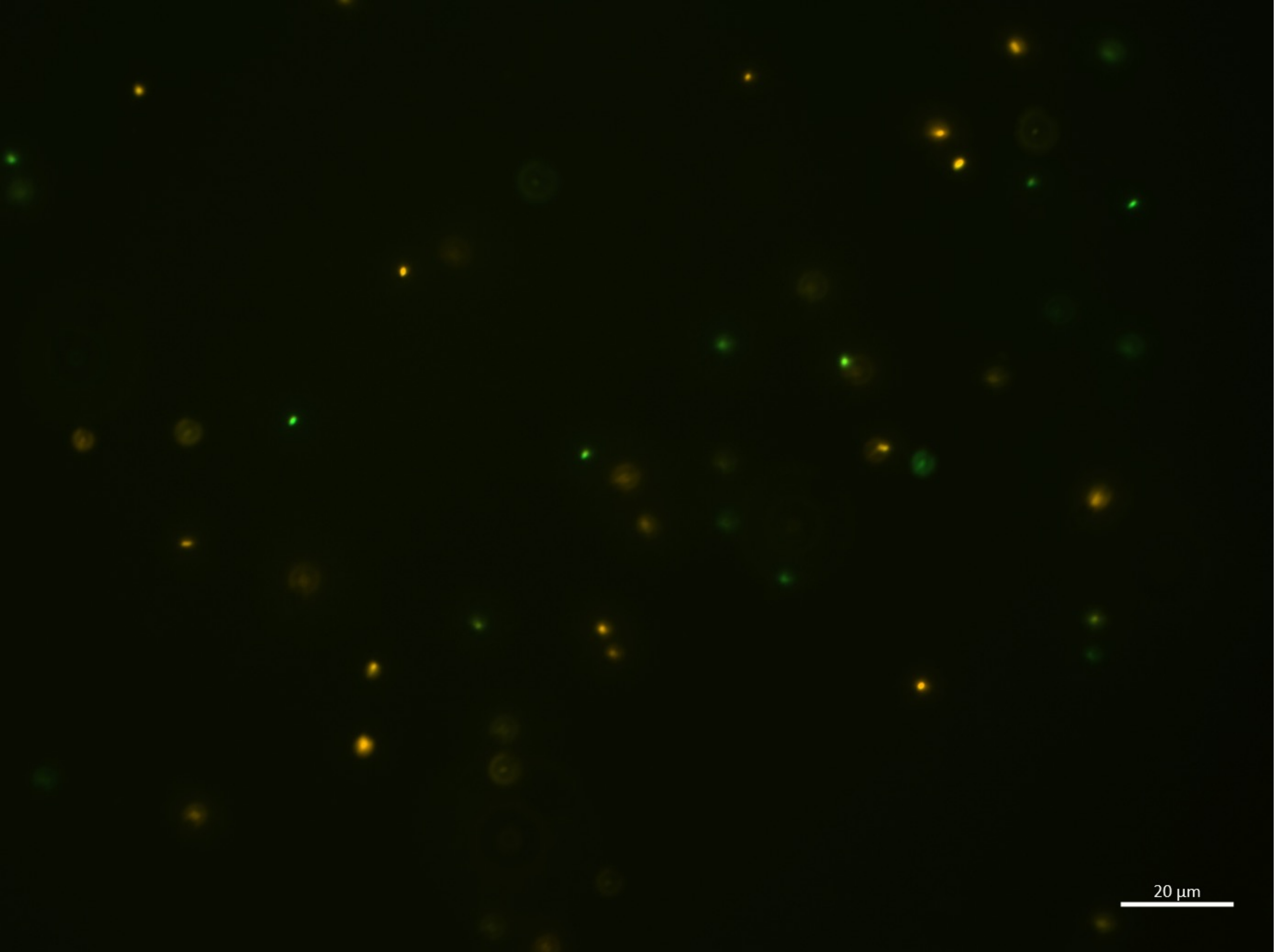

B.bifidum	**33**
E.coli	13
**Sum**	46

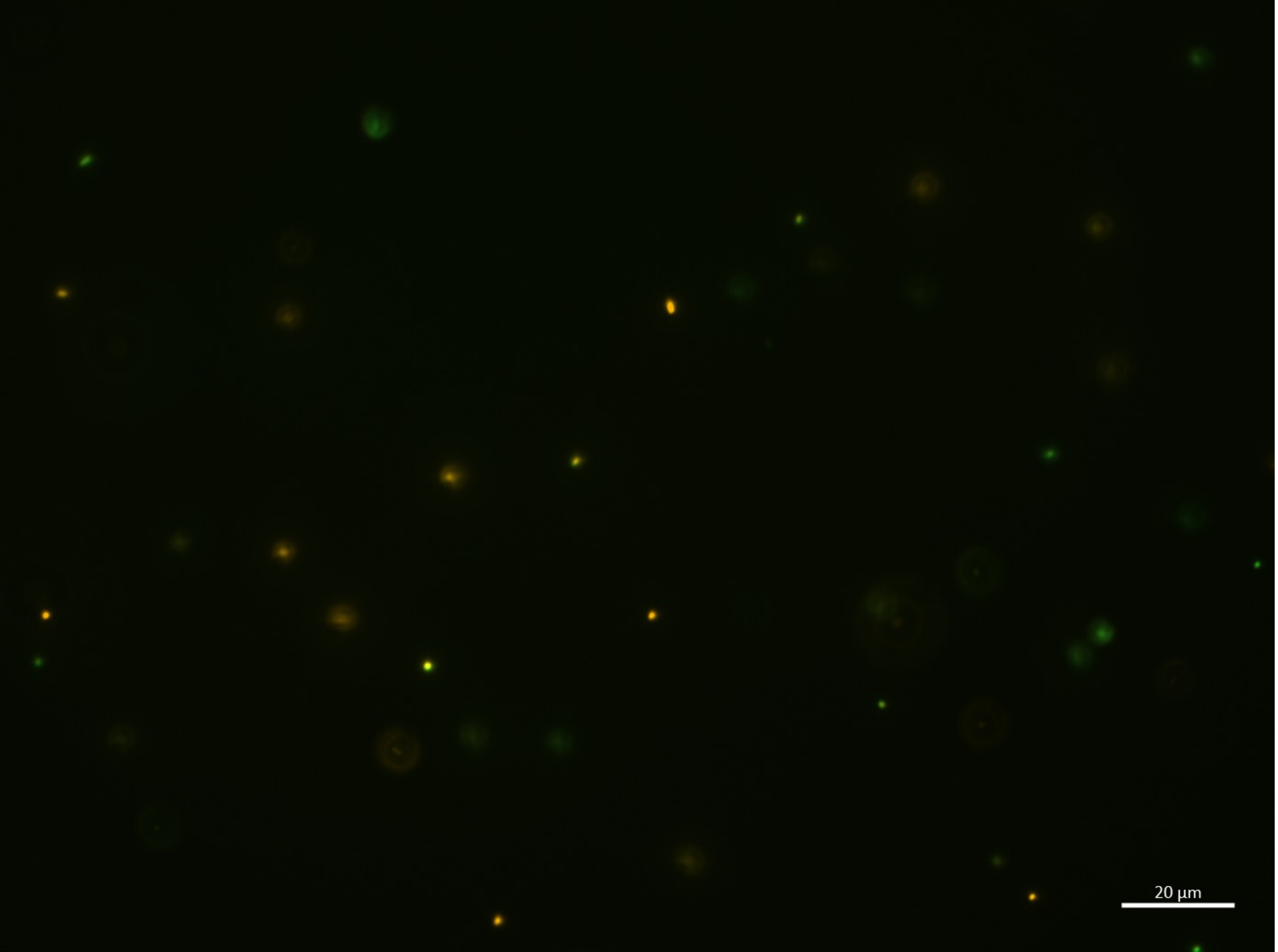

20:00-M

**B.bifidum**	**29**
**E.coli**	6
**Sum**	35

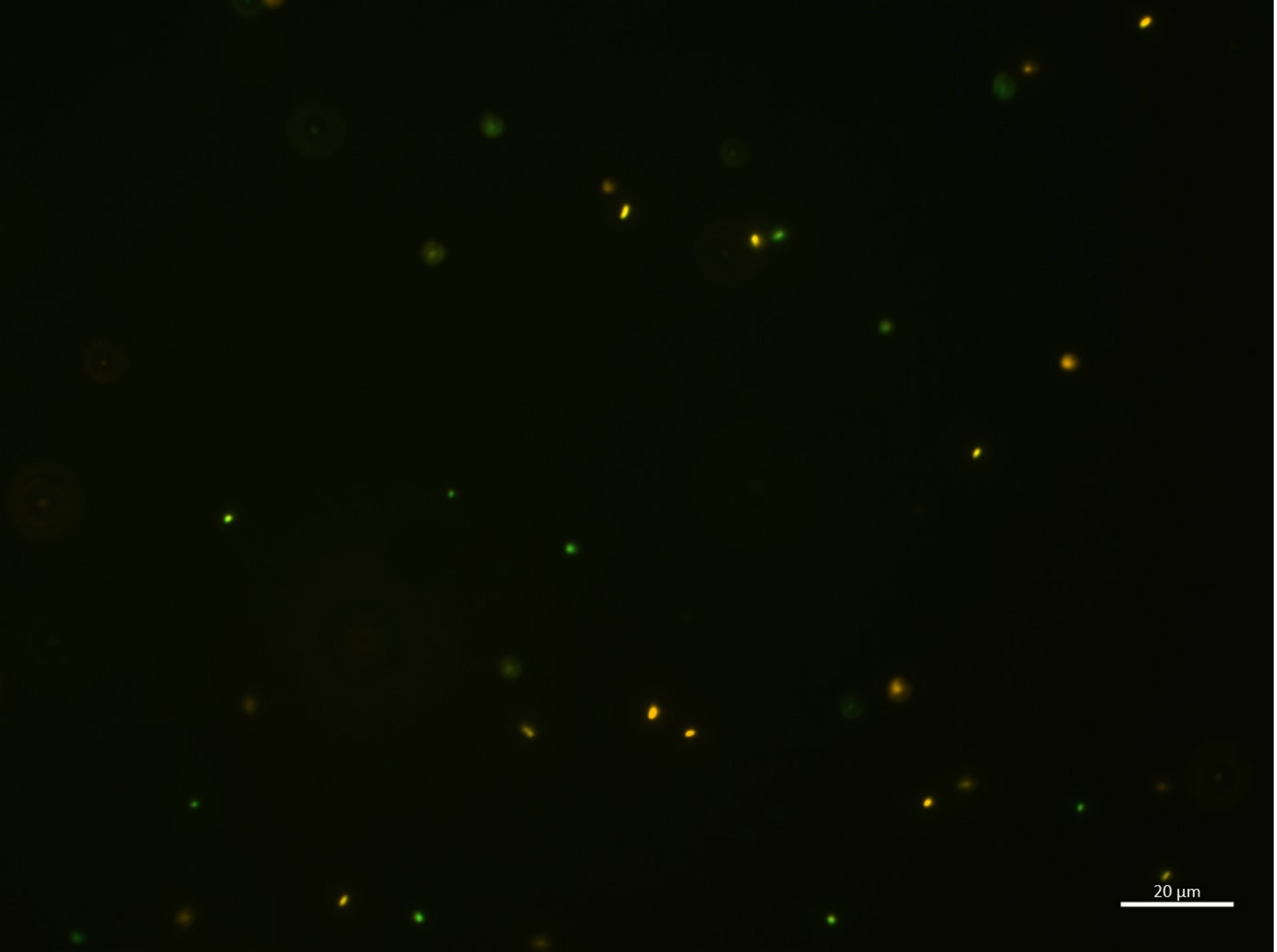

20:00-N

B.bifidum	**24**
E.coli	11
**Sum**	35

20:00-O

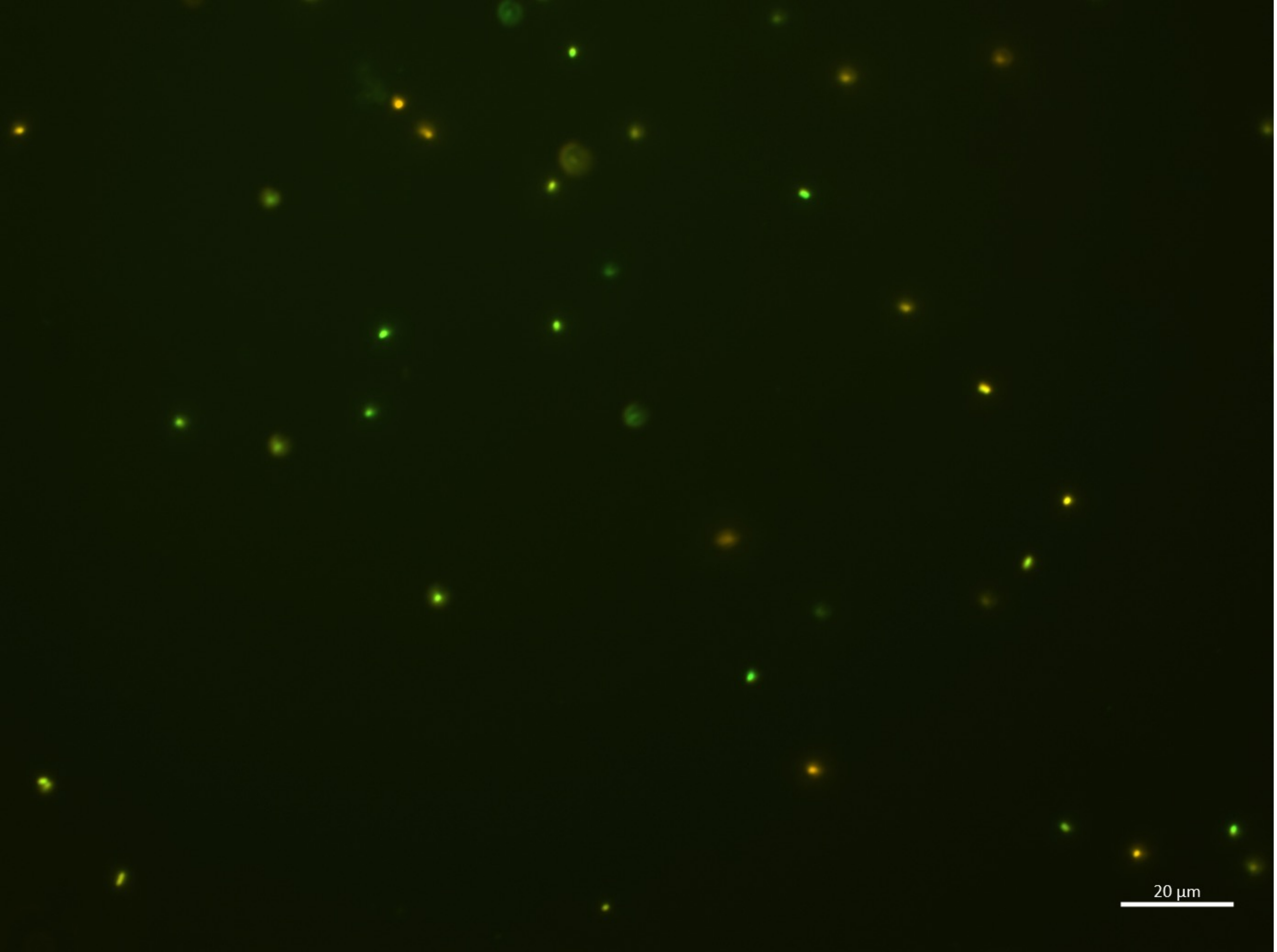

B.bifidum	**22**
E.coli	13
**Sum**	35

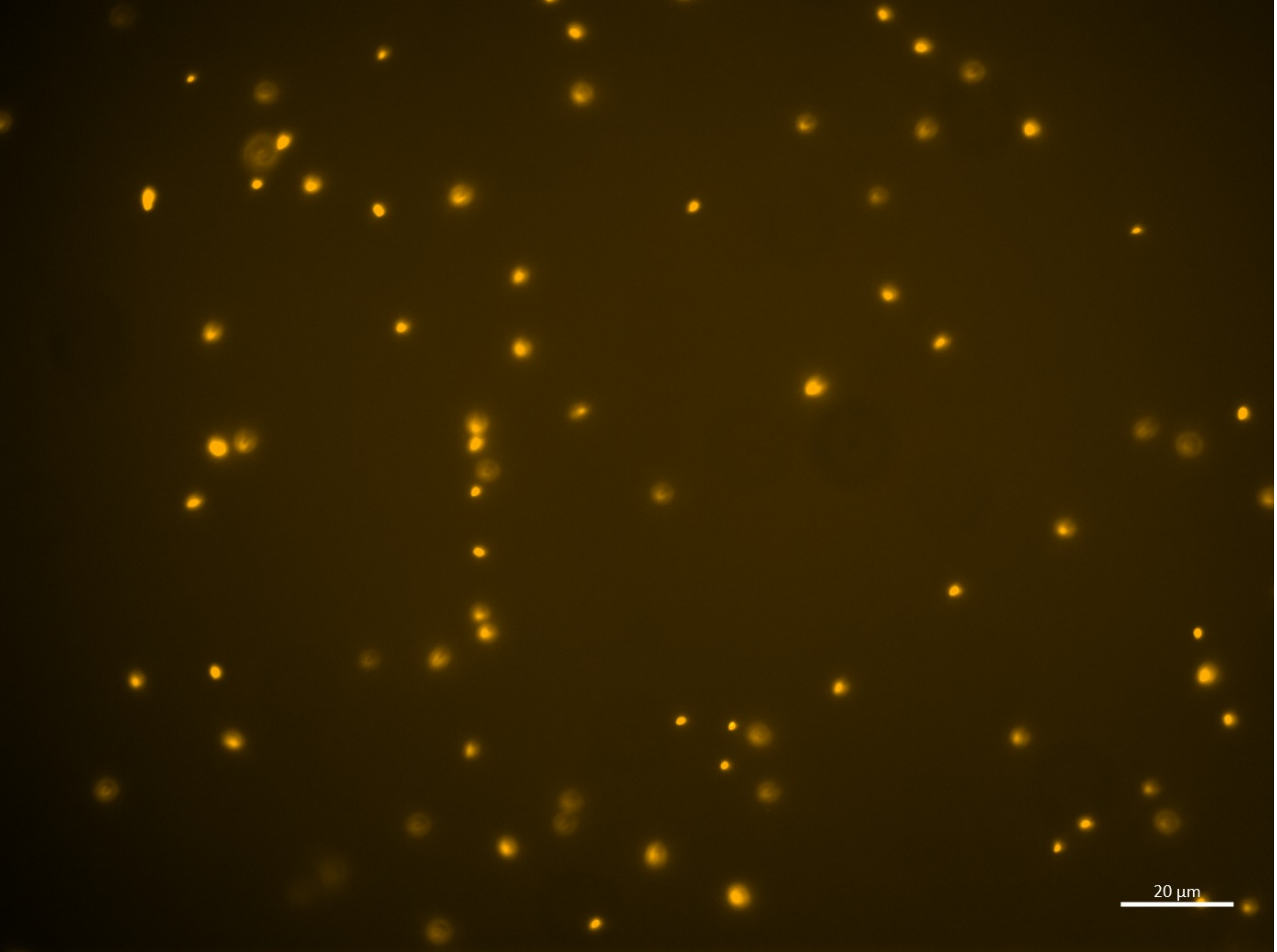

24:00-A

B.bifidum	
E.coli	
Sum	

B.bifidum	100 %

24:00-A

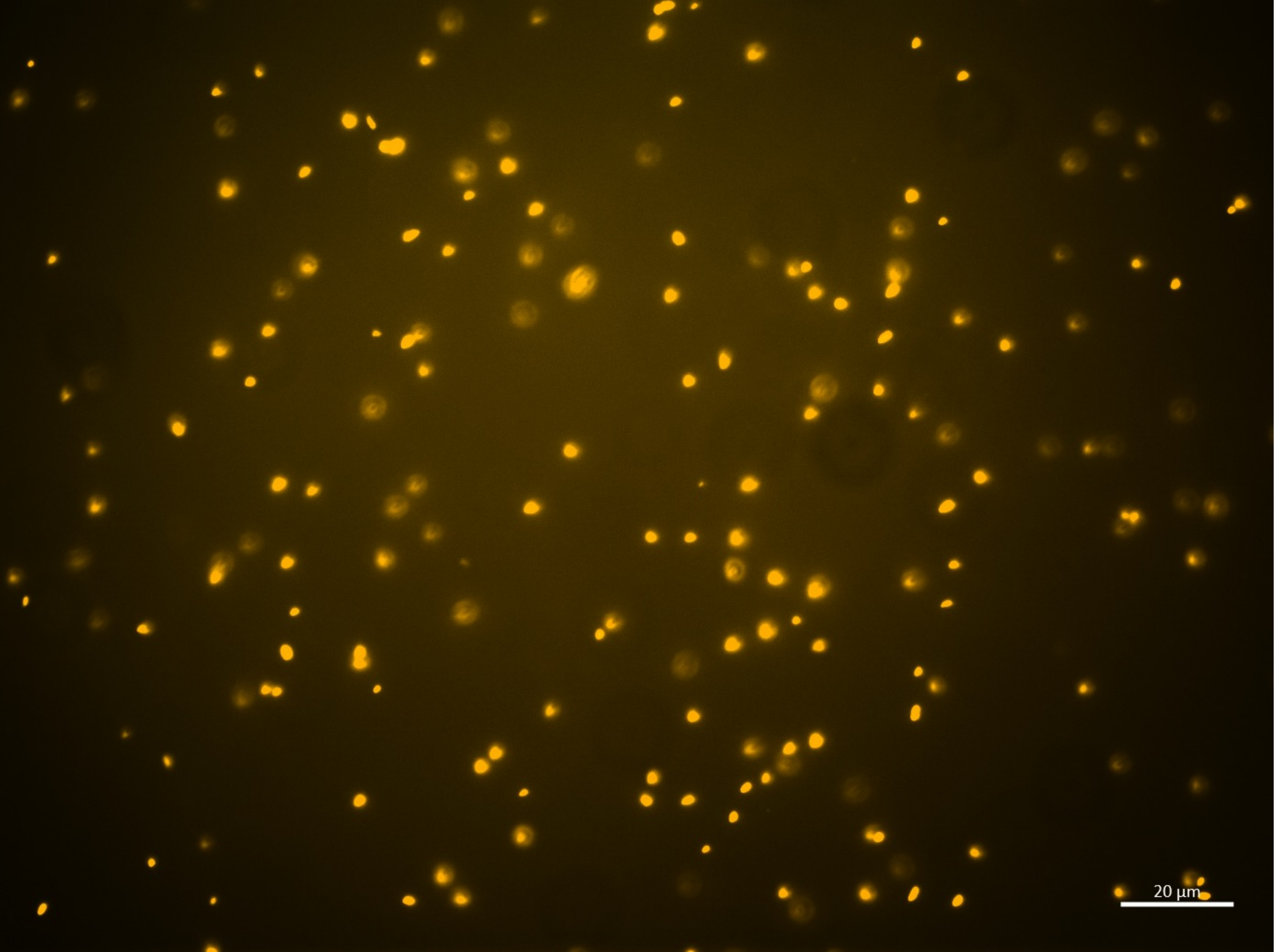

B.bifidum	
E.coli	
Sum	

B.bifidum	100 %

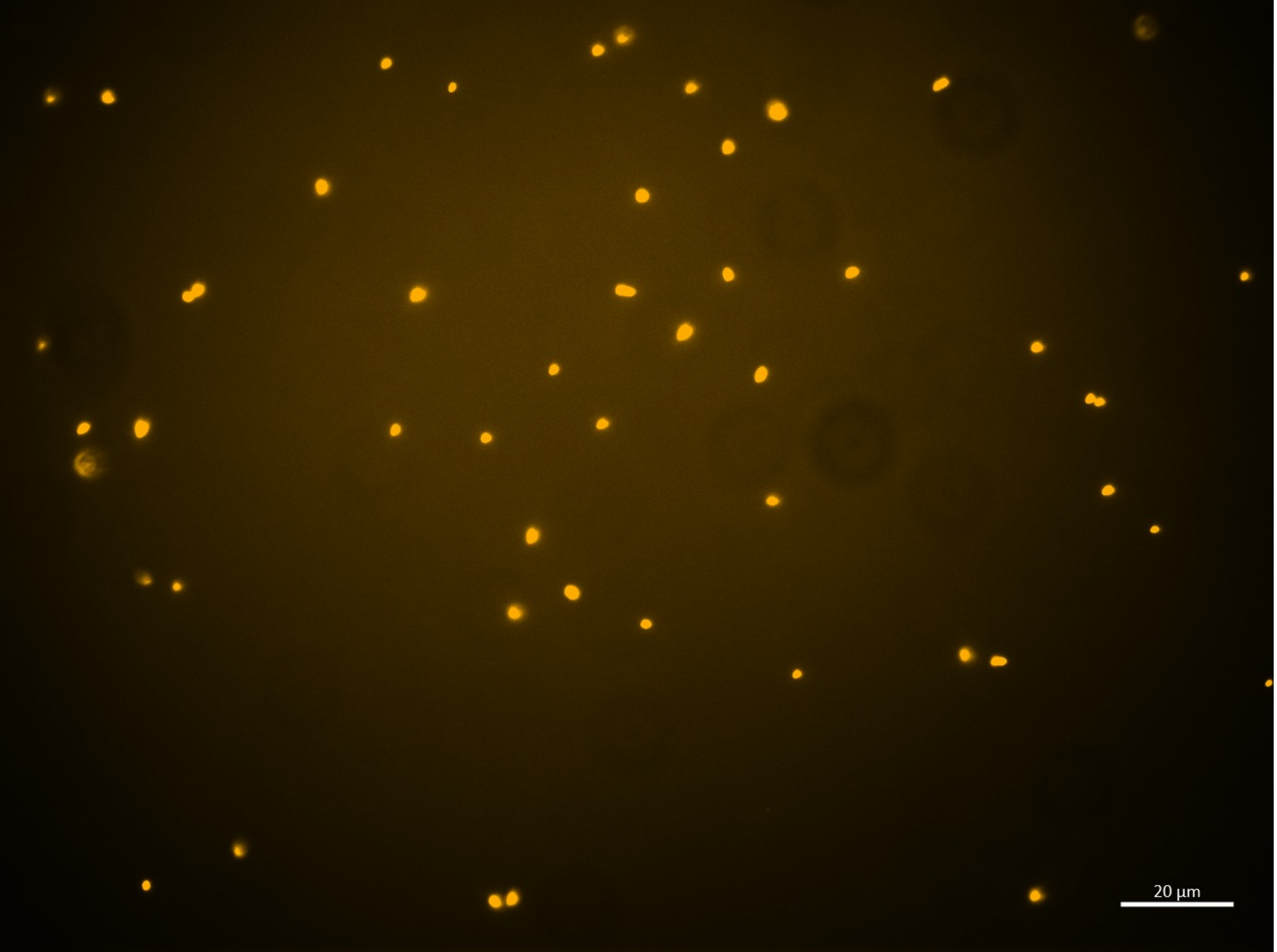

24:00-A

B.bifidum	
E.coli	
Sum	

B.bifidum	100 %

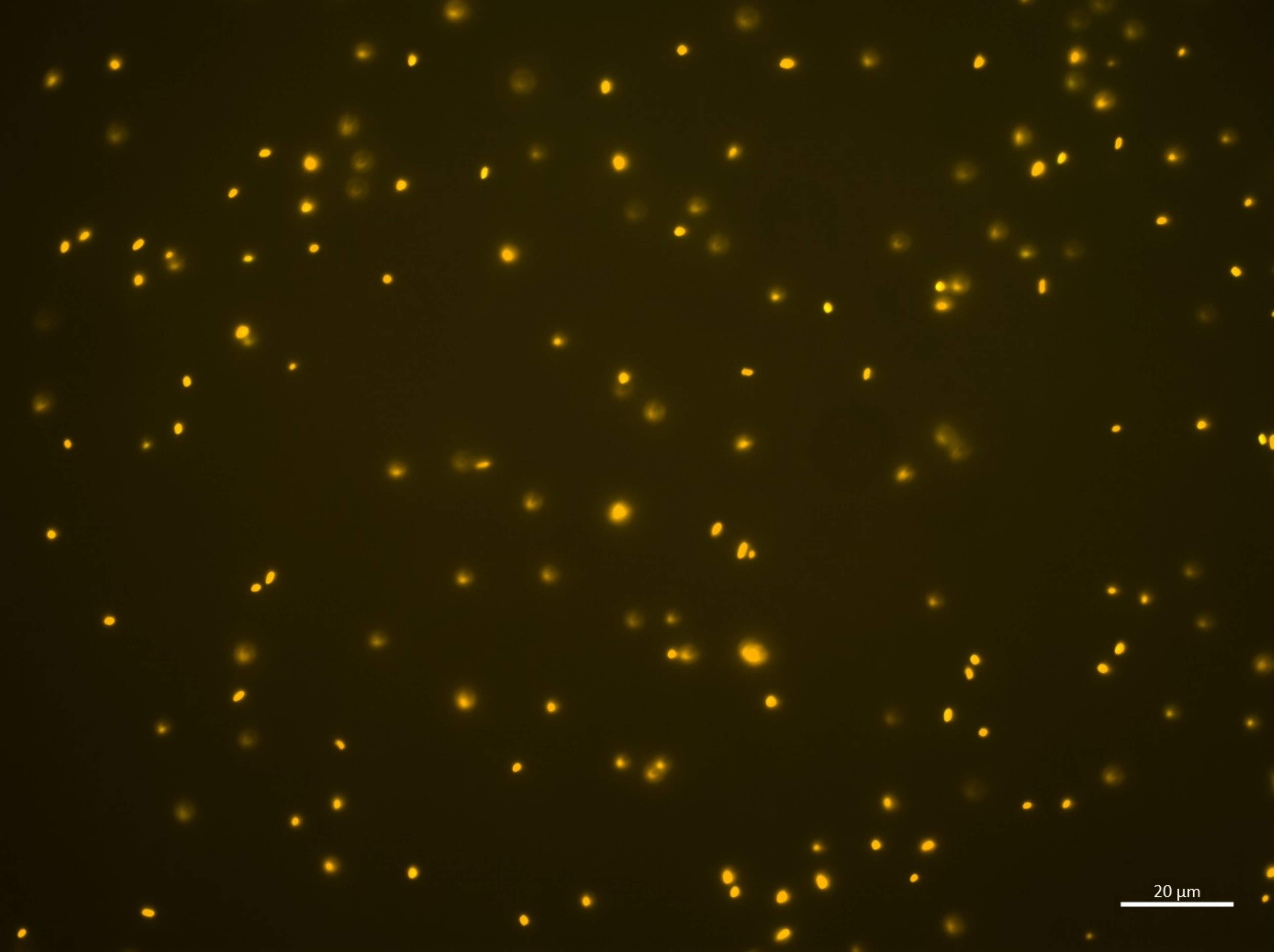

24:00-A

B.bifidum	
E.coli	
Sum	

B.bifidum	100 %

24:00-A

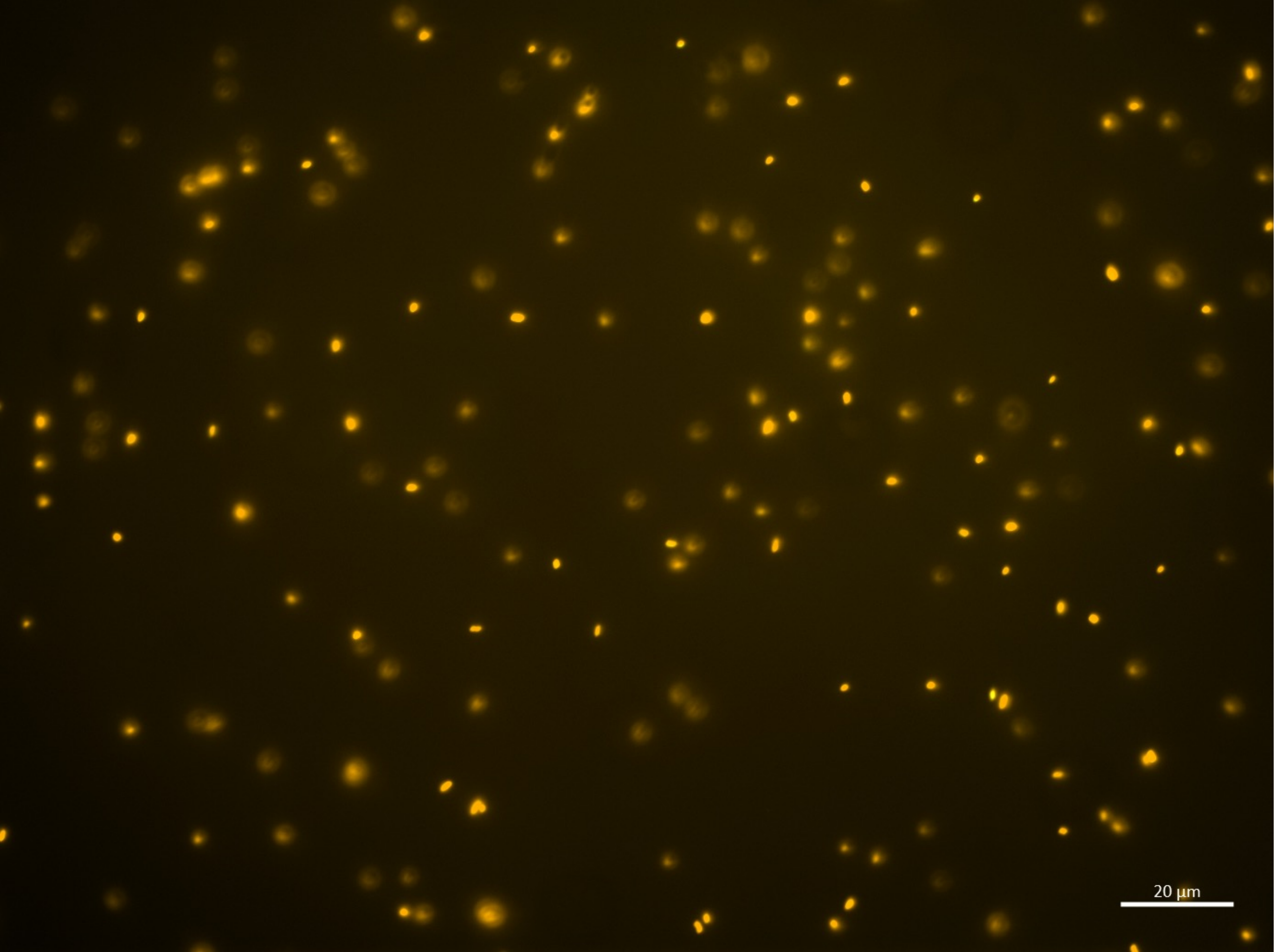

B.bifidum	
E.coli	
Sum	

B.bifidum	100 %

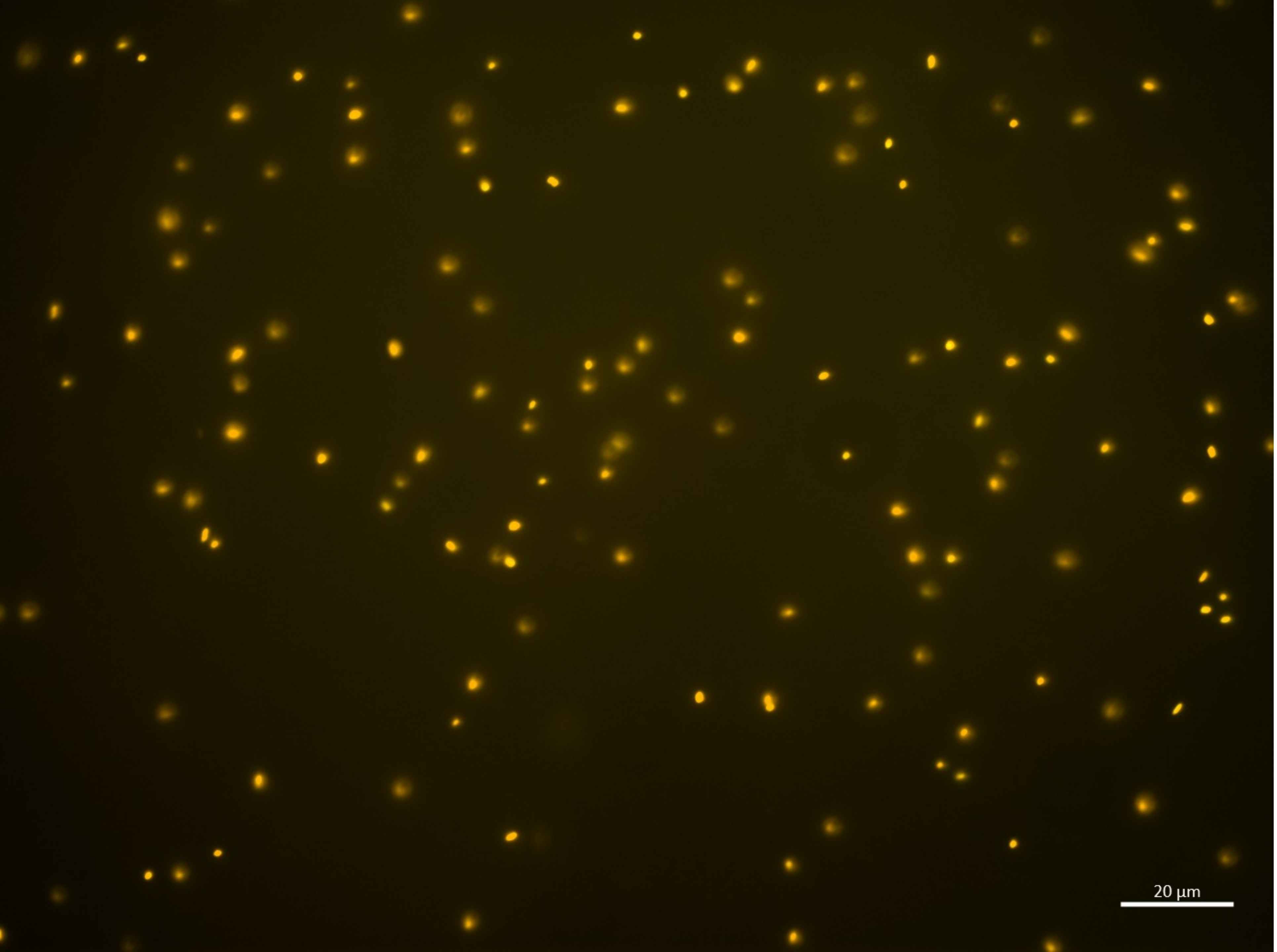

24:00-A

B.bifidum	
E.coli	
Sum	

B.bifidum	100 %

24:00-A

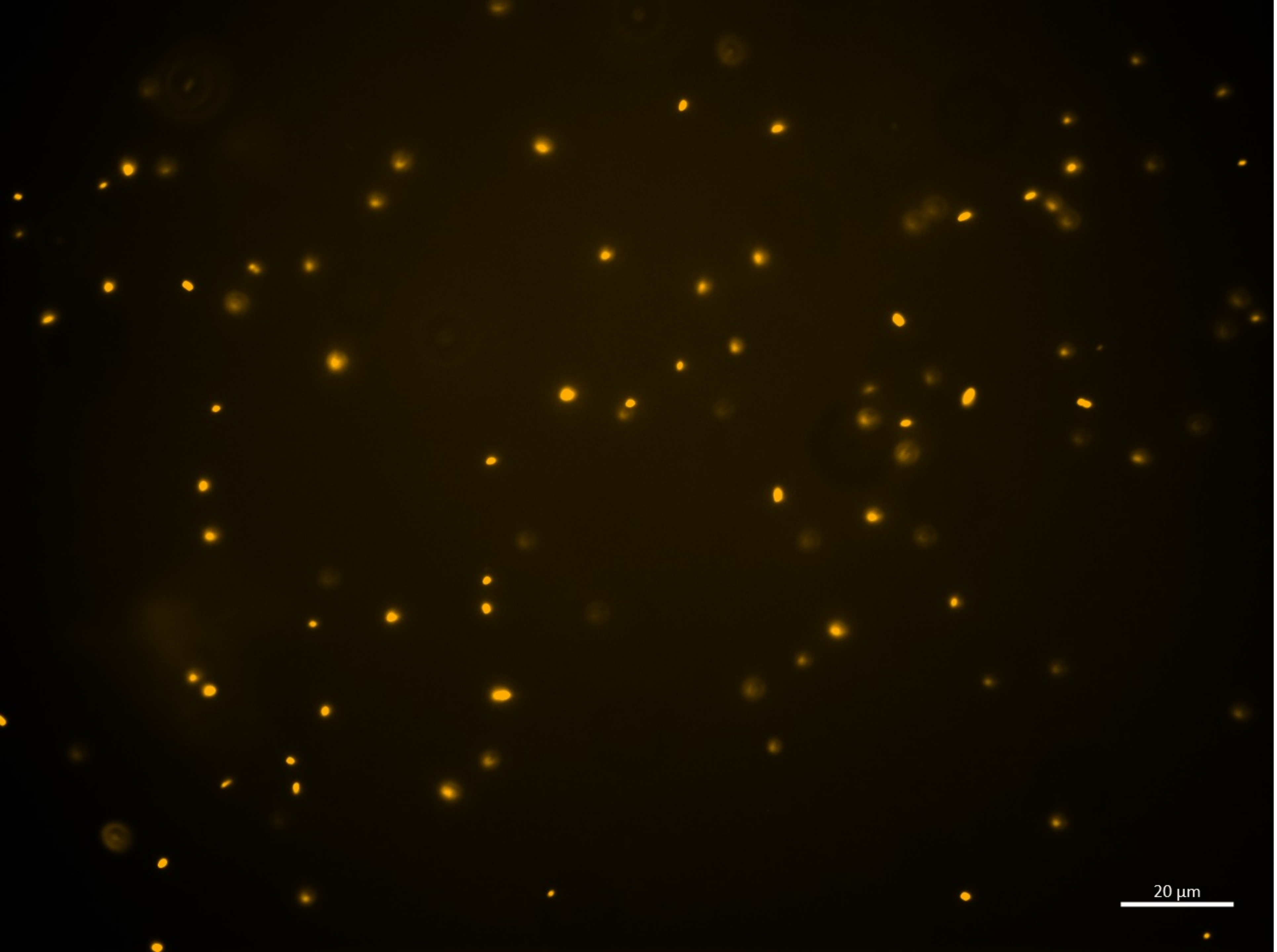

B.bifidum	
E.coli	
Sum	

B.bifidum	100 %

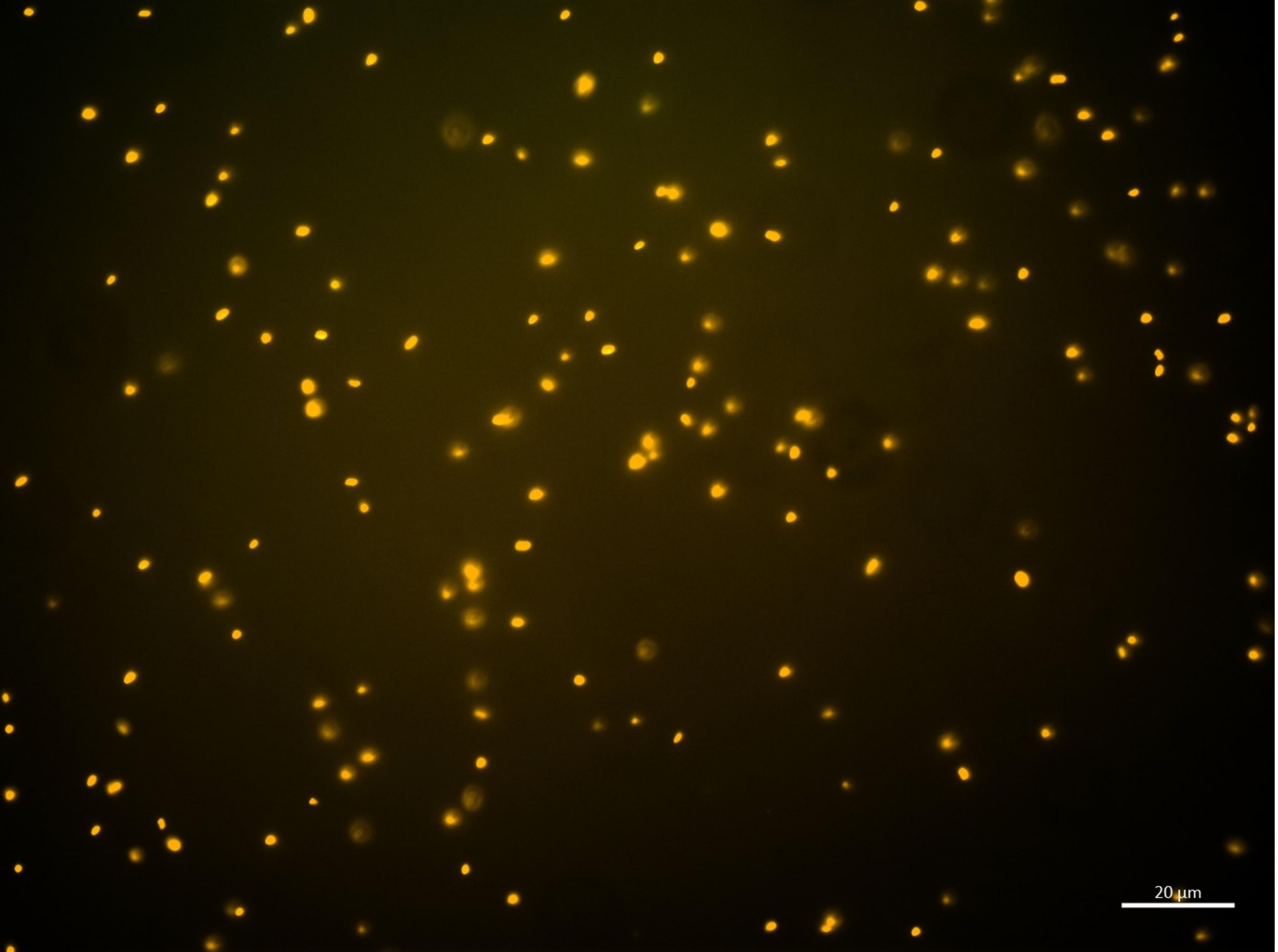

24:00-A

B.bifidum	
E.coli	
Sum	

B.bifidum	100 %

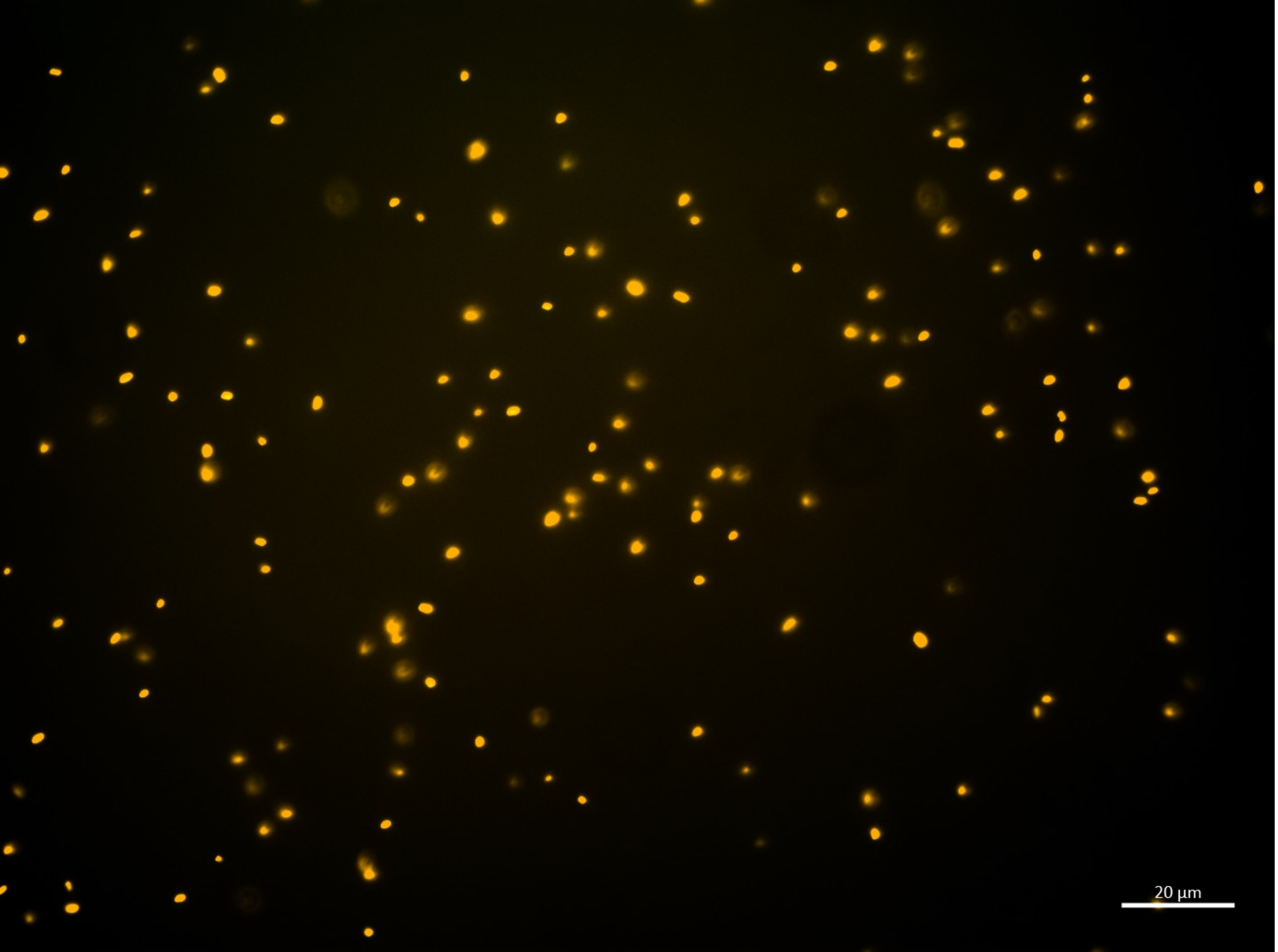

24:00-A

B.bifidum	
E.coli	
Sum	

B.bifidum	100 %
